# Chirality-induced magnetoresistance in hybrid organic-inorganic perovskite semiconductors

**DOI:** 10.1126/sciadv.aed7176

**Published:** 2026-09-09

**Authors:** Md Azimul Haque, Pius M. Theiler, Ian A. Leahy, Steven P. Harvey, Jeiwan Tan, Matthew P. Hautzinger, Margherita Taddei, Aeron McConnell, Andrew Grieder, Andrew H. Comstock, Yifan Dong, Kirstin Alberi, Yuan Ping, Peter C. Sercel, Joseph M. Luther, Dali Sun, Matthew C. Beard

**Affiliations:** ^1^ National Laboratory of the Rockies, Golden, CO, USA.; ^2^ RASEI: The Joint CU-Boulder/NLR Energy Institute, Boulder, CO, USA.; ^3^ Department of Physics and Organic and Carbon Electronics Lab (ORaCEL), North Carolina State University, Raleigh, NC, USA.; ^4^ Department of Materials Science and Engineering, University of Wisconsin-Madison, Madison, WI, USA.; ^5^ Department of Physics, University of Wisconsin-Madison, Madison, WI, USA.; ^6^ Department of Chemistry, University of Wisconsin-Madison, Madison, WI, USA.; ^7^ Center for Hybrid Organic Inorganic Semiconductors for Energy, Golden, CO, USA.

## Abstract

The combination of semiconducting properties and synthetically tunable chirality in chiral metal halide semiconductors (CMHSs) offers a compelling platform for room temperature control of electronic spin properties, leveraging effects such as chirality-induced spin selectivity (CISS). We report CISS-induced magnetoresistance (CISS-MR) exceeding 100% for spin valves operating at room temperature. The high CISS-MR is attributed to an interfacial tunneling barrier, modulated by chirality and spin, producing current dissymmetry factors (*g*_c_) that surpass the limit imposed by the Julliere model, which is governed by the intrinsic spin polarization of the adjacent ferromagnetic (FM) contact. The CISS-MR exhibits a dependence on the CMHS composition, revealing structure-property relationships between CISS and structural chirality. The observed exceptionally large tunneling MR response differs from a subtle anisotropic MR arising from the proximity effect at the FM/CMHS interface in the absence of a tunneling barrier. Our study provides insights into charge-to-spin interconversion in chiral semiconductors, offering design principles to control and enhance the CISS response for functional platforms.

## INTRODUCTION

Metal halide semiconductors (MHS) are widely studied for optoelectronic applications due to their tunable semiconducting properties. The hybrid organic/inorganic nature of MHS enables properties that are not available to either purely organic or inorganic systems. The prototypical realization of such emergent properties is in the incorporation of chiral organic cations, producing a family of enantiomerically pure chiral semiconductors (CMHS) with novel functionality whose properties can be tuned through harnessing the synergistic interactions between the chiral organic and achiral inorganic components. In CMHSs, chiral organic molecules incorporated as A-site cations distort the metal halide octahedra through asymmetric hydrogen bonding interactions leading to symmetry breaking (*1*). This enables phenomena such as the circular photogalvanic effect, the large spin Hall effect, the chiral phonon–activated Seebeck effect, and the chirality-induced spin selectivity (CISS) effect (*2*).

CISS enables control over spin populations at room temperature without requiring magnetic fields or ferromagnets providing a fundamentally new route to achieve spin functionality (*3*). The CISS effect, when realized in a chiral semiconductor, forms the basis for various chiral-optoelectrical applications such as circularly polarized light detection, spin light-emitting diodes, and chiral spin valves (*4*–*6*). A spin valve is a simple two-terminal device that exhibits two distinct resistance states depending on the relative alignment of its magnetic layers, termed magnetoresistance (MR). Unlike conventional spin valves requiring two ferromagnetic (FM) electrodes, or an FM and a hard magnetic electrode, CISS-based spin valves operate with a single soft FM, and the two resistance states are determined by the alignment between the magnetic polarization and stereochemical configuration. As a result, CISS-based spin valves operate through a new mechanism (chirality) whose understanding and control could pave the way for new unrealized functionality not available using traditional approaches. While CISS has been observed in DNA, peptides, self-assembled monolayers, and chiral quantum dots (*7*), understanding its structure-property relationships remains challenging due to the limited tunability of existing CISS-active thin films and general fabrication methods. CMHSs with composition A_2_BX_4_, where A is an organic chiral cation, B is the metal cation, and X is a halide anion provide an ideal platform to overcome this limitation, offering synthetic control over semiconducting properties via B and X sites and symmetry broken crystal structures via A-site chirality, making them promising candidates for CISS-based applications and for developing an understanding of the underlying CISS mechanisms (*8*–*11*).

Here, we developed a chiral spin valve based on a tunneling junction which exhibits CISS-induced MR (CISS-MR) well in excess of 300% at room temperature, comparable to conventional spin valves, which exceed ∼200% in consumer electronics (*12*). Magnetochiral tunnel barrier modulation has been discussed from both equilibrium electrostatic (*13*) and dissipative transport-induced (*14*) perspectives. Our data are consistent with the electrostatic picture, and we propose a spin-selective charge localization mechanism as the physical origin of the barrier modulation. We show that without the tunneling barrier, CISS-MR values are reduced. The interfaces between the tunneling barrier, FM, and chiral semiconductors play the key role in achieving high MR values reported here. With this platform, we established structure/function relationships by comparing the MR from four different chiral A-site cations that induce varying degrees of chirality, assessed through the continuous chirality measure (CCM) and incorporated into three different spin valve architectures. Our findings provide design principles to enhance and understand charge-to-spin interconversion in chiral semiconductors, offering disorder-tolerant, high-MR spin functionality. Similar to the success envisioned with solution-processed halide perovskites in potentially revolutionizing solar cell technology, CMHSs offer a unique opportunity to merge the structural tunability of perovskite semiconductors with the intrinsic spin selective functionality inherent in chiral systems (*15*).

## RESULTS

### Spin valve fabrication and analysis

**Fig. 1.**
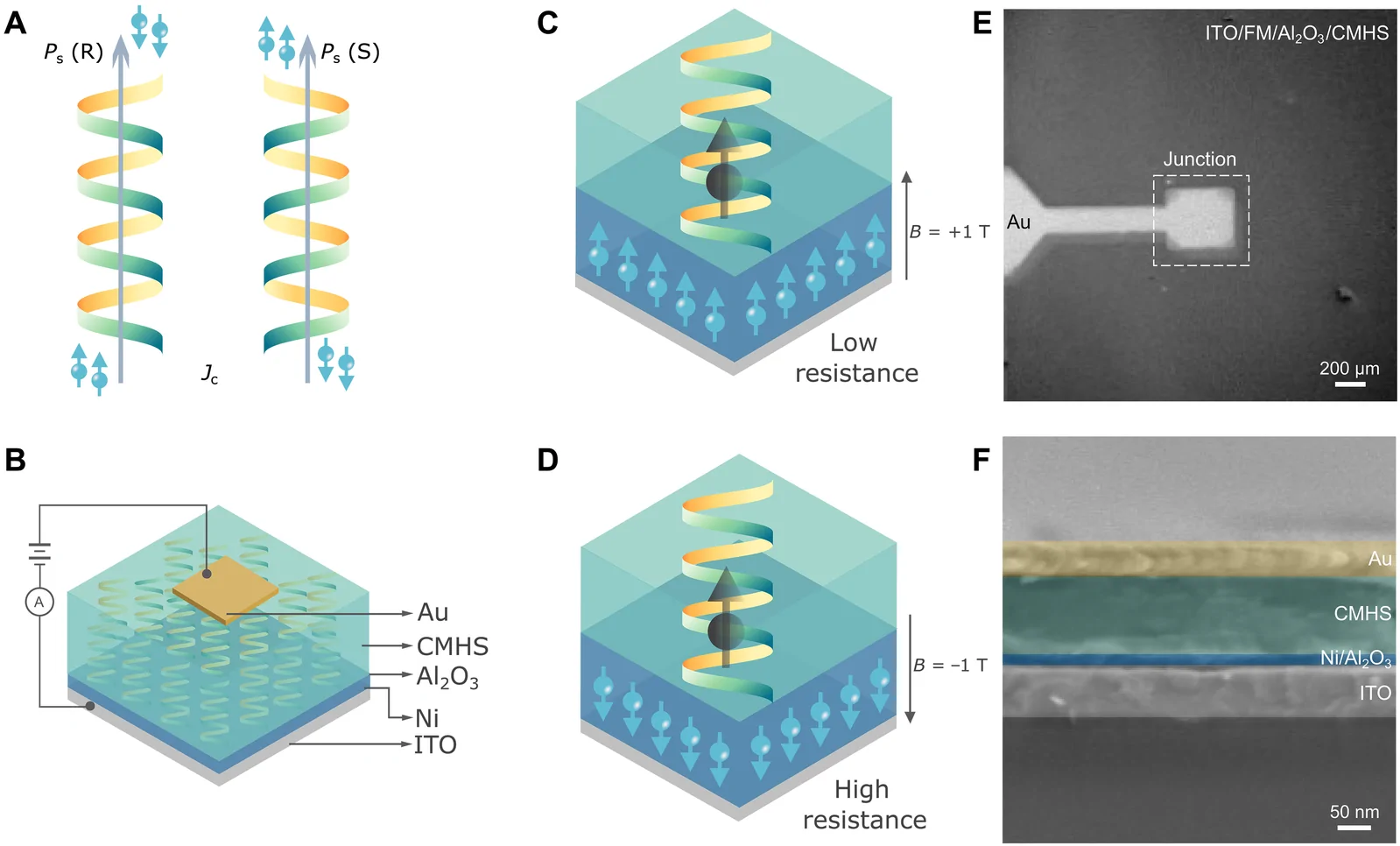
CISS-based spin valve design and response. (**A**) Schematic illustration of the CISS effect, where a flow of charge current *J*_c_ in the enantiomorphs generates a pair of spin polarizations *P*_s_. (**B**) CISS spin valve architecture. CMHS enantiomorph with a preference for spin-up carrier transport separated from a Ni electrode by an Al_2_O_3_ tunnel barrier under out-of-plane magnetic field values of B± = ±1 T. A low resistance (**C**) state is observed when the Ni and enantiomorph spins are parallel and high resistance (**D**) when the spins are antiparallel. SEM images showing (**E**) top view of the spin valve and (**F**) false color cross-sectional view of the spin valve with the architecture indium tin oxide (ITO)/Ni/Al_2_O_3_/CMHS/Au.

The CISS effect describes a process whereby a charge current becomes spin polarized along the current direction when passing through a chiral medium (Fig. 1A). A traditional spin valve typically consists of a hard magnet and an FM that sandwiches a normal metal or insulating tunnel barrier. The efficacy of the spin injection and transport in such structures is assessed based on the MR which is defined as

$$\text{MR}\%=\frac{G_{P}-G_{\text{AP}}}{G_{\text{AP}}}\times 100\%$$ (1)

where $G_{P}$ is the charge conductance when the FM is aligned with the hard magnet, and $G_{\text{AP}}$ is the conductance when they are antiparallel. In a CISS-based spin valve, one of the magnetic electrodes, typically the hard magnet, and the nonmagnetic interlayer are replaced by the chiral semiconductor (Fig. 1B). The CISS layer assumes the same role as the hard magnet, i.e., it is fixed with respect to the soft FM layer. The CISS-MR can therefore be similarly defined where now $G_{P}$ is the conductance when the chirality and FM are parallel (Fig. 1C), i.e., the device is in the low resistance/high conductance state, while $G_{\text{AP}}$ is when it is in the high resistance/low conductance state (Fig. 1D). Here, we adopt the notation and definitions described by Weiss *et al.* (*16*). Commonly, in CISS measurements, the current dissymmetry factor $g_{c}$ rather than MR is assessed using a magnetic conducting atomic force microscopy (mc-AFM) measurement where a nanoscale charge current, $I_{M↓,↑}$, is measured under opposite magnetization orientation of the FM, using

$$g_{c}\%=\frac{I_{M↑}-I_{M↓}}{I_{M↑}+I_{M↓}}\times 100\%$$ (2)

This quantity represents the spin-dependent charge current polarization measured in a transport experiment (*16*). Researchers have reported a broad range of $g_{c}$ values using the mc-AFM technique for CMHS films, and they are typically in the range of 50 to 94%, indicating an efficient spin polarization that should also correspond to a large MR, since the MR and $g_{c}$ are related by

$$\text{MR}=2|g_{c}|/\left(1-|g_{c}|\right)$$ (3)

However, despite the strong spin polarization inferred from these mc-AFM measurements achieving apparent MR values in the hundreds of percent, MR values in reported macroscopic CISS-based spin valves remain low (*17*–*20*). This discrepancy highlights unresolved design rules for translating the high spin polarization observed in typical CISS demonstrations to the practical implementations of CISS spin valves.

Previous reports of macroscopic CMHS spin valves have used buffer layers between the CMHS layer and a nonmagnetic top metal electrode to avoid unintended surface reaction and potential short circuits (*21*, *22*). There are also reports where no such buffer layers are used (*19*). Most demonstrated CISS-based spin valves do not have such buffer layers (*23*). This calls into question what is the best architecture for CMHS-based spin valves to pursue high MR response in practical CISS applications. To resolve this important question, we investigated three distinct spin valve architectures by varying the number of buffer layers, i.e., spin valves with two, one, and no buffer layers between the CMHS and nonmagnetic electrode. Figure S1 shows schematics of these architectures and that the highest MR value is observed for spin valves with no buffer layers. This result indicates that reducing the number of interfaces suppresses spin trapping and scattering. A typical spin valve architecture (FM/Al_2_O_3_/CMHS/Au) is shown in Fig. 1B, here an ultrathin (2 nm) Al_2_O_3_ tunneling barrier is used between the FM and CMHS layer. Au is used as the top electrode since its work function is closer to the valence band of the CMHS. Upon the application of an out-of-plane magnetic field, the spin valve exhibits either a low or high resistance state (Fig. 1, C and D) depending on the CMHS chirality and magnetization. Figure 1 (E and F) presents the scanning electron microscopy (SEM) images of the top view of the macroscopic spin valve and the cross-sectional view of the full stack. We discuss later what happens in the absence of the tunneling barrier where a smaller MR but larger current can be observed.

**Fig. 2.**
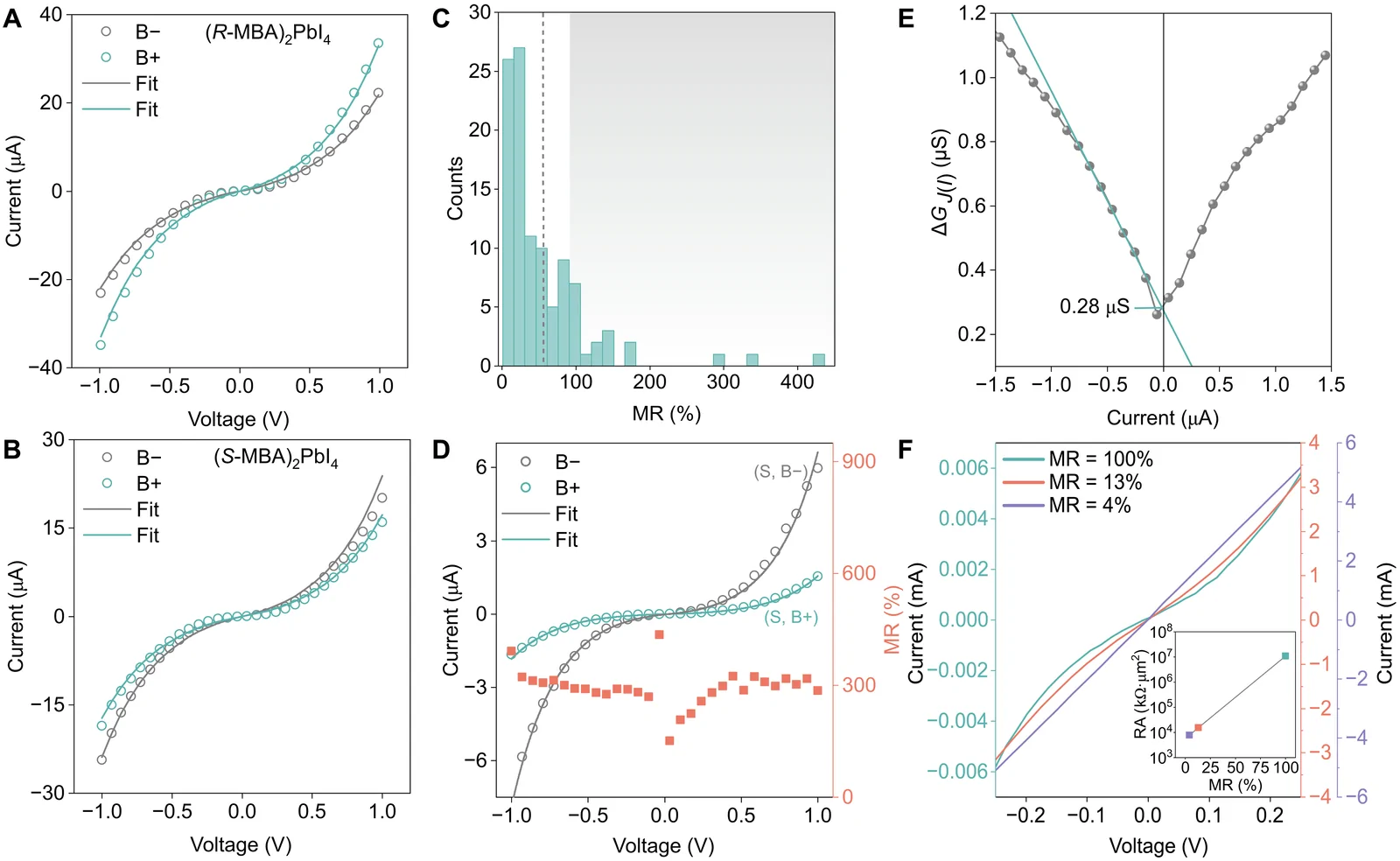
CMHS spin valves and conductance. Representative *I-V* characteristics for (**A**) (*R*-MBA)_2_PbI_4_ and (**B**) (*S*-MBA)_2_PbI_4_ spin valves. Smooth lines are the best-fitted model discussed in the text (Eq. 11) to the data with only one adjustable parameter for both curves. Measurements were performed at magnetic field values of B± = ±1 T. (**C**) Histogram of 106 spin valves for different CMHS compositions. Dotted line in (C) is indicative of the average MR value. The shaded region highlights the MR values of 100% and above. The curves in (A) and (B) are representative of the most common occurrence. (**D**) *I-V* characteristics of 298% (*S*-MBA)_2_PbI_4_ spin valve from the histogram in (C). (**E**) $∆G_{J}$ determined from the data in (D) as a function of current showing a linear dependence at low currents (green line is the best-fitted linear line) with a nonzero offset. (**F**) Comparison of *I-V*s under magnetic field (B+ = +1 T) for spin valve [FM/Al_2_O_3_/(*R*-MBA)_2_SnI_4_/Au] with different strength of tunnel junction. Inset shows the RA product versus MR, where MR decreases for leaky spin valves.

With this structure (FM/Al_2_O_3_/CMHS/Au) held constant, we first studied spin valves using the canonical (*R/S*-MBA)_2_PbI_4_. Typical room temperature current-voltage (*I-V*) characteristics (Fig. 2) of the spin valve show a nonlinear response, indicating the formation of a tunnel junction (*24*). For the (*R*-MBA)_2_PbI_4_ spin valve (Fig. 2A), a lower resistance/higher conductance is observed for the positive field, +B, compared to that for the negative field, −B, exhibiting a preferential transport of carriers with spin orientation parallel to the positive magnetic field. The *I-V* characteristics exhibit an opposite behavior for the (*S*-MBA)_2_PbI_4_ spin valve (Fig. 2B). There is a lower resistance/higher conductance for −B compared to +B (also see fig. S2). Such opposite but symmetric spin-dependent *I-V* characteristics from (*R*-MBA)_2_PbI_4_ to (*S*-MBA)_2_PbI_4_ coincides with that expected from CISS-MR phenomena (*7*). Reversing either the structural chirality or the external magnetic field orientation switches the conductance state from high to low or vice versa, while reversing both the chirality and magnetic field yields similar *I-V* characteristics. A histogram of 106 tested spin valves for various CMHS compositions (Fig. 2C and figs. S3 and S4) show that most of the fabricated spin valves in this study exhibited an MR response in the range of 20 to 50% (Fig. 2A is a representative 50% MR, and Fig. 2B exhibits MR of 20%) with an average response already showing a large improvement over literature results and more in-line with the mc-AFM results. Detailed fabrication guidelines and tunnel barrier optimization for achieving reliable spin valves are discussed in the Supplementary Materials (fig. S5). Many spin valves achieved an MR response of >100%; and in Fig. 2D, we show the performance characteristics for the spin valve with an MR of 298%. We calculated the MR from Eq. 1 (Fig. 2D, red dots) and observed very little voltage dependence. The deviation observed with champion MR values well above the typical 20 to 50% is likely due to immaculate surface chemistries with minimal interfacial reactions in those select spin valves (as discussed later). Figure S6 shows the *I-V* characteristics of other high MR spin valves.

Because the MR is measured on a macroscopic spin valve, we can also analyze the current-dependent junction magnetoconductance expressed as follows

$$∆G_{J\left(I\right)}=I\left(1/V_{M↑}-1/V_{M↓}\right)$$ (4)

where $V_{M↑}$ and $V_{M↓}$ are the voltages for B+ and B− that give the same current (*25*). Three notable features stand out in $∆G_{J\left(I\right)}$ versus current plot (Fig. 2E): (i) At low currents, the dependence is linear, while at higher currents, there is a slight deviation from linearity (green line, Fig. 2E is a linear fit to the negative current data). The linear response regime at low currents is consistent with other measurements of chiral MR and demonstrates that these spin valves exhibit a linear dependence at low bias in a two-terminal device which is characteristic of CISS enabled spin valves (see fig. S7 for analysis on other spin valves), corroborating the collinear charge-to-spin generation process through the (*S*-MBA)_2_PbI_4_ layer (*25*). The small nonlinearity at higher currents suggests that the tunneling barrier becomes current (or bias) dependent. The reduction at higher currents may also be explained by the magnon excitation mechanism (*26*), i.e., injected hot electrons at a higher voltage excite magnons and randomize the majority and minority states leading to a decrease of the spin polarization at the surface of the FM electrode. (ii) The $∆G_{J\left(I\right)}$ response in the negative and positive current regime is symmetric. Figure S8 shows $∆G_{J\left(I\right)}$ when plotted as a function of the absolute current, the values for positive currents and negative currents overlap, indicating that the electrical magnetochiral anisotropy (*27*) does not play a dominant role. (iii) The plot does not go to zero $\Delta G_{J\left(I\right)}$ at zero current. The best fit of $∆G_{J\left(I\right)}$ to a straight line (Fig. 2E, green trace) gives an offset of 0.28 μS. These features suggest that the underlying CISS mechanism is apparently odd with respect to time-reversal symmetry, as previously discussed in the literature (*13*, *28*, *29*). However, the symmetry of the *I-V* curves indicates that the mechanism is independent of the current direction.

The interface between FM and metals or semiconductors plays a critical role in determining the spin current injection efficiencies and resulting MR responses (*30*). The MR values in the present work are substantially higher than previous reports but consistent with high $g_{c}$ typically measured by mc-AFM (*2*, *31*). Since mc-AFM is performed at room temperature, even larger MR values are expected in optimized spin valve architectures, where spin transport occurs across well-defined tunnel barriers. To elucidate the origin of the high MR, three spin valves with identical composition and architecture but differing MR magnitudes were analyzed. The variation in MR correlates with the effective tunneling behavior (nonlinearity in *I-V*) of the junction, exhibiting a clear decrease in MR as the junction becomes more conductive (*32*–*34*). Spin valves with leaky barriers show markedly reduced MR (Fig. 2F and fig. S9), underscoring the decisive role of tunnel barrier in governing spin transport. This can be better visualized as the resistance-area (RA) product defined as the product of the device resistance and its active junction area. In spin valves where a tunnel barrier is present, the effective barrier thickness and quality directly determine the RA value (*35*–*37*). Here, we observed an increasing MR (Fig. 2F, inset) with increasing RA, a feature observed in traditional tunnel junctions (*38*, *39*). The discussion on the intricate balance between RA and MR is beyond the scope of the present work; however, it should be noted that high RA has been previously reported for spin valves using insulating tunnel barriers (*40*–*46*). RA for leaky spin valves is orders of magnitude lower, and the corresponding MR approaches the low values generally observed in CMHS-based spin valves (fig. S10). Imperfections such as pinholes or incomplete barrier formation can facilitate spin-independent charge leakage, thereby suppressing spin selectivity and reducing the overall MR response (*47*). These observations emphasize that achieving high-quality, well-defined interfaces is essential for realizing high CISS-MR.

Another related interfacial effect that can also have strong implications in the context of the chiral semiconductors and the spin valves studied here involves interfacial chiral magnetic proximity effects. It has been shown that the adsorption of chiral molecules on top of FMs can alter the remanent magnetization of the FM layer (*48*). In addition, there is enantiospecific adsorption of chiral molecules on the FM surfaces depending on the magnetization direction of the FM (*49*). These phenomena suggest that a strong chiral-modulated spin-exchange interactions occur between FM contacts and adjacent chiral molecules, although no electron flow is present at the interface. This effect has been termed magnetism induced by proximity of adsorbed chiral molecules (MIPAC) (*48*). Although CMHSs are substantially different from chiral molecules, we need to examine whether the MIPAC effect is also present at the FM/CMHS interface and if it plays a role in the observed MR responses. Thus, we fabricated macroscopic (*R/S*-MBA)_2_PbI_4_ spin valves without an Al_2_O_3_ tunneling barrier which allows for a direct contact between the FM and CMHS layer. Without the tunneling barrier, an ohmic *I-V* characteristic with high current levels is observed due to electrical short circuits between the top Au contact and the bottom Ni contact. Although the current does not traverse the CMHS layer, we still observed an MR response, i.e., with the high conductance state for one enantiomer corresponding to a given magnetic field direction and for the opposite enantiomer corresponding to the reversed magnetic field direction (Supplementary Text and figs. S11 to S14). These results indicate a proximity effect between the CMHS and FM upon contact, even in the absence of direct current flow through the CMHS. This behavior was also validated by using axially chiral phosphate molecules, and we observed that the MIPAC effect vanishes upon introducing a polymer layer between the FM and chiral layers (fig. S15). We propose that this interaction arises from CISS but manifested through the MIPAC effect. The MR response from the electrically shorted structures is smaller compared to the MR measured in the spin valves with a tunnel barrier. The presence of the MIPAC effect indicates that there is a chirality-induced spin-exchange interaction between CMHS and FM similar to chiral organic molecules.

### CISS enabled modulation of tunneling barrier

The high values of MR and $g_{c}$ in the present case are in apparent contradiction with the Julliere model of MR (*50*), which should limit $g_{c}$ to be the product of the intrinsic spin polarization of the FM, ${\text{SP}}_{\text{FM}}$, and that of the CISS layer ${\text{SP}}_{\text{CISS}}$, i.e.

$$g_{c}={\text{SP}}_{\text{FM}}\cdot {\text{SP}}_{\text{CISS}}$$ (5)

Here, the intrinsic spin polarization is defined by Eq. 6

$${\text{SP}}_{i}\%=\frac{n_{i,↑}-n_{i,↓}}{n_{i,↑}+n_{i,↓}}\times 100\%$$ (6)

where $n_{i,↑},n_{i,↓}$ denote the density of states for spin-up and spin-down carriers *n* at the Fermi level in each material i. As a result, in the Julliere model, the current dissymmetry factor, $g_{c}$, should not be greater than the spin polarization of the FM, in our case, Ni with ${\text{SP}}_{\text{FM}}$= 33% at room temperature (*51*). Similarly, the MR is limited as follows

$$\text{MR}=2\left({\text{SP}}_{\text{FM}}\cdot {\text{SP}}_{\text{CISS}}\right)/\left(1-{\text{SP}}_{\text{FM}}\cdot {\text{SP}}_{\text{CISS}}\right)$$ (7)

and thus should be limited to ∼98.5% for Ni in the Julliere model assuming the maximum value ${\text{SP}}_{\text{CISS}}=100\%$. However, the observed MR and $g_{c}$ greatly exceeded these limits in our experiments which suggest that ${\text{SP}}_{\text{CISS}}$ would be greater than 100%, which is not physical. This apparent paradox arises from oversimplifications in the Julliere model, which does not account for a spin-dependent tunneling barrier (*52*–*54*). MacDonald and coworkers (*55*) generalized the Julliere model to account for such a spin-dependent tunneling barrier. In the MacDonald model, the intrinsic spin polarizations ${\text{SP}}_{i}$ of each electrode (i= 1 or 2) in a spin-dependent tunnel junction should be each replaced by an effective spin polarization ${\text{SP}}_{i}^{\text{eff}}$ which can be written as

$${\text{SP}}_{i}^{\text{eff}}=\frac{f_{i}\;\left(\tau_{i}+1\right)-1}{f_{i}\;\left(\tau_{i}-1\right)+1}$$ (8)

In this model, the MR is given by

$$\text{MR}=\frac{2\;{\text{SP}}_{\text{FM}}^{\text{eff}}{\text{SP}}_{\text{CISS}}^{\text{eff}}}{\left(1-{\text{SP}}_{\text{FM}}^{\text{eff}}{\text{SP}}_{\text{CISS}}^{\text{eff}}\right)}\times 100\%$$ (9)

Here, the parameter $f_{i}=\left({\mathrm{SP}}_{i}+1\right)/2$ where ${\text{SP}}_{i}$ is the spin polarization of electrode i, while $\tau_{i}=t_{i,↑}/t_{i,↓}$ is the ratio of the transmission amplitude factors for spin-up versus spin-down carriers in each electrode i; this assumes that the barrier tunneling probability for each spin state can be factored as ${|t_{↑\left(↓\right)}|}^{2}=t_{1,↑\left(↓\right)}\;t_{2,↑\left(↓\right)}$ (Supplementary Text). Assuming a symmetric tunneling barrier, $\tau_{1}=\tau_{2}=\tau ,$ the effective polarization of the FM is given by Eq. 10

$${\text{SP}}_{\text{FM}}^{\text{eff}}=\left[f\left(\tau +1\right)-1\right]/\left[f\left(\tau -1\right)+1\right]$$ (10)

As for the CISS layer, even assuming negligible intrinsic spin polarization, i.e., ${\text{SP}}_{\text{CISS}}=0$, and $f_{\text{CISS}}$ = ½, the effective polarization of the CISS layer can be described as

$${\text{SP}}_{\text{CISS}}^{\text{eff}}=\left(\tau -1\right)/\left(\tau +1\right)$$

**Fig. 3.**
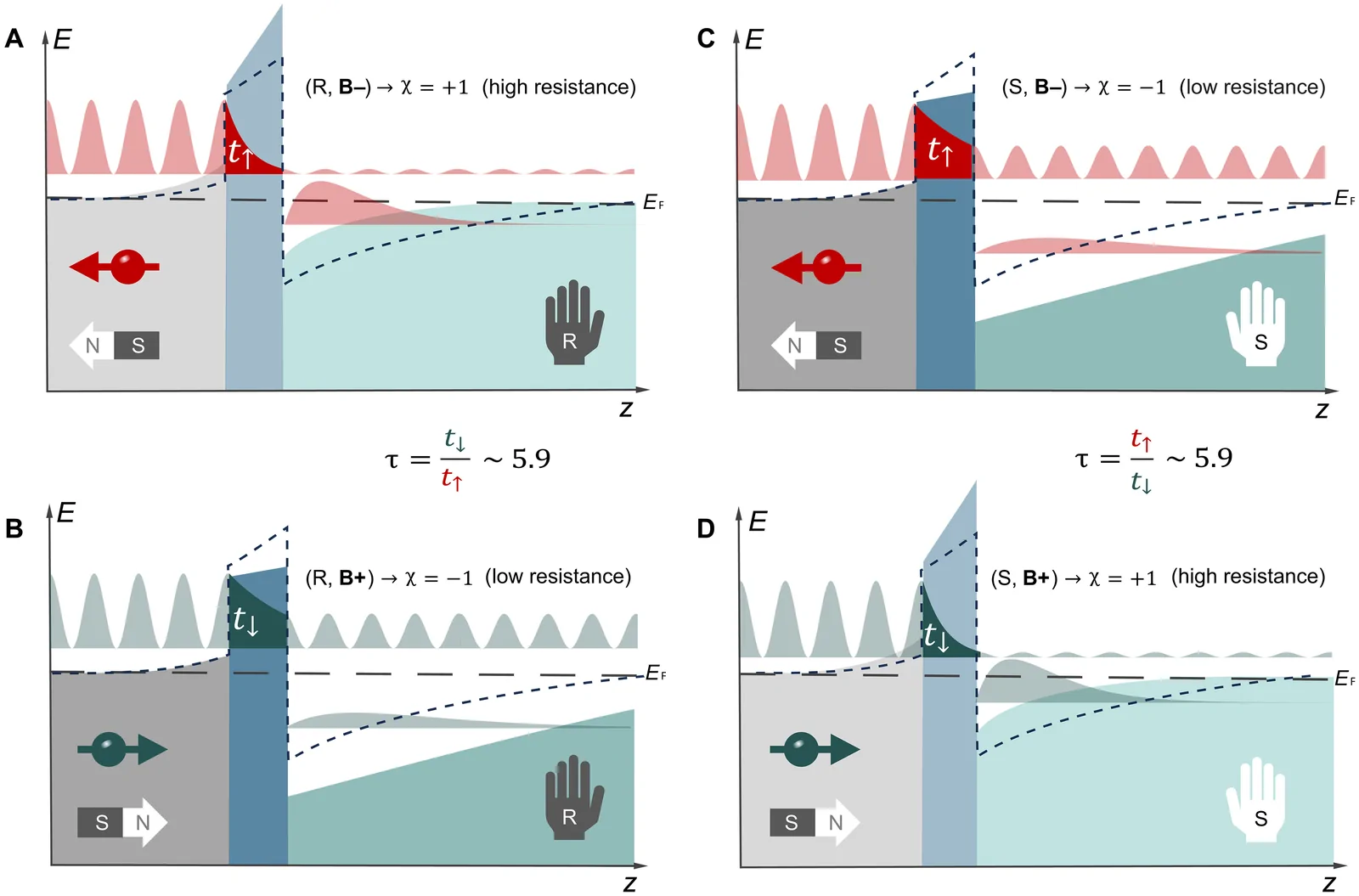
Modeling and CISS effect at the FM/CMHS interface. (**A** to **D**) Band alignment diagrams for the spin valves with tunnel barrier in the FM-dielectric-CMHS interface at zero bias voltage. The magnetic electrode is shown in gray and the tunnel barrier in blue, with the dashed line indicating the position of the tunnel barrier in an achiral state. Changes in the magnetochiral local density of states affect the electrostatic screening at the interfaces and modulate the tunnel barrier. The chiral semiconductor corresponds to the green area. The four quadrants show all configurations of a spin valve and the symmetry of *I-V* curves, under reversal of chirality and magnetic field direction, with (A) and (B) and (C) and (D) showing opposite enantiomers (black and white hands), while (A) and (C) and (B) and (D) show opposite magnetization (denoted by the arrow). The diagonals [(A) and (D)] are in a high-resistance state with the symmetric pairing of (R, −B) and (S, +B), while (B) and (C) represent the low-resistance state with symmetric pairing of (R, +B) and (S, −B). When the magnetic field direction and chirality are matched, the tunneling barrier is low, and the tunneling rate is high. The ratio of the tunneling rates, τ, between the high resistance and low resistance state governs the MR discussed in the text, and for the MR of 298%, the ratio is ∼5.9. The schematic was edited and exported using Inkscape, version 1.3.

Thus, one can easily check if τ=10 and ${\text{SP}}_{\text{FM}}=33\%$, then $g_{c}={\text{SP}}_{\text{FM}}^{\text{eff}}{\text{SP}}_{\text{CISS}}^{\text{eff}}\times 100\%=74\%$, and the corresponding MR would be 570%. For τ>10, the values of $g_{c}$ and MR could easily increase. For our observed MR value up to 298% (Fig. 2D), this model would imply that τ should be ∼5.9 (see Fig. 3 and discussion below).

To gain further insight into the chirality and spin-modulated tunneling and the calculated ratio of the transmission amplitude factors, τ for spin-up versus spin-down carriers in the CISS layer, it is useful to develop a predictive model that can account for the spin- and chirality-dependent barrier physics and the true band structure at the interfaces. The nonlinear *I-V* characteristics of the spin valves can be best described by a tunneling current through a rectangular barrier of height $E_{b}+\chi \Delta$ (Fig. 3 and Supplementary Text) where χΔ is the CISS-induced change in the work function that is due to magneto-chiral interaction

$$I∼V\text{exp}\left[-2s\;\sqrt{\frac{2m}{\hbar^{2}}\left(E_{b}+\chi \Delta -e|V|\right)}\right]$$ (11)

with the applied bias V, the width of the tunneling barrier s=2 nm, and the mass m and charge of an electron e. Modeling the *I-V* characteristics in the achiral state where ∆=0 yields an Al_2_O_3_ barrier height of $E_{b}$ = 2.9 to 3.3 eV consistent with literature (*56*). Thus, for the CMHS spin valves, ∆ is the only adjustable parameter to describe both $I_{M↑}$ and $I_{M↓}$, and χ captures the symmetry of the *I-V* curves under reversal of chirality/magnetic field, where χ=+1 represents the high resistance state and corresponds to the enantiomer and magnetization pairing (R, −B) and (S, +B) (Fig. 3, A and D), while χ=−1 is the low resistance state corresponding to the opposite pairings (R, +B) and (S, −B) (Fig. 3, B and C). The experimental *I-V* characteristics are well reproduced by Eq. 11 consistent with our hypothesis [smooth lines in Fig. 2 (A, B, and D)]. Here, we simultaneously model the +B and −B data to obtain the best-fitted value of ∆. The CISS-induced change in work function found here varies between Δ = 20 and 100 mV depending on the exact CMHS. These values are of the same order of magnitude as found in specific measurements of the work function of peptide layers indicating similarities between CMHS and chiral molecules (*57*–*59*) and results in a spin- and chirality-dependent tunneling rate proportional to ${|t_{↑\left(↓\right)}|}^{2}$ as described above.

Two broad classes of interpretation have been proposed for the MR observed in chiral/FM systems. The first class invokes spin-dependent transport, specifically, different transmission probabilities for spin-up and spin-down carriers, in analogy with the Julliere spin valve model. The second class, advanced by Tirion and van Wees (*13*), argues that the observed MR is inconsistent with a Julliere-type spin valve picture and instead arises from an equilibrium electrostatic modification of the transport barrier due to a magnetochiral contact potential that depends on both the chirality handedness and the direction of magnetization, rather than on intrinsic spin-selective transport through the chiral layer itself. Our model builds on this electrostatic framework: We similarly attribute the MR to a chirality- and magnetization-dependent modification of the tunneling barrier height. The present work extends this picture by connecting the barrier modification to a specific spin-charge coupling mechanism (*29*, *60*), where spin-selective charge localization modifies the local density of states at the chiral/FM interface, producing a chirality- and magnetization-dependent electrostatic screening that results in an effective spin-dependent tunneling rate.

It has been experimentally shown that CISS modulations of the work function, decay distance, and quantum capacitance are observable consequences of the same effect without the need of charge transport across the chiral interface (*59*). The square root in Eq. 11 can be interpreted as a chirality and spin-dependent decay length expressed as

$$1/\lambda =\sqrt{\frac{2m}{\hbar^{2}}\left(E_{b}+\text{χΔ}-e|V|\right)}$$ (12)

or equivalently by a localization shift of the spin-dependent wave function leading to a chirality-induced quantum capacitance. The effectiveness of this simple tunneling model arises from the spin valve design and operational parameters. Band edge alignment creates an ohmic contact between the top Au electrode and the CMHS, while band bending at the Al_2_O_3_/CMHS interface dominates the tunneling transport characteristics (Fig. 3, A to D). The dielectric nature of Al_2_O_3_ modulates the tunneling barrier height based on effective work function differences. Since electron kinetic energy is well below the barrier, Fowler-Nordheim tunneling does not occur, making a rectangular tunnel barrier a valid approximation. The applied potential induces field changes much smaller than those from band bending, with chirality-induced effects being more substantial. A back-of-the-envelope calculation shows a field change of 50 MV/m from chirality-induced effects, compared to less than 0.6 MV/m from a 0.1 V applied external bias (Supplementary Text). So, a small work function change can induce notable changes in the transport characteristics.

The interaction of the chiral semiconductor with magnetic layer affects the local density of states such that when the chirality and magnetic field are aligned (low resistance state; Fig. 3, B and C), there is a lower barrier leading to a tunneling rate that is higher than when they are anti-aligned (high resistance state; Fig. 3, A and D). The microscopic origin of the work function modulation, captured in the model via the phenomenological energy Δ, has recently been connected to a spin-charge coupling mechanism in chiral systems (*60*, *61*), although a complete quantitative description of its dependence on chirality and magnetization remains an active area of investigation. Consequently, the model reproduces the chirality/magnetic field symmetry observed in the *I-V* characteristics and explains the deviation from the Julliere model due to the spin-dependent chirality-induced tunneling rate at the interface. The barrier modulation picture presented here is a physically motivated proposition, supported by the consistency of our transport data with the model and by quantitative agreement with work function shifts reported in analogous chiral systems. Notably, conductance differs at zero bias between opposite enantiomers under the same magnetic field polarity or the same enantiomers under opposite polarities, consistent with a behavior that is apparently odd with respect to time-reversal symmetry at equilibrium, independent of transport, and motivates further theoretical development.

## DISCUSSION

### CISS-MR dependence on composition and symmetry

**Fig. 4.**
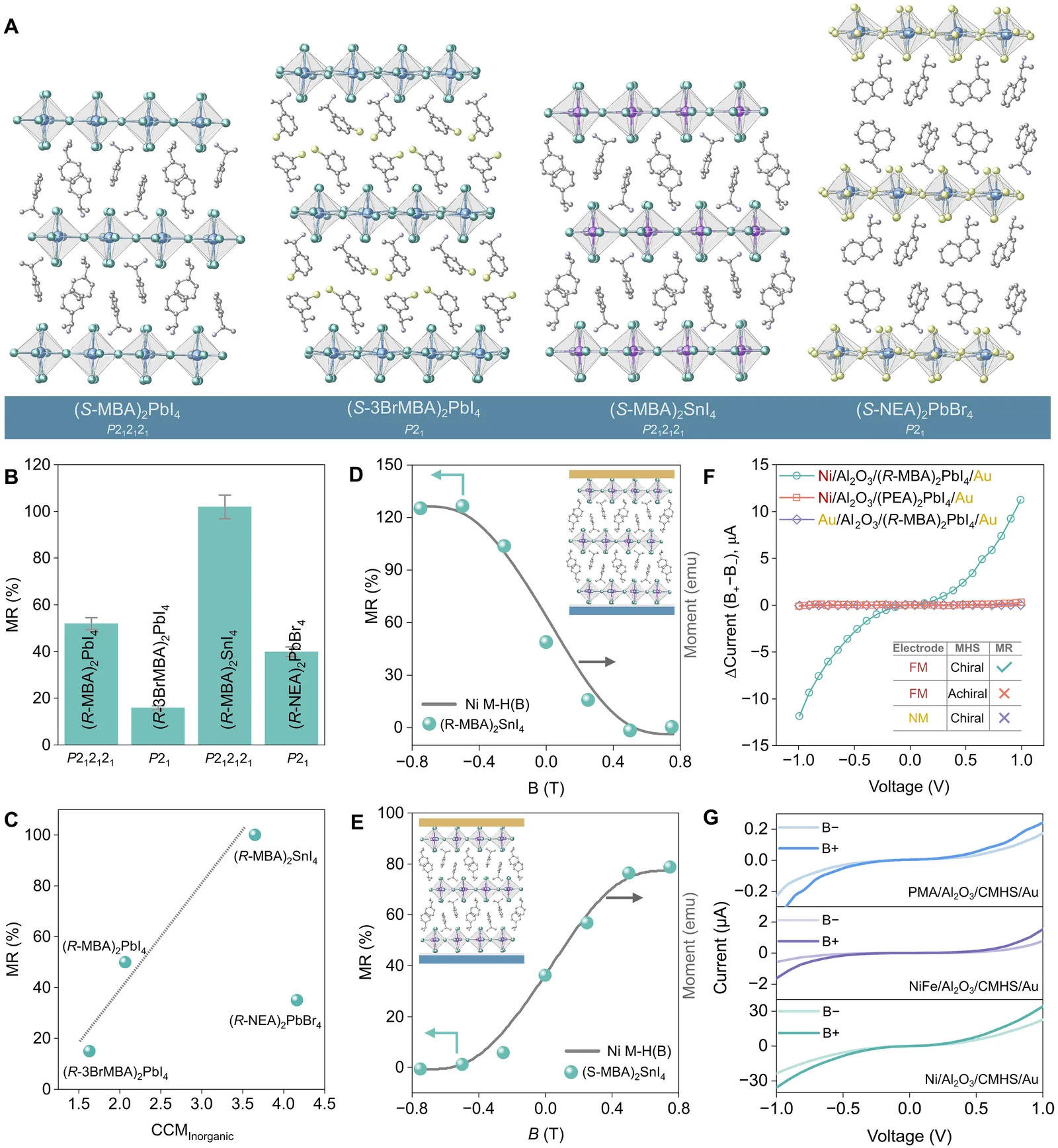
MR dependence on CMHS composition. (**A**) Crystal structure of (*S*-MBA)_2_PbI_4_, (*S*-3BrMBA)_2_PbI_4_, (*S*-MBA)_2_SnI_4_, and (*S*-NEA)_2_PbBr_4_. MBA-based CMHS have *P*2_1_2_1_2_1_ symmetry, while 3BrMBA- and NEA-based CMHS have *P*2_1_ symmetry. (**B**) Representative room temperature MR values for various CMHS calculated from the current-voltage characteristics under out of plane magnetic field (B± = ±1 T). (**C**) MR values as a function of inorganic CCM. The gray dotted line is a guide to the eye showing correlation between CCM of inorganic lattice with MR. Field-dependent MR(B) response for (**D**) (*R*-MBA)_2_SnI_4_ and (**E**) (*S*-MBA)_2_SnI_4_ spin valve at an applied bias of −0.25 V. Green points are measured MR values by sweeping the field from positive to negative or vice versa. Gray lines show the magnetic hysteresis M-H(B) loop of the Ni layer measured by a superconducting quantum interference device, which overlaps the MR(B) data, confirming the CISS-MR response in the current spin valve configuration. (**F**) The difference in the current under B+ and B− magnetic field of ±1 T extracted from *I-V* measurements under different electrode and MHS configurations. MR response is only observed when both FM and CMHS are present. FM and NM denote ferromagnetic and nonmagnetic contacts, respectively. (**G**) Room temperature *I-V* characteristics (B± = ±1 T) for (*R*-MBA)_2_PbI_4_ spin valves with Ni, NiFe, and PMA (Pt/Co superlattices) FM layers. Fabrication details were similar for all spin valves. Ni and NiFe thicknesses were 30 nm, and PMA layer consisted of six alternating thin layers of Co and Pt. emu, electromagnetic unit.

Compared to traditional chiral systems, the synthetically tunable composition and high crystallinity of CMHS thin films allow both tailoring and quantification of the structural symmetry breaking in addition to the metal-halide composition, thus enabling correlation of the symmetry breaking to the resultant MR characteristics. For instance, the choice of chiral organic cation in the CMHS determines its space groups with distinct mirror symmetry breaking. The two most common space groups for CMHS are the *P*2_1_ and *P*2_1_2_1_2_1_ space groups (*31*). The *P*2_1_ space group has an in-plane 2_1_ screw axis although the chiral organic cation’s chiral axis is mainly oriented out of plane, while the *P*2_1_2_1_2_1_ structures have 2_1_ axes along each crystallographic direction. Note that the 2_1_ screw axis symmetry operation by itself is not chiral; however, when chiral molecules are present in the structure, they also lack mirror symmetry, in that case, the 2_1_ screw axis is also a chiral axis (*62*). To elucidate this structure-property correlation, we selected four types of CMHS compositions with *P*2_1_2_1_2_1_ and *P*2_1_ space groups (Fig. 4A) by varying A-, B-, and X-sites (*63*, *64*), i.e., (*R/S*-MBA)_2_PbI_4_, (*R/S*-3BrMBA)_2_PbI_4_, (*R/S*-MBA)_2_SnI_4_, and (*R/S*-NEA)_2_PbBr_4_. We performed spin-dependent *I-V* characteristic measurements using the same architecture FM/Al_2_O_3_/CMHS/Au. Pure phase of CMHS thin films was obtained by dissolving their respective single crystals and spin casting on the substrate (fig. S16). CMHS layer exhibits smooth and flat microstructure on the FM/Al_2_O_3_ substrate (fig. S17). Figure 4B represents the MR calculated from *I-V* characteristics (fig. S18) of the macroscopic spin valves upon both positive and negative magnetic fields of 1 T. Note that these values represent the most common MR results for each CMHS composition.

We found that the CISS-MR follows the trend (*R*-MBA)_2_SnI_4_ > (*R*-MBA)_2_PbI_4_ > (*R*-NEA)_2_PbBr_4_ > (*R*-3BrMBA)_2_PbI_4_. We find the CISS-MR is larger for the *P*2_1_2_1_2_1_ space group, where the 2_1_ screw axis aligns with the charge current direction; however, a substantial MR can be observed in the *P*2_1_ space group despite the absence of collinear 2_1_ screw axis. Recent analysis of the spin textures for the *P*2_1_ space group found symmetry-allowed chiral spin-splitting terms which produce spin polarization parallel to the carrier wave vector along both the out-of-plane and in-plane directions consistent with density functional theory simulations irrespective of whether a given direction coincides with the 2_1_ screw axis (*65*). Their work highlighted that these chiral spin-splitting terms arise due to the absence of any mirror symmetries rather than the presence of a specific screw axis, suggesting that neither the crystal space group nor the screw axis direction serves as a reliable measure per se to correlate with the CISS response.

To reveal the structural correlation, we compared two other metrics of chirality: circular dichroism (CD) and the CCM. The CD dissymmetry factor $g_{\text{CD}}$ is an approximate empirical metric for the “degree” of chirality (in the absence of directionally asymmetric “apparent” CD effects which are uncorrelated to intrinsic chirality and can be observed in both chiral and achiral solids). CD spectra for all CMHS films exhibit an onset consistent with their optical absorption spectra (figs. S19 and S20), indicating successful chirality transfer to the inorganic framework (*65*). We find that the $g_{\text{CD}}$ shows a trend when comparing CMHS within the same space group: (*R*-MBA)_2_SnI_4_ > (*R*-MBA)_2_PbI_4_ and (*R*-3BrMBA)_2_PbI_4_ > (*R*-NEA)_2_PbBr_4_ (fig. S21). The higher $g_{\text{CD}}$ in the case of (*R*-MBA)_2_SnI_4_ has been attributed to higher octahedral bond distortion, compared to that in Pb, leading to higher chiroptical activity (*64*, *66*, *67*). The trend of $g_{\text{CD}}$ and MR is consistent for (*R*-MBA)_2_SnI_4_ and (*R*-MBA)_2_PbI_4_ (fig. S22).

On the other hand, CCM provides a quantitative descriptor for the degree of chirality (distortion) by calculating how far away the chiral structure is from the nearest achiral structure (*68*–*70*). We used the racemic structure of each composition as a reference to calculate the CCM (Supplementary Text and fig. S23). We can also easily separate out the total CCM into the organic and the inorganic contribution. They do not correlate with one another. Although the organic component dominates the total CCM, the structures with larger organic CCM do not correspond to a larger inorganic CCM (fig. S24). The MR scales with the inorganic CCM for MBA-based CMHS (straight line in Fig. 4C and fig. S25). However, while (*R*-NEA)_2_PbBr_4_ exhibits the largest CCM, it exhibits a smaller MR and thus does not follow the correlation. This could arise due to the difference in mass of I compared to Br and any resulting effect on spin-orbit coupling and CISS or due to changes in the energy levels (valence band/conduction band) relative to Ni. We find that the $g_{\text{CD}}$ only correlates to CCM for (*R*-MBA)_2_SnI_4_ and (*R*-MBA)_2_PbI_4_ (fig. S26). To summarize the structure-property correlations, (i) the space group observation (presence of MR for both *P*2_1_2_1_2_1_ and *P*2_1_) is consistent with previous theoretical work (*65*), suggesting that chiral spin splitting requires the absence of mirror symmetry rather than any specific screw axis alignment. (ii) Across all four compositions, no single simple structural metric (space group, *g*_CD_, and CCM) provides a universal, quantitative predictor of MR, likely because MR in these devices is a multiparameter quantity that depends on both the intrinsic CISS activity of the active layer and device-level factors such as interface quality, film morphology, and tunneling barrier characteristics. (iii) Within the present dataset, the inorganic CCM is a better predictor of MR than the organic CCM or total CCM within the MBA cation series. This suggests that chirality in the inorganic sublattice (the component directly involved in charge transport) is the more relevant descriptor.

To confirm that the spin valve response follows the typical magnetic polarization of the FM contact (fig. S27), field-dependent MR was measured. Field-dependent resistance measurements on (*R/S*-MBA)_2_SnI_4_ spin valves exhibit opposite MR response depending on the chirality of the CMHS layer and applied field (Fig. 4, D and E, and fig. S28). Further discussion on field dependence of MR and the protocol followed are provided in the Supplementary Materials (fig. S29). To further validate that the resistance difference in the *I-V* characteristics truly originates from the CISS effect and simply not due to the presence of a magnetic field (*71*), we fabricated control samples by replacing the FM contact with a nonmagnetic electrode and CMHS with achiral MHS (PEA)_2_PbI_4_. The *I-V* response of the spin valves with the architecture Au/Al_2_O_3_/(*R*-MBA)_2_PbI_4_/Au and Ni/Al_2_O_3_/(PEA)_2_PbI_4_/Au shows a negligible resistance difference under positive and negative fields (Fig. 4F and figs. S30 and S31), confirming that the FM contact and CMHS are essential to detect the CISS-MR response. To further demonstrate the impact of the magnetic field on the spin valve resistance, we measured in situ continuous current response while reversing the magnetic field direction from −B to +B over several cycles (fig. S32). The current-time (*I-t*) response curve shows an abrupt change upon switching the field orientation, confirming the spin character of the CISS effect for the CMHS-based spin valve while the achiral MHS spin valve has no response to the magnetic field. To confirm the high MR observed is not specific to Ni, two other FM layers, NiFe and PMA (Co/Pt), were also used in spin valves. NiFe and PMA-based spin valves show clear distinction between current values in opposite fields (Fig. 4G) confirming the CISS-MR in the spin valves. The lower current level in the case of NiFe and PMA can be rationalized as the difference in the interfacial energetics and quality (*72*).

The combination of macroscopic spin valve architecture exploration and measurements of multiple chiral halide perovskite compositions in this work enabled MR values to reach >100% in CISS-based spin valves. The correlation of MR with the inorganic CCM and the orientation of chiral screw axes suggest that the CISS behaviors in chiral halide perovskites can be tailored and offer great potential for high-throughput compositional screening. Furthermore, we revealed the presence of two types of spin-dependent interfacial effects related to CISS: TMR and MIPAC based on the interfacial layer composition. We identified the important role of the spin-dependent tunneling barrier enabled by CISS in chiral spin valves which will provide device design rules in controlling spin at room temperature using solution-processed hybrid semiconductors. The molecular flexibility and tunable symmetry breaking of halide perovskites offer a compelling platform for the advancement of exploiting spin, charge, and light.

## MATERIALS AND METHODS

### Chemicals and materials

R/S-α-methylbenzylamine (R/S-MBA), R/S-(1-naphthyl)ethylamine (R/S-NEA), 2-Phenethylamine (PEA), lead(II) iodide (PbI_2_), lead(II) bromide (PbBr_2_), tin(IV) oxide (SnO_2_), hypophosphorous acid (H_3_PO_2_), hydrobromic acid (HBr), diethyl ether, bathocuproine (BCP), *N*,*N*-dimethylformamide (DMF), and (R)-1,1′-binaphthyl-2,2′-diyl hydrogenphosphate (R-BHP) were purchased from Sigma-Aldrich. Hydroiodic acid (HI) was purchased from Thermo Fisher Scientific. (R/S)-1-(3-bromophenyl)ethanamine (R/S-3BrMBA) was purchased from Synthonix. PEDOT:PSS (Clevios AI 4083) was from Heraeus. All reagents were used without further purification.

### Single crystal growth

(R/S-MBA)_2_PbI_4_: PbI_2_ (413 mg) was dissolved in 5.5 ml of concentrated HI acid and 0.5 ml of concentrated H_3_PO_2_ acid. The solution was then cooled over ice, and 228 μl of R/S-MBA was added dropwise. The mixture was then heated at 120°C until it was fully dissolved. The solution was then placed in a 75°C aluminum heating block and cooled to room temperature at a rate of 1°C/hour. Orange needle-like crystals were collected through vacuum filtration, and the crystals were washed with 20 ml of diethyl ether three times followed by drying in a vacuum oven at 55°C, 150 torr.

(R/S-NEA)_2_PbBr_4_: PbBr_2_ (90.0 mg) was dissolved in 1.0 ml of concentrated HBr acid by heating at 85°C. After cooling the solution to room temperature, 2.4 ml of DI water was slowly added to the mixture. Subsequently, the solution was cooled to 0°C, and R/S-NEA (87 μl) was added dropwise. This mixture was then heated at 95°C while stirring for a minimum of 2 hours to ensure complete dissolution of precipitates. The solution was then left to cool to room temperature at a rate of 2°C/hour, and crystals were collected over vacuum filtration.

(R/S-MBA)_2_SnI_4_: SnO_2_ (135 mg) was completely dissolved in 5.5 ml of HI acid and 0.5 ml of concentrated H_3_PO_2_ acid by stirring and heating at 120°C overnight. The solution was cooled to room temperature. R/S-MBA (228 μl) was added dropwise and a precipitate formed. The mixture was heated while stirring at 120°C (<10 min) until all solids were dissolved. The vial was subsequently transferred to an aluminum heating block (preheated at 90°C) and cooled to room temperature at a rate of 1°C/hour. In a nitrogen atmosphere, orange rod-like crystals were collected via vacuum filtration followed by washing with degassed diethyl ether (N_2_ sparged ∼10 min at 0°C). The crystals were then transferred to a vacuum oven at RT and dried overnight.

(R/S-3BrMBA)_2_PbI_4_: PbI_2_ (231 mg) was dissolved in 8.5 ml of concentrated HI acid and 0.5 ml of concentrated H_3_PO_2_ acid by heating at 100°C (<5 min). The solution was cooled to room temperature, R/S-3BrMBA (151 μl) was added dropwise to the solution, and an orange precipitate formed. The mixture was heated at 120°C while stirring until completely dissolved (∼1 hour). The solution was then placed in an aluminum heating block preheated to 70°C and cooled to room temperature at a rate of 1°C/hour. Orange crystals were collected via vacuum filtration, washed with 50 ml of diethyl ether three times, and then allowed to dry at 55°C overnight in a vacuum oven.

(PEA)_2_PbI_4_: PbI_2_ (200 mg) was first fully dissolved in 7 ml of HI and 1.5 ml of H_3_PO_2_ at 100°C. Then, PEA (200 μl) was added and kept at 100°C for 1 hour. The solution was then slowly cooled to room temperature at a rate of 2°C/hour. The crystals were then isolated from the parent solution by filtration and washed with diethyl ether. Last, the crystals were dried at 70°C for 5 hours.

### Spin valve fabrication

The FM layer (30 nm) of Ni or NiFe was deposited on cleaned indium tin oxide by e-beam evaporation. Spin valves with PMA layer were fabricated by depositing Co and Pt via radio frequency sputtering, and the process was repeated six times to build up the heterostructure. A 2-nm tunneling layer of Al_2_O_3_ was deposited on the top of the FM by atomic layer deposition. Atomic layer deposition was performed with pulsed trimethylaluminum and water at 200°C to form 2-nm Al_2_O_3_. Before CMHS film deposition, the substrates were subjected to ozone for 1 min. For CMHS thin films, single crystals were dissolved in DMF with a concentration of 200 mg/ml. CMHS films were prepared by spin coating 50 μl precursor solution on the substrate at 4000 rpm for 30 s, followed by annealing at 100°C for 10 min in a glove box. Depending on the device architecture, BCP(15 nm)/MoO*_x_*(15 nm)/Au(80 nm) or MoO*_x_*(15 nm)/Au(80 nm) or only Au(80 nm) was deposited by thermal evaporation on the top of the CMHS layer to complete the spin valve fabrication. Devices with PEDOT:PSS and R-BHP were fabricated by spin coating PEDOT:PSS on Ni at 5000 rpm. R-BHP was dissolved in DMF (10 mg/ml) and spin coated at 5000 rpm on bare Ni or PEDOT:PSS-coated Ni.

### Spin valve characterization

Electrical characterization (*I*-*V* characteristics) of the freshly fabricated spin valves was carried out using a custom-built sample holder connected to a Keithley 2400, controlled by a MATLAB code. Each substrate/sample consisted of six spin valves that were measured in sequence. The current/voltage compliance level for each CMHS composition was determined by trial until the device failed under test conditions. The *I*-*V* characteristics were collected by placing the sample holder under a fixed magnetic field of ±1 T at room temperature using Lakeshore narrow gap fixed magnet. Magnetic field–dependent measurements for spin valves with tunneling layers were measured in air using an electromagnet from GMW Associates. For spin valves without a tunneling layer, magnetic field–dependent measurements were performed in a Physical Property Measurement System (Quantum Design lnc.). Magnetoconductance measurements were carried out by applying a dc bias to the sample and measuring the resulting current. All measurements were performed at room temperature and in dark unless otherwise stated.

### Material characterization

XRD measurements were performed using Rigaku Ultima IV with Cu Kα radiation at ambient temperature. SEM images were taken on a Hitachi S-4800 scanning electron microscope. UV-Vis absorption spectra were taken on a Cary 7000 spectrometer. CD measurements were carried out using an Olis DSM 170 spectropolarimeter. An ION-TOF TOF-SIMS V spectrometer was used for depth profiling of MHS films. Magnetic hysteresis measurements for Ni were performed using a Quantum Design Magnetic Property Measurement System equipped with a superconducting quantum interference device magnetometer. The sample was mounted in a nonmagnetic sample holder and centered in the detection coils to ensure accurate measurement. Magnetic moment was recorded as a function of applied magnetic field at a fixed temperature of 300 K, with the field swept between ±1 T. Data were corrected for the diamagnetic background of the sample holder.

## Supplementary Materials

### This PDF file includes:

Supplementary Text Figs. S1 to S32 References
