# Supplementary material for: Chirality-induced magnetoresistance in hybrid organic-inorganic perovskite semiconductors
Source: Sci Adv. 2026 Sep 9;12(37):eaed7176. doi: 10.1126/sciadv.aed7176 (PMC13557078; doi:10.1126/sciadv.aed7176)
Supplement: Supplementary file 1 [file sciadv.aed7176_sm.pdf]

Science Advances
AAAS

Supplementary Materials for

Chirality-induced magnetoresistance in hybrid organic-inorganic perovskite semiconductors

Md Azimul Haque *et al.*

Corresponding author: Matthew C. Beard, matt.beard@nlr.gov

Sci. Adv. **12**, eaed7176 (2026)
DOI: 10.1126/sciadv.aed7176

This PDF file includes:

Supplementary Text
Figs. S1 to S32
References

Spin valve fabrication guidelines

To ensure high quality spin valves, we follow a specific fabrication protocol. Figure S5A depicts the fabrication steps. ITO coated glass substrates were used to ensure good bottom electrode contacts. The substrates were masked to deposit ferromagnetic layers on the ITO. Afterwards the substrates were masked again to deposit the tunneling layer such that Al_2O_3 covers the edges of ferromagnetic layer to prevent electrical shorting with the top metal electrode. CMHS layer was coated on the tunneling layer and then CMHS layer was scratched off from the contact areas. Finally, top metal electrode was deposited through a shadow mask using thermal evaporation. For electrical characterization, the compatible voltage range should be determined by scanning different voltage ranges (from low to high range). Starting the measurement from high voltage can break the spin valve. Spin valves with same architecture and composition can have variations in the performance. High MR spin valves have good tunnel junctions and MR decreases for leaky spin valves. The quality of CMHS layer synthesized from different batches of single crystals should be checked by CD measurements to ensure spin valve data reproducibility.

To understand the critical role of the Al_2O_3 tunnel barrier quality and thickness in governing the spin valve performance, we fabricated devices with varying Al_2O_3 deposition conditions and thicknesses. Figure S5B-E summarizes the current-voltage (I-V) characteristics of spin valves employing four different barrier configurations: 2 nm Al_2O_3 deposited at room temperature, 1 nm Al_2O_3 deposited at 200 °C, 2 nm Al_2O_3 deposited at 200 °C, and 2.5 nm Al_2O_3 deposited at 200 °C. Room-temperature deposition of 2 nm Al_2O_3 (Fig. S5B) produces a highly resistive and noisy interface, with current levels in the hundreds of nA range and significant instability across the measured voltage window. This behavior is consistent with poor atomic layer deposition (ALD) quality at room temperature resulting in resistive Al_2O_3 layer. Reducing the barrier thickness to 1 nm while using elevated deposition temperature (200 °C, Fig. S5C) leads to a barrier that is too thin to sustain a well-defined tunnel junction. The device exhibits breakdown within one to two I-V sweeps, as evidenced by the marked difference between the first and second sweep, indicating irreversible dielectric breakdown or filament formation through the ultrathin oxide. The 2 nm Al_2O_3 barrier deposited at 200 °C (Fig. S5D) represents the optimal configuration. The I-V characteristics are smooth, stable, and reproducible across multiple sweeps, with well-defined tunneling behavior and clear separation between the B+ and B- curves, corresponding to a robust magnetoresistance response. Current levels in the tens of microampere range indicate a well-conducting yet barrier-limited junction, consistent with coherent tunneling through a high-quality, pinhole-free oxide. This is the barrier configuration employed for all spin valves with a tunneling layer. Increasing the barrier thickness to 2.5 nm at 200 °C (Fig. S5E) still yields a functional spin valve with distinguishable B+ and B- curves, but the I-V characteristics become noticeably noisier, and the current levels decrease significantly compared to the 2 nm case, reflecting increased interfacial resistance. These results demonstrate that the spin valve performance is highly sensitive to both the thickness and deposition quality of the Al_2O_3 tunnel barrier. A 2 nm barrier deposited at 200 °C represents the optimal balance between barrier integrity, tunneling conductance, and device stability. A leaky or discontinuous barrier decreases the spin selectivity by providing spin-independent conduction pathways.

MR induced by magnetic proximity effect

Coupling between spin and charge transport arises at the interface of a FM and paramagnetic metal due to unequal spin-up and spin-down sub-bands in the FM (*74*). Similarly, an interface between FM and antiferromagnet can create an exchange spring in the antiferromagnet due to the coupling

to FM (*75*). Furthermore, enhanced circularly polarized emission from FM/CMHS bilayers has been reported that was attributed to the formation of these chiral/FM interfaces, serving as another manifestation of the CISS effect (*76*).

We fabricated macroscopic (*R/S*-MBA)$_2$PbI$_4$ spin valves without an Al_2O_3 tunneling barrier which allows for a direct contact between the FM and CMHS layer (Fig. S11A-B). Without the tunneling barrier, an ohmic *I-V* characteristic is observed (*77*). The current is in the mA range and not in the μA range observed from the corresponding spin valves containing the tunnel barrier. This larger current arises because of short circuits between the top Au contact and bottom Ni contact. The bottom Ni atoms can diffuse into the CMHS layer due to vacancies and chemical reactions adjacent to the metal/CMHS interface, resulting in short circuit. To confirm the formation of metal interdiffusion, the FM/CMHS interfaces were examined by depth-dependent elemental composition profiling using time-of-flight secondary ion mass spectrometry (TOF-SIMS). The presence of the tunneling barrier creates a sharp well-defined interface between the CMHS and FM layer. In contrast, the absence of tunnel barrier results in substantial interfacial mixing of CMHS and FM layers (Fig. S11C, Fig. S12).

For the *I-V* characteristics measured at ± 1 T, the barrier free (*R*-MBA)$_2$PbI$_4$ device shows a higher and lower resistance under -B and +B, respectively (Fig. S11D gray vs. green-traces). Conversely, when the handedness of the CMHS layer is inverted, the opposite trend is observed (Fig. S11E). It is noteworthy here that there is still a MR response even though *no current* passes through the CMHS owing to the filament formation. We confirmed that a spin valve without having the CMHS layer, i.e., Au/Ni interface, does not show any difference in their *I-V* characteristics under +B and -B (Fig. S13). In addition, we verified the short circuits in the device by measuring the anisotropic magnetoresistance (AMR) signals which is a fundamental characteristic of FM (Ni) layer. The MR(B) curve in the Ni/CMHS/Au device exhibits a characteristic butterfly-shaped curve, with a hysteresis dip around B = ±0.1 T, corresponding to the coercive field of Ni (Fig. S11F bottom panel). We found the CMHS interacts with the FM through the MIPAC effect revealing two key features: (1) the MR(B) loop exhibits asymmetry from -B to +B (Fig. S11F top panel compare green vs gray at -1 T and +1 T) and (2) the coercive field of Ni decreases (dashed line in Fig. S11F bottom to top panels compare bare Ni to CMHS/Ni). Figure S14 shows additional I-V characteristics of spin valves without the tunneling layer. To further validate the MIPAC effect, an axially chiral molecule (R-BHP) was tested, and similar effects were observed. Additionally, when a PEDOT:PSS coating was applied to the FM layer prior to R-BHP deposition, the MIPAC effect was suppressed and the current level decreased, confirming the interfacial origin of the MIPAC effect (Fig. S15).

Tunneling current through a rectangular barrier

The spin valve can be described in terms of a ferromagnet-oxide-semiconductor tunneling junction in the low bias regime (*78*). The ferromagnet and the semiconductor are both connected to reservoirs with their corresponding chemical potential of $\mu_L = 0$ and $\mu_R = eV$. In the regime where the applied bias is much smaller than the tunneling barrier height $eV \ll E_b$, the barrier can be approximated with a rectangular tunneling barrier. Thus, we can write the Schrödinger equation of the system as:

$$\left[-\frac{\hbar^2}{2m}\frac{\partial^2}{\partial z^2} + U(z)\right]\Psi(z) = E\,\Psi(z)$$

With the potential landscape

$$U(z) = \begin{cases} 0 & z \leq 0 \\ E_b + \chi\Delta & 0 < \mathrm{z} < \mathrm{s} \\ 0 & s \leq \; z \end{cases}$$

where the chirality induced work function $\chi\Delta \ll E_b$. It depends upon the magnetization and the chirality. The wavefunction can be found using the ansatz $\Psi_{\mathrm{j}}(z) = A_j \exp\left(ik_j z\right) + B_j \exp\left(-ik_j z\right)$ where $j = L, b, R$ together with requirement that the wave function is continuous as probability flux is conserved.

The wave vector for the barrier, the left and right contact are

$$\kappa \; = \sqrt{\frac{2m(E_b + \chi\Delta - \mathrm{E})}{\hbar^2}}$$

$$k = \; k_L = k_R = \sqrt{\frac{2mE}{\hbar^2}}$$

We are only interested in the transmission from left to right, where the electron from the left has an amplitude of $A_L = 1$, the reflected amplitude is $B_L = r$ and the transmitted amplitude is $A_R = t$ and $B_R = 0$ since no wave is coming from the right.

For the first interface,

$$\Psi_{\mathrm{L}}(z = 0) = \Psi_{\mathrm{b}}(z = 0) \qquad \rightarrow \qquad 1 + r = \; A_b + B_b$$

$$\left.\frac{\partial \Psi_{\mathrm{L}}}{\partial z}\right|_{z=0} = \left.\frac{\partial \Psi_{\mathrm{b}}}{\partial z}\right|_{z=0} \qquad \rightarrow \qquad ik\,(1 \; - r) \; = \; \kappa \; (A_b - B_b)$$

and for the second interface:

$$\Psi_{\mathrm{b}}(z = s) = \Psi_{\mathrm{R}}(z = s) \qquad \rightarrow \qquad t \exp(ik\,s) = \; A_b \exp(\kappa\,s) + B_b \exp(-\kappa\,s)$$

$$\left.\frac{\partial \Psi_{\mathrm{b}}}{\partial z}\right|_{z=s} = \left.\frac{\partial \Psi_{\mathrm{R}}}{\partial z}\right|_{z=s} \qquad \rightarrow \qquad ik\,t \exp(ik\,s) \; = \; \kappa \; \left(A_b \exp(\kappa\,s) - B_b \exp(-\kappa\,s)\right)$$

Solving for the transmission t, eliminating with some algebra yields the transmitted amplitude:

$$t = \frac{2\,\kappa\,k \exp(-iks)}{2k\kappa \cosh(\kappa s) + i(k^2 - \kappa^2)\sinh(\kappa s)}$$

The transmission probability is thus

$$T(E) = \; |t|^2 \; = \left(1 + \left(\frac{k^2 + \; \kappa^2}{2\,k\,\kappa}\right)^2 \sinh^2(\kappa\,s)\right)^{-1}$$

In the limit of the WKB approximation, at small energies ($E_b \gg \; E$) and wide barriers, $\kappa\,s \gg 1$ so that

$$T(E) \approx \; \frac{4\,k\,\kappa}{(k^2 + \; \kappa^2)} \frac{4\,k\,\kappa}{(k^2 + \; \kappa^2)} \exp(-2\kappa s),$$

$$\approx t_1 t_2 \exp(-2\kappa s)$$

where the first and second terms $t_1 = t_2 = \frac{4\,k\,\kappa}{(k^2+\kappa^2)}$ respectively represent the tunneling amplitude from the left electrode into the tunnel barrier; and from tunnel barrier to the right electrode, while the exponential term describes decay in the barrier. Written in this way, it is clear that the factorization assumed in Eq. 8 of the main text is justified (*52-54, 78*).
Adapting Eq. 20 of Simmons, (79) the current through a tunneling barrier with similar electrodes is

$$I = \frac{Ae}{2\pi\,h\,s^2}\left[(E_b + \chi\Delta)\exp\left(-2s\sqrt{\frac{2m(E_b+\chi\Delta)}{\hbar^2}}\right) - (E_b + \chi\Delta - \mathrm{eV})\exp\left(-2s\sqrt{\frac{2m(E_b+\chi\Delta-\mathrm{e}|\mathrm{V}|)}{\hbar^2}}\right)\right]$$

which can be approximated for small, but non-zero voltages as

$$I \approx \frac{Ae^2V}{4\pi^2\,\hbar\;s^2}\exp\left(-2s\sqrt{\frac{2m(E_b+\chi\Delta-\mathrm{e}|\mathrm{V}|)}{\hbar^2}}\right)$$

Since the exact surface area A of the junction is experimentally unknown, we can only describe the experimental proportionality

$$I \sim V\exp\left(-2s\sqrt{\frac{2m(E_b+\chi\Delta-\mathrm{e}|\mathrm{V}|)}{\hbar^2}}\right)$$

Electric field strength estimation

The surface potential induced by chirality-related effects can vary by up to ΔU = 100 mV relative to the Fermi level of the ferromagnetic electrode. Assuming a tunneling barrier thickness of 2 nm and the absence of mobile charges within the barrier, the resulting electric field across it is given by

$$|\vec{E}| = \frac{\Delta U}{d} = \frac{0.1\ \mathrm{V}}{2\ \mathrm{nm}} \approx 50\ \mathrm{MV/m}$$

In contrast, considering the entire device, which includes both the semiconductor and the tunneling barrier with a total thickness of 170 nm, the corresponding electric field is

$$|\vec{E}| = \frac{\Delta U}{d} \approx 0.6\ \mathrm{MV/m}$$

Continuous chirality measure

Continuous Symmetry Measure (CSM)
The continuous symmetry measure (CSM) is computed by minimizing the difference of structure with atomic coordinates (Q_k) and the coordinates (P_k) belonging to point group symmetry (G). The set of points P_k with point group symmetry G are systematically varied until $\sum_k^N |Q_k - P_k|^2$ is minimized. The minimized difference is then normalized by the center of positions vector (Q_0). For the inorganic sublattice the CCM is computed using the racemic inorganic positions as P_k. The

CCM is computed by minimizing the CSM, $S(S_n)$ with respect to the achiral point group (S_n) (*68, 69*).

$$CCM = \min[S(S_n)]$$

$$S(\mathrm{G}) = 100 \cdot \frac{\min[\sum_k^N |Q_k - P_k|^2]}{\sum_k |Q_k - Q_0|^2}$$

$$Q_0 = \frac{1}{N} \sum_k^N Q_k$$

We find that the chiral molecules tend to have larger CCM than the inorganic layers and do not alone predict the amount of chirality in the inorganic layer. For instance, 3BrMBA has the largest CCM but introduces the least chirality into the inorganic layer. Additionally, MBA introduces more chirality into the Sn based perovskite than the Pb counterpart.

Field-dependent MR

The electronic stability of CMHS needs to be considered for field-dependent measurements as current drift or device degradation can occur under prolonged electrical bias. To minimize these effects, resistance was measured only at a limited set of magnetic field values to construct the field-dependent MR curve. Because Ni electrodes exhibit negligible magnetic hysteresis, all measurements were performed while sweeping the field in a single direction, which provides sufficient data points to reproduce the expected Ni magnetization profile. For field dependent measurements, robust spin valves with negligible current drift and clear difference between multiple cycles of positive and negative fields were selected. An example of such a spin valve is shown in Fig. S29. It is important to note that CMHS-based spin valves can become electronically unstable when subjected to repeated magnetic field sweeps, likely due to ion migration or interfacial degradation. To avoid electrical overstressing, resistance measurements should be performed using minimized dwell times and controlled field sweep rates. Developing optimized MR measurement protocols specifically tailored for CMHS devices will therefore be essential to achieve reliable and reproducible field-dependent magnetoresistance in future studies.

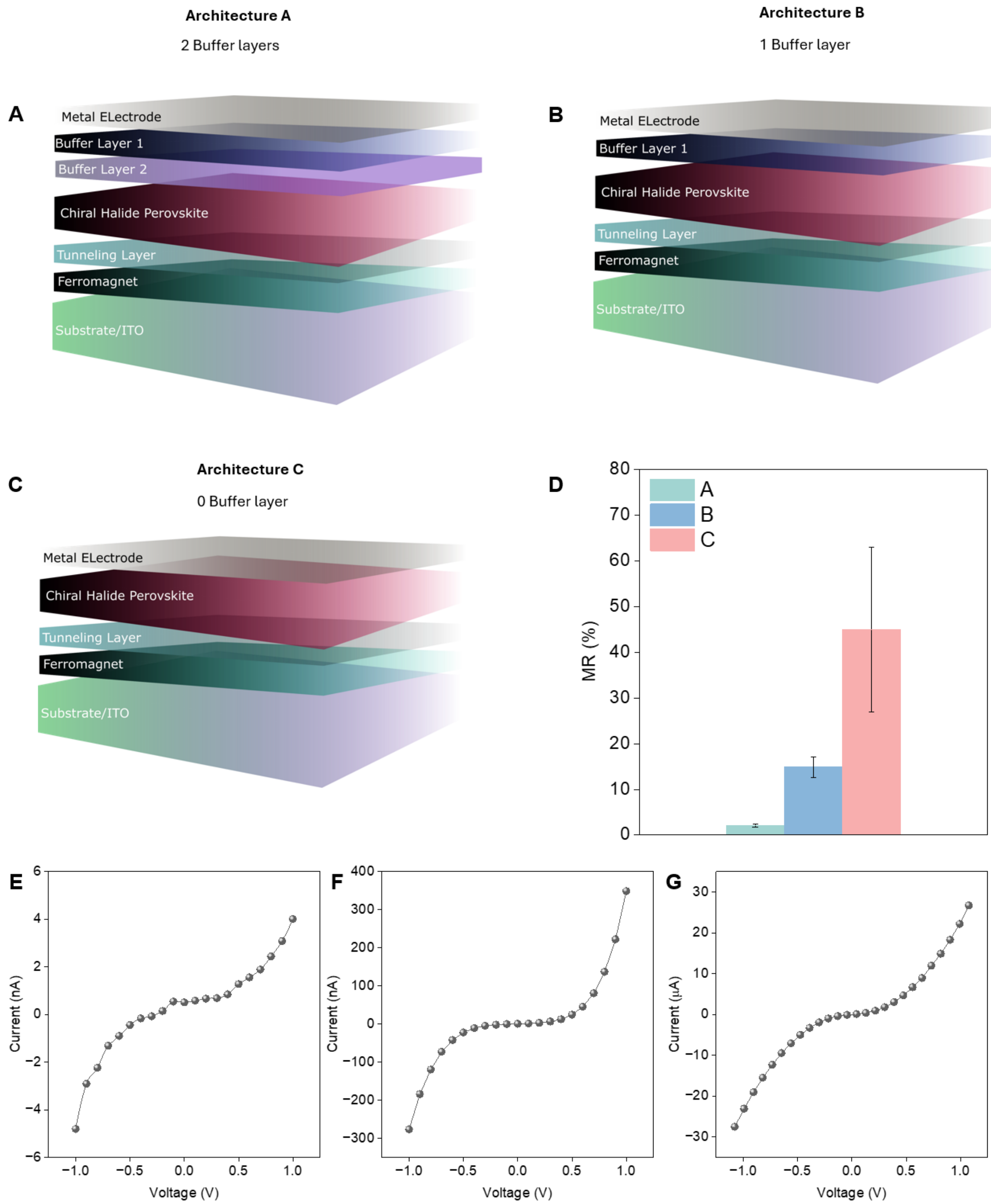

Fig. S1. CISS-based spin valve architecture. (**A**) Two buffer layers. (**B**) A single buffer layer. (**C**) No buffer layer between the CMHS and metal electrode interface. (**D**) Average MR response as a function of spin valve architecture for (R-MBA)$_2$PbI$_4$. Buffer layer 1 and 2 represent MoO_x and BCP, respectively. (**E-G**) *I-V* characteristics for spin valves with A, B, and C architecture, respectively. The current level increases with decreasing number of interfaces.

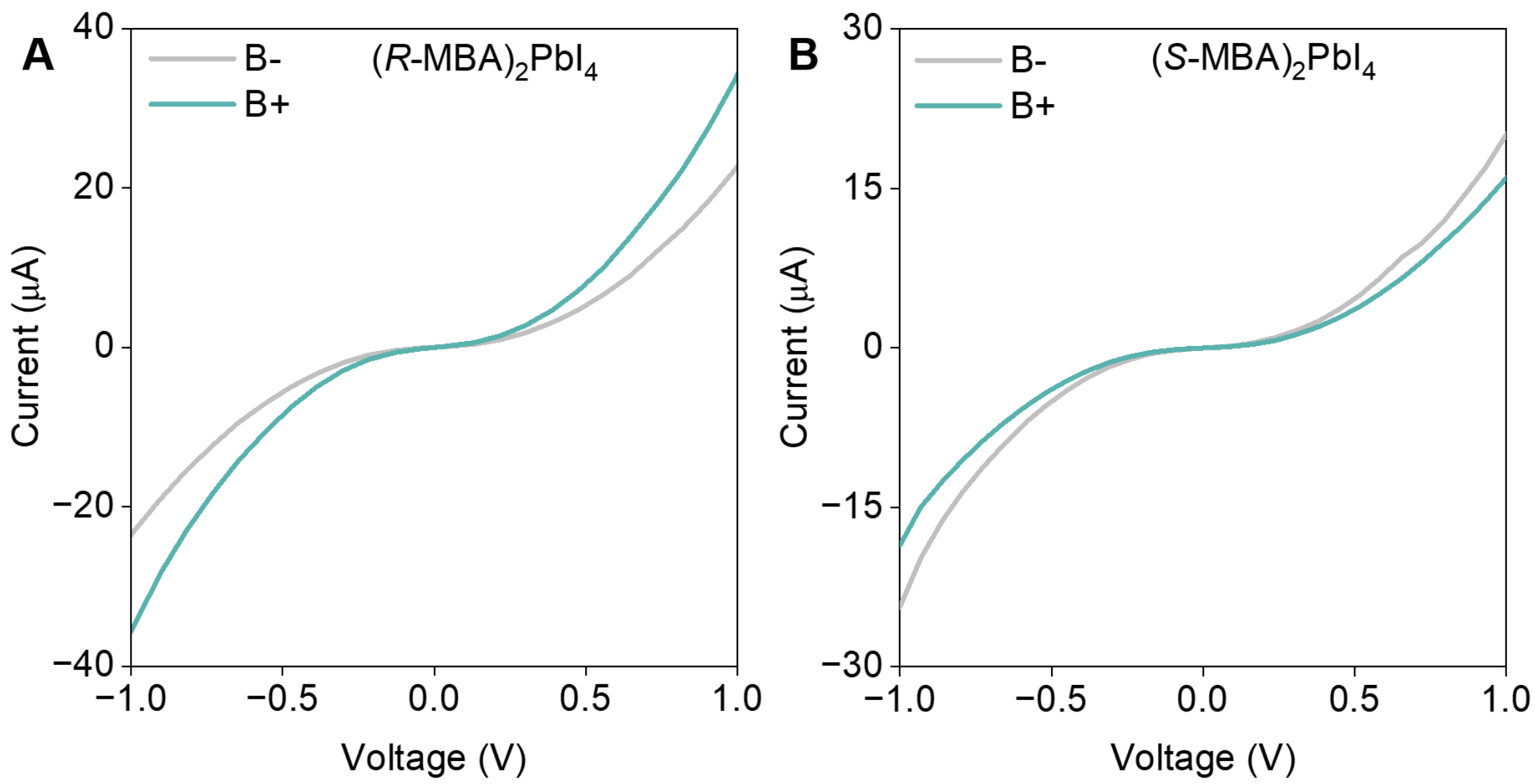

Fig. S2. Room-temperature current-voltage characteristics. (**A**) (*R*-MBA)$_2$PbI$_4$ and (**B**) (*S*-MBA)$_2$PbI$_4$. The spin valves (FM/Al_2O_3/CMHS/Au) are from the same device batch and show opposite response under out of plane magnetic field (±1 T) depending on the CMHS handedness.

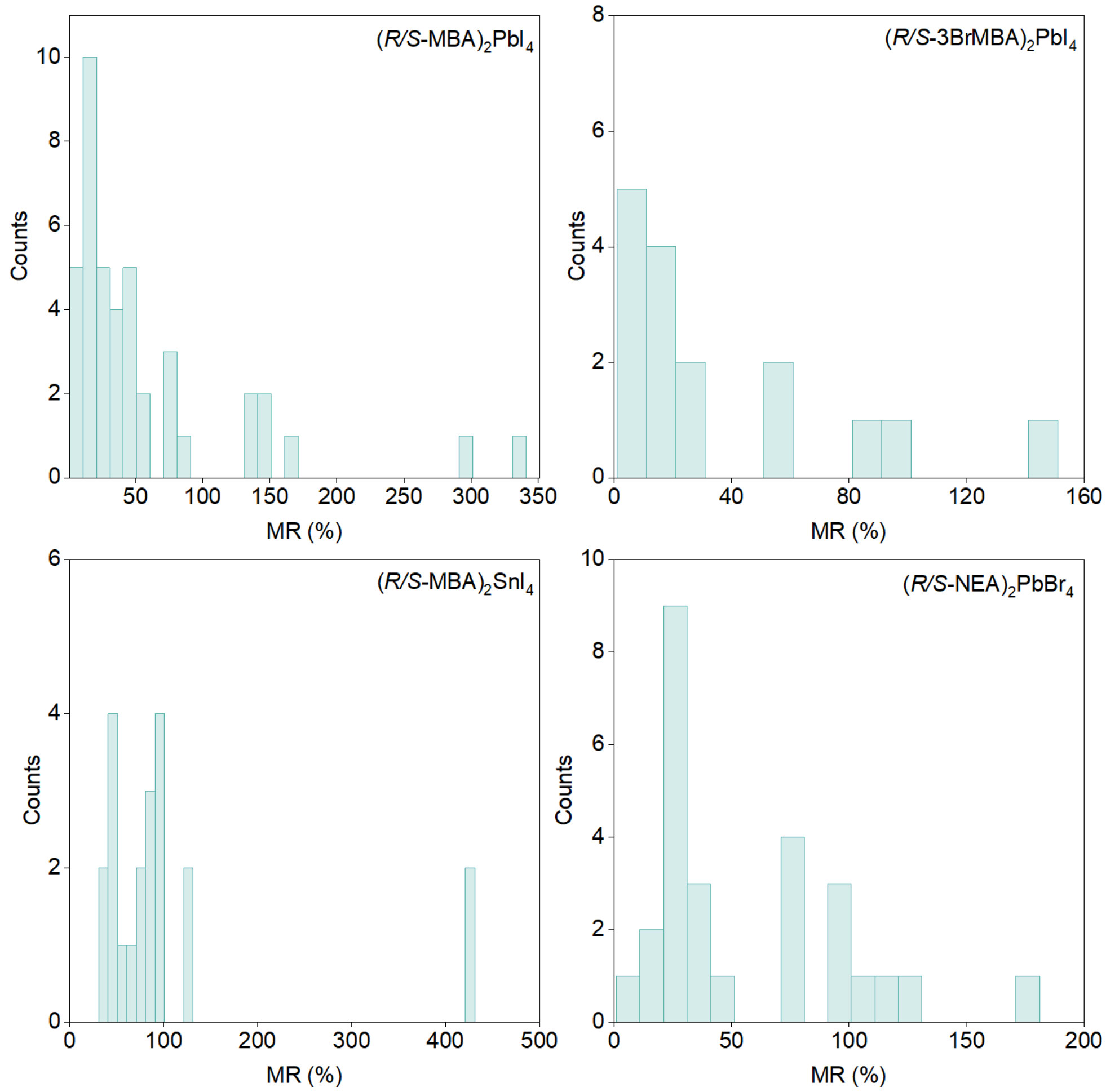

Fig. S3. MR histogram. MR distribution of spin valves for different CMHS compositions.

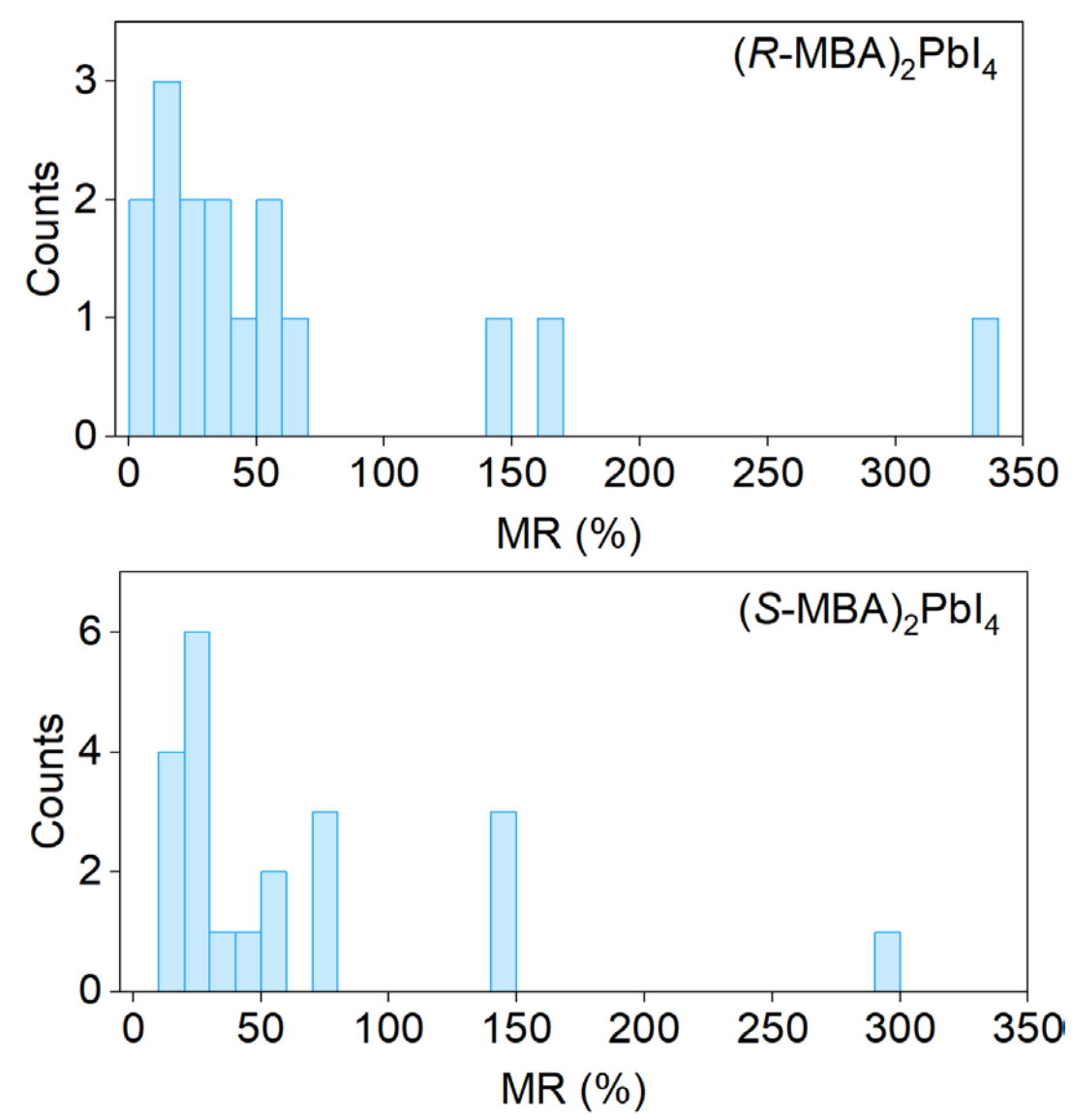

Fig. S4. MR histogram. MR distribution of spin valves for $(R/S\text{-}MBA)_2PbI_4$ compositions.

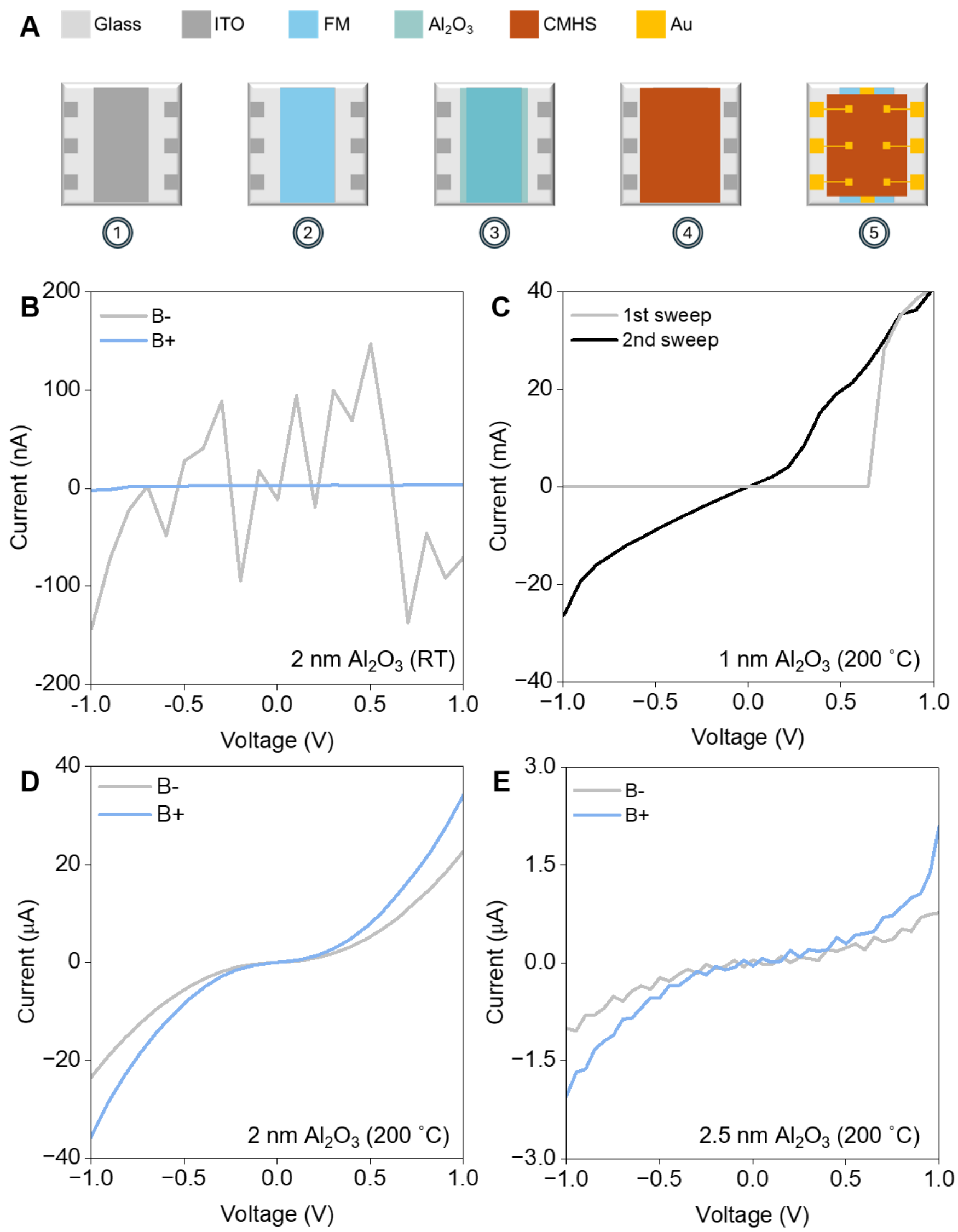

Fig. S5. Spin valve fabrication and tunnel layer optimization. (**A**) Spin valve fabrication steps. I-V characteristics of $(R\text{-}MBA)_2PbI_4$ spin valves under varying Al_2O_3 tunnel barrier deposition conditions and thickness. (**B**) Room-temperature (RT) deposited Al_2O_3. (**C**) 1 nm Al_2O_3 deposited at 200 °C. (**D**) 2 nm Al_2O_3 deposited at 200 °C. (**E**) 2.5 nm Al_2O_3 deposited at 200 °C.

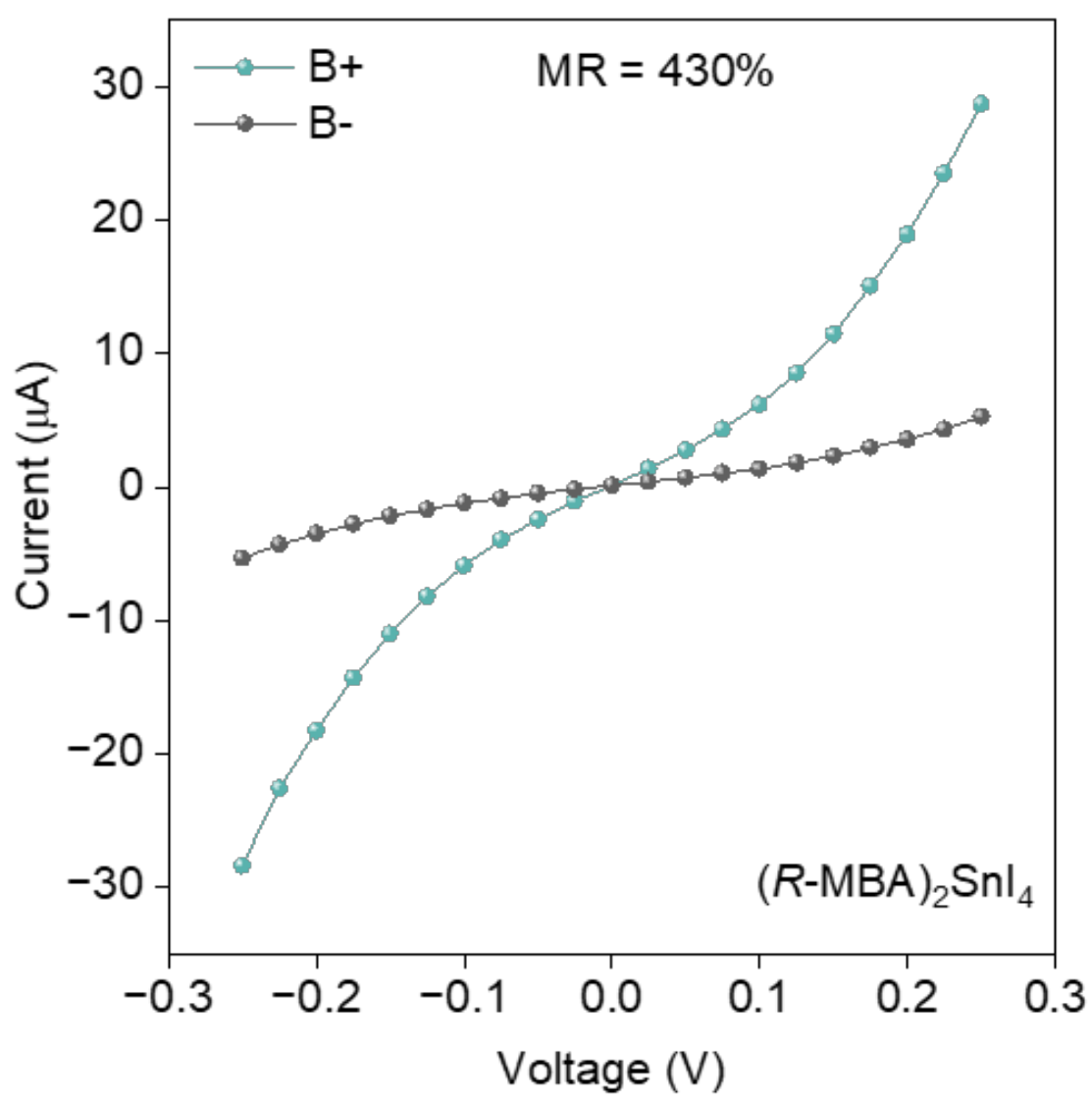

Fig. S6. Current-Voltage characteristics. I-Vs of high MR spin valves (FM/Al_2O_3/CMHS/Au) for (*R*-MBA)$_2$$SnI_4$ and (*S*-NEA)$_2$$PbBr_4$.

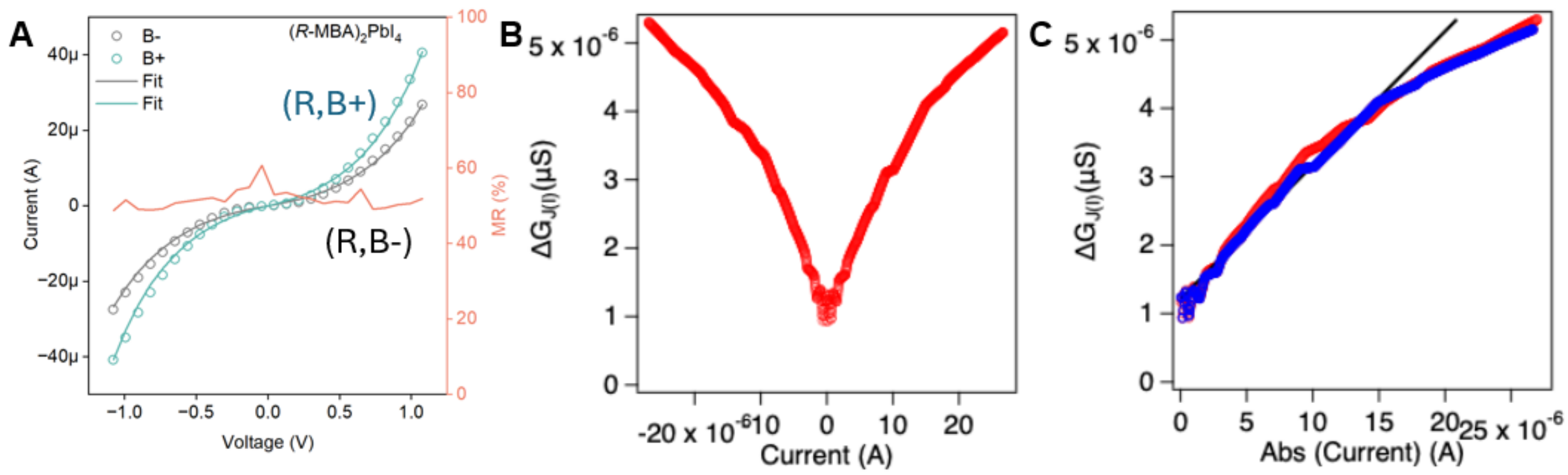

Fig. S7. Analysis of current-voltage characteristics. (**A**) Fitting of the experimental data for (*R*-MBA)$_2$PbI$_4$-based spin valve (FM/Al$_2$O$_3$/CMHS/Au) by a model assuming tunneling current through a rectangular barrier. MR values calculated from *I-V* characteristics are also shown. ΔG_J as a function of (**B**) current and (**C**) absolute current for (*R*-MBA)$_2$PbI$_4$.

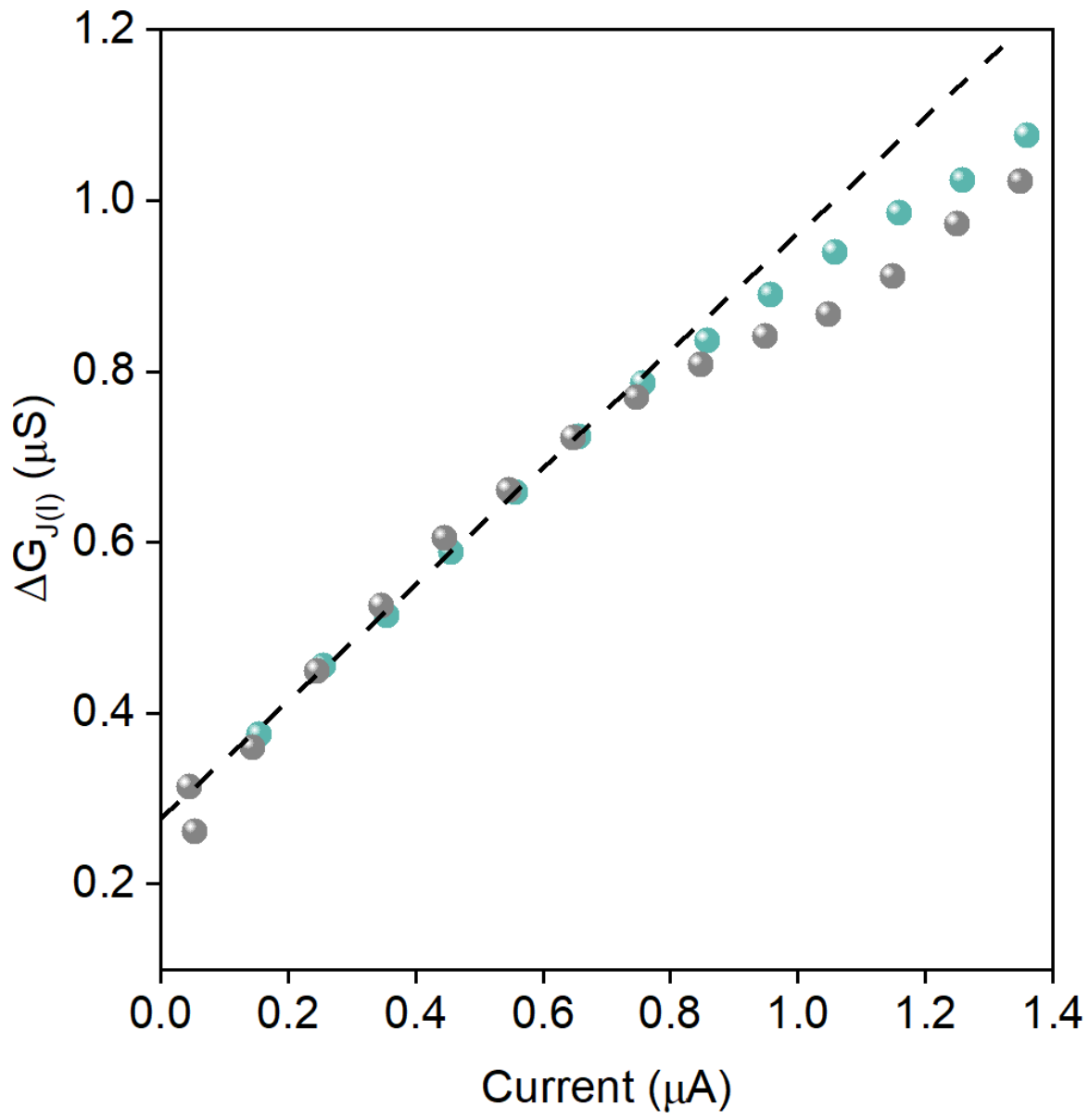

Fig. S8. Current-dependent junction magnetoconductance. ΔG_J as a function of absolute current for (*S*-MBA)$_2$PbI$_4$ spin valve (FM/Al_2O_3/CMHS/Au).

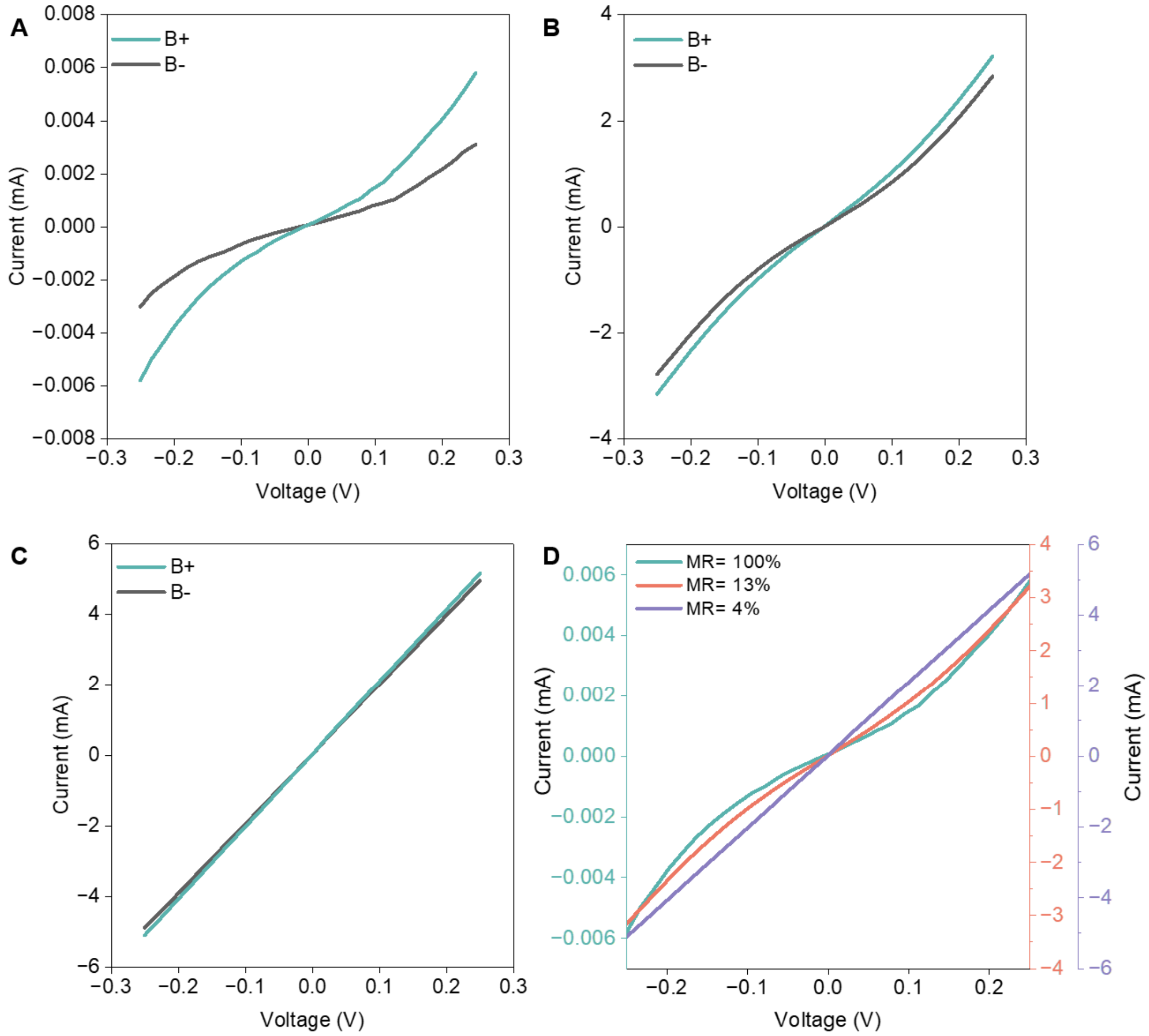

Fig. S9. Current-voltage characteristics. (**A-C**) (R-MBA)$_2$SnI$_4$ spin valves (FM/Al_2O_3/CMHS/Au) with different strengths of tunnel junction. (**D**) Comparison of positive field I-V from A-C. MR decreases for leaky spin valves.

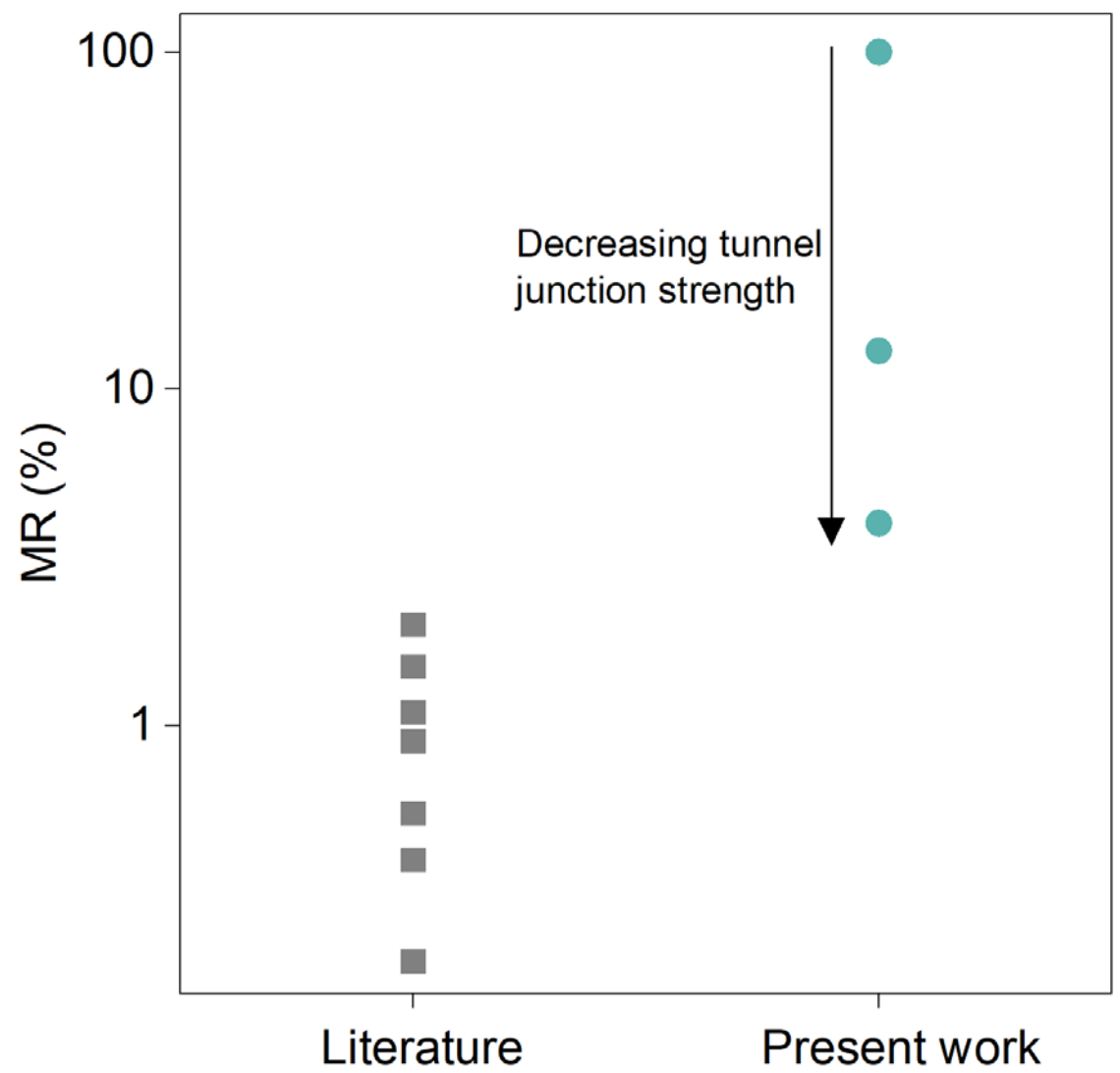

Fig. S10. Comparison of MR values. MR for various CMHS from literature (*17-20*) and the present work for (*R*-MBA)$_2$SnI$_4$ spin valves with different degrees of tunnel junction strength.

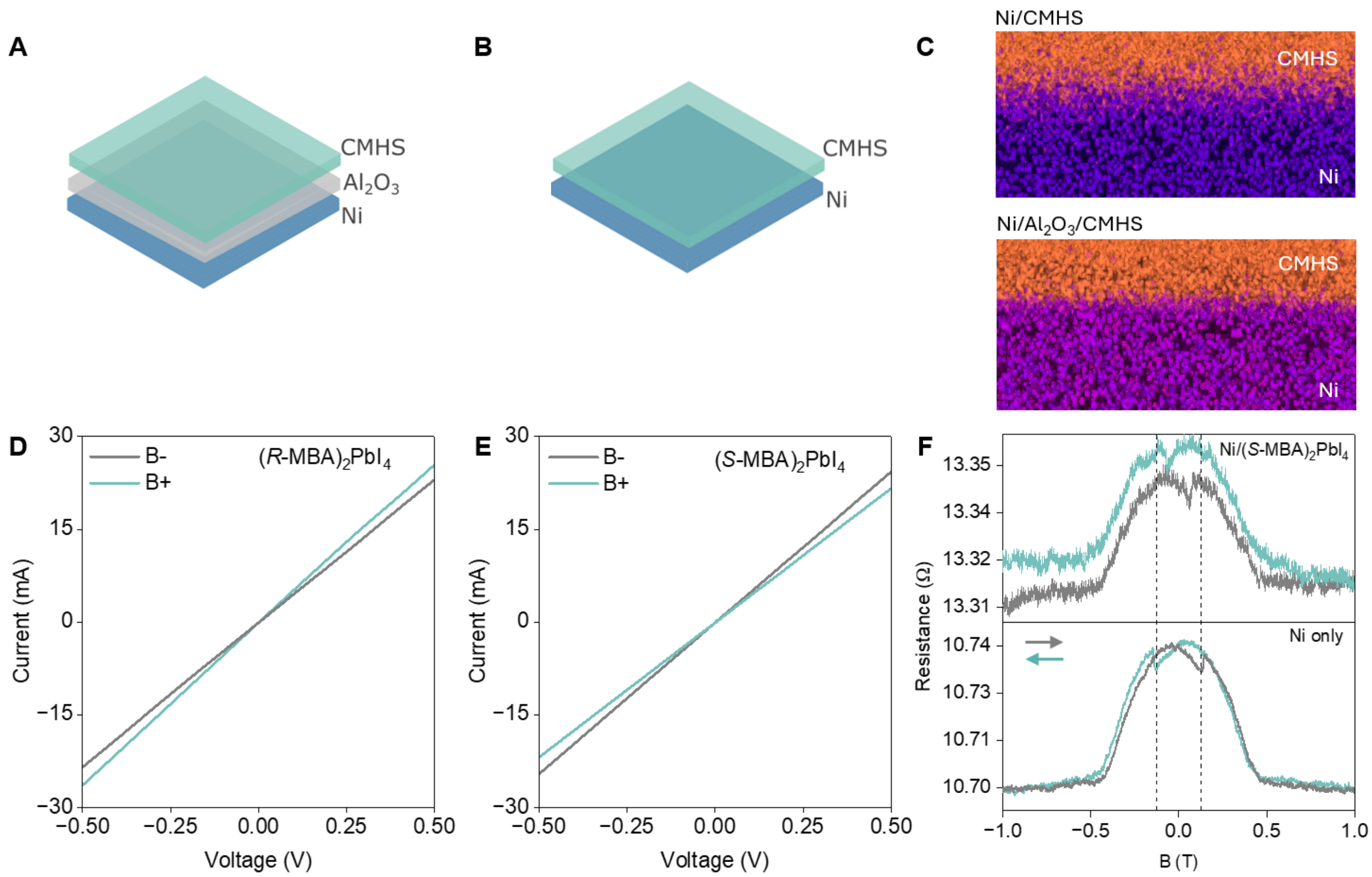

Fig. S11. Spin-polarized current exchange between CMHS and ferromagnet. (**A-B**) Schematic showing the interface between the CMHS and the ferromagnet with and without Al_2O_3. For a typical CISS spin valve, there is an ultrathin tunneling layer present between the FM and active layer to mitigate conductivity mismatch. (**C**) 3D rendering of TOF-SIMS for Ni/CMHS interface in the spin valve without and with Al_2O_3 layer. Orange and purple represent Iodine and Ni, respectively. The absence of a tunneling layer leads to the mixing of Ni and CMHS while in the presence of Al_2O_3, a sharp interface is observed between Ni and CMHS. TOF-SIMS measurements were carried out after the electrical characterization of the spin valves. Current-voltage characteristics of tunneling layer-free spin valves ($B\pm = \pm 1$ T) of (**D**) (*R*-MBA)$_2$PbI$_4$ and (**E**) (*S*-MBA)$_2$PbI$_4$ CMHS. (**F**) Magnetic-field-dependent response of spin valve without tunneling layer for Ni only and with (*S*-MBA)$_2$PbI$_4$ at -0.5 V. I-V curves in (**D-E**) and magnetic field response (upper panel) in (**F**) are from different spin valves.

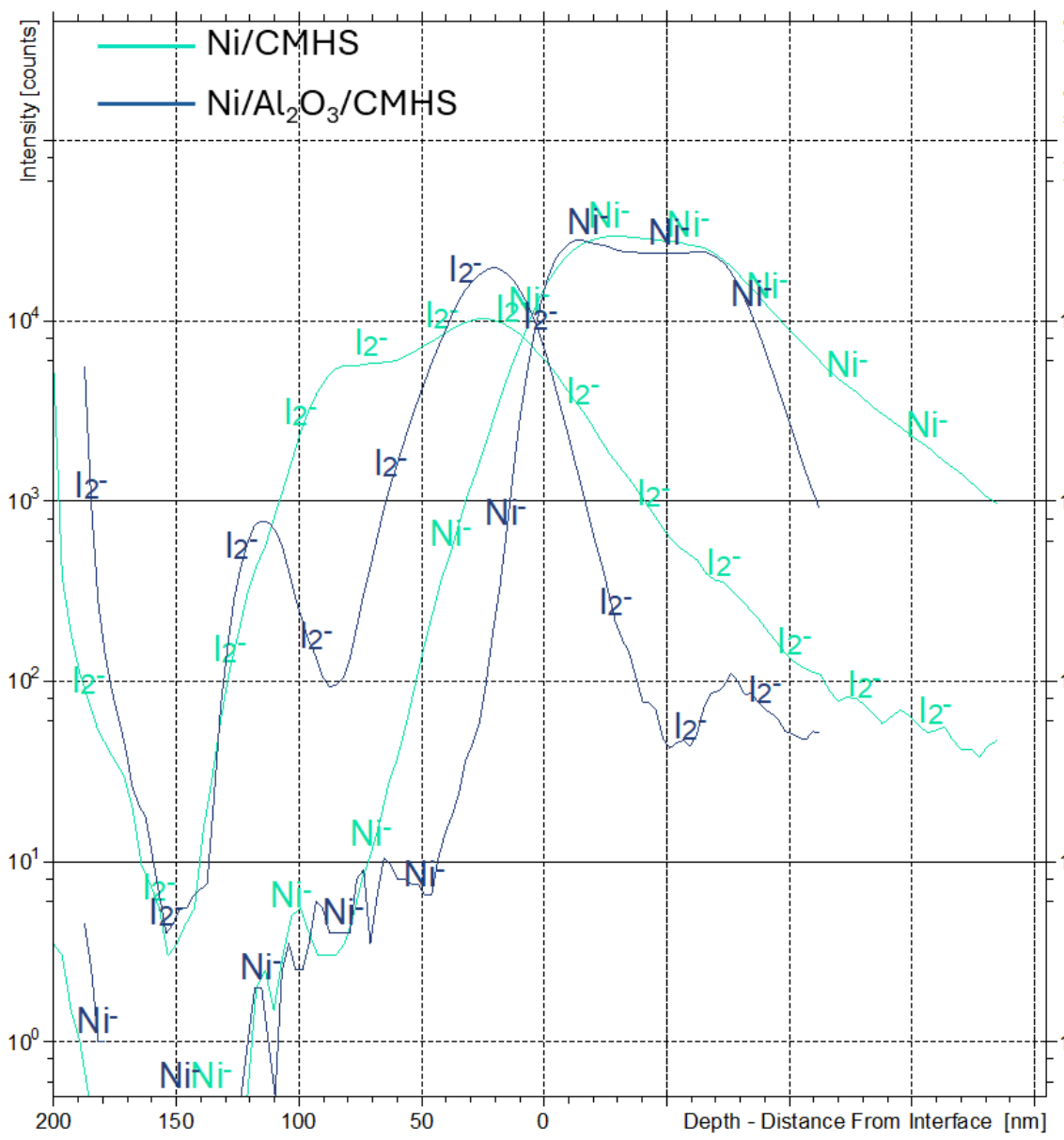

Fig. S12. TOF-SIMS depth profile. Ni/CMHS interface in the spin valve without and with Al_2O_3 layer.

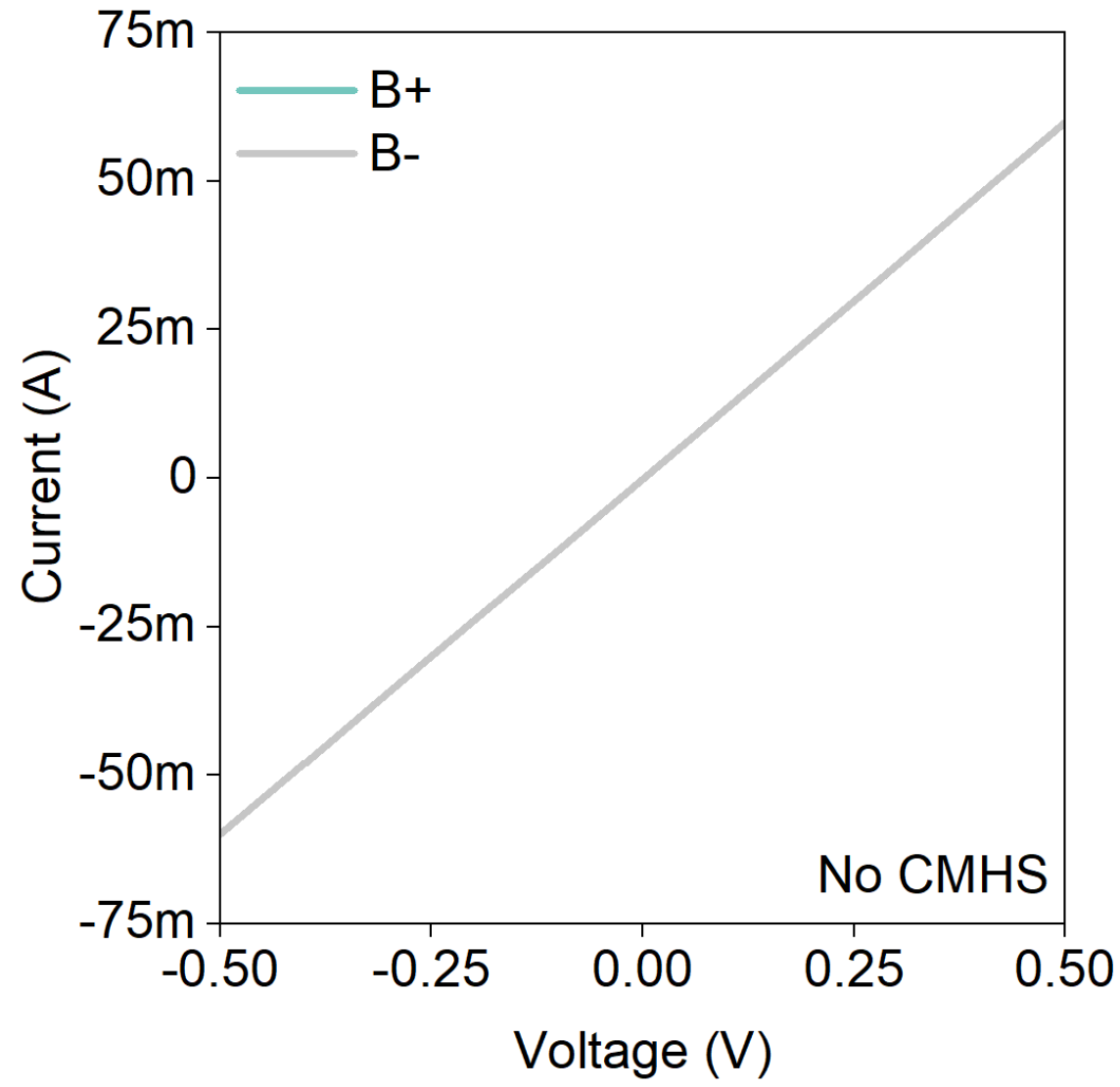

Fig. S13. Current-voltage characteristics. Spin valves without CMHS layer.

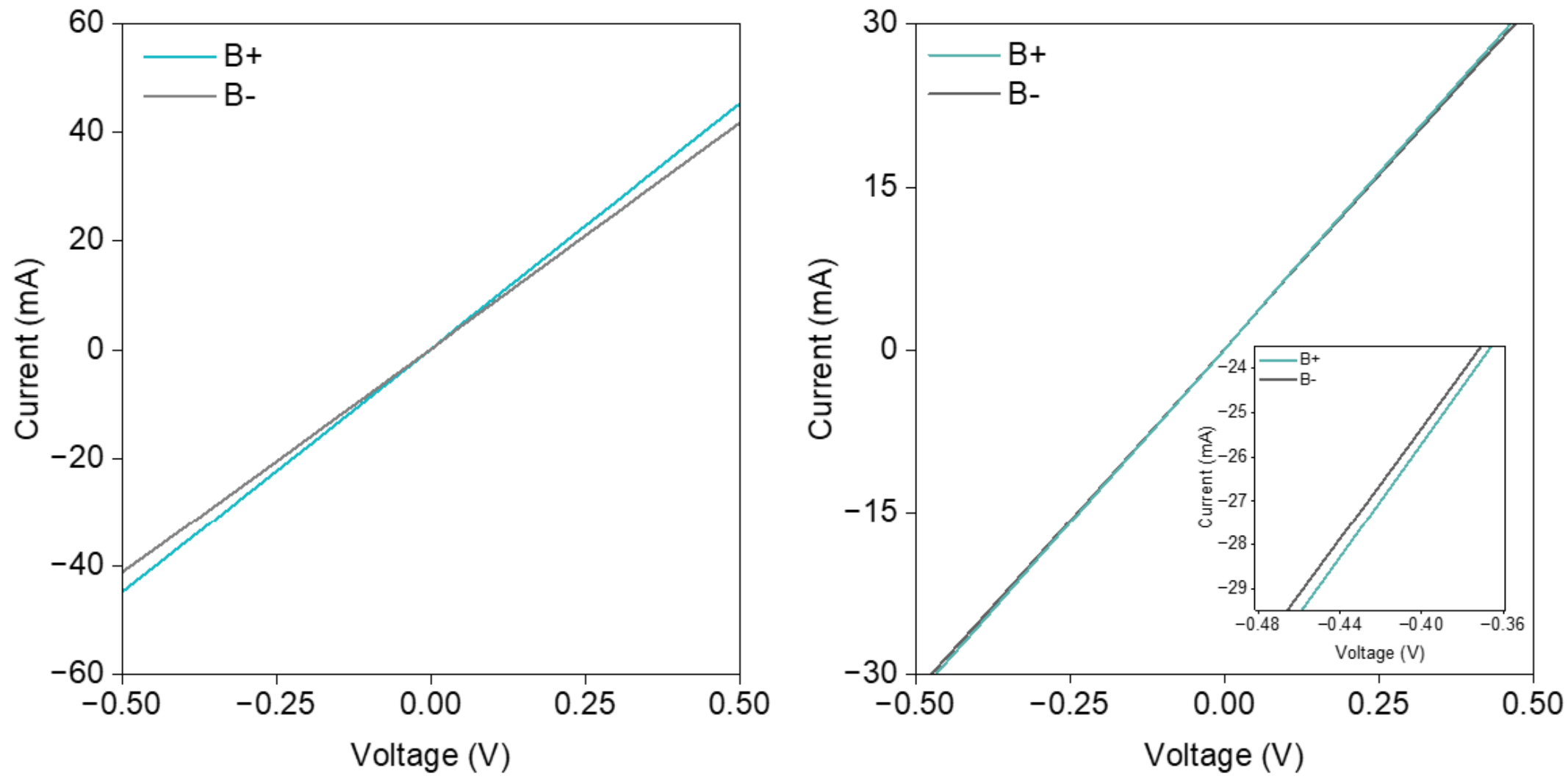

Fig. S14. Current-voltage characteristics. Tunneling layer-free spin valves (B± = ±1 T) of (*R*-MBA)$_2$PbI$_4$.

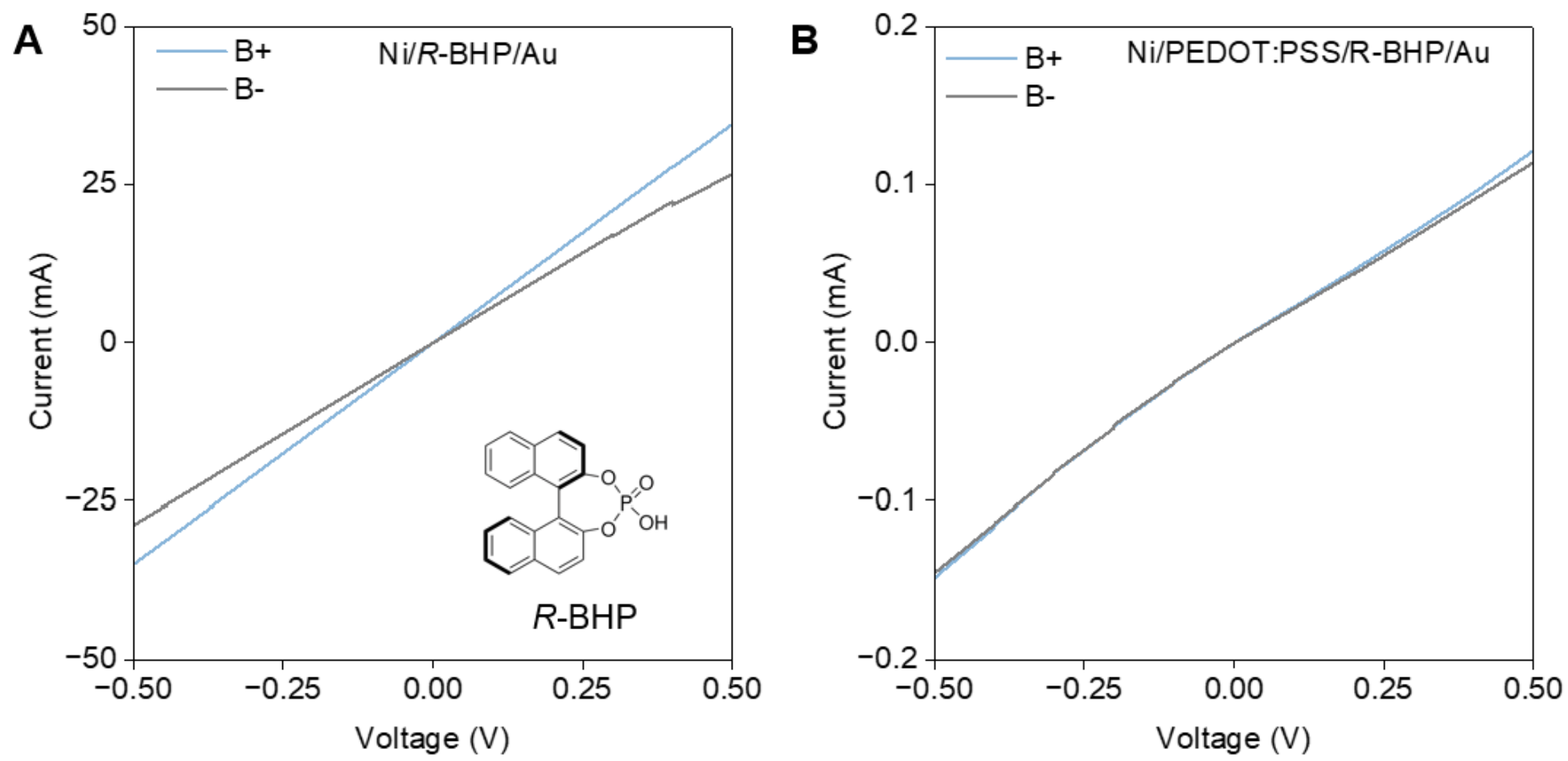

Fig. S15. Current-voltage characteristics. Devices with architecture (**A**) Ni/R-BHP/Au and (**B**) Ni/PEDOT:PSS/R-BHP/Au under magnetic field (B± = ±1 T) showing that the proximity effect vanishes when the chiral molecule and the FM layer are not in contact. R-BHP: (R)-1,1′-binaphthyl-2,2′-diyl hydrogenphosphate.

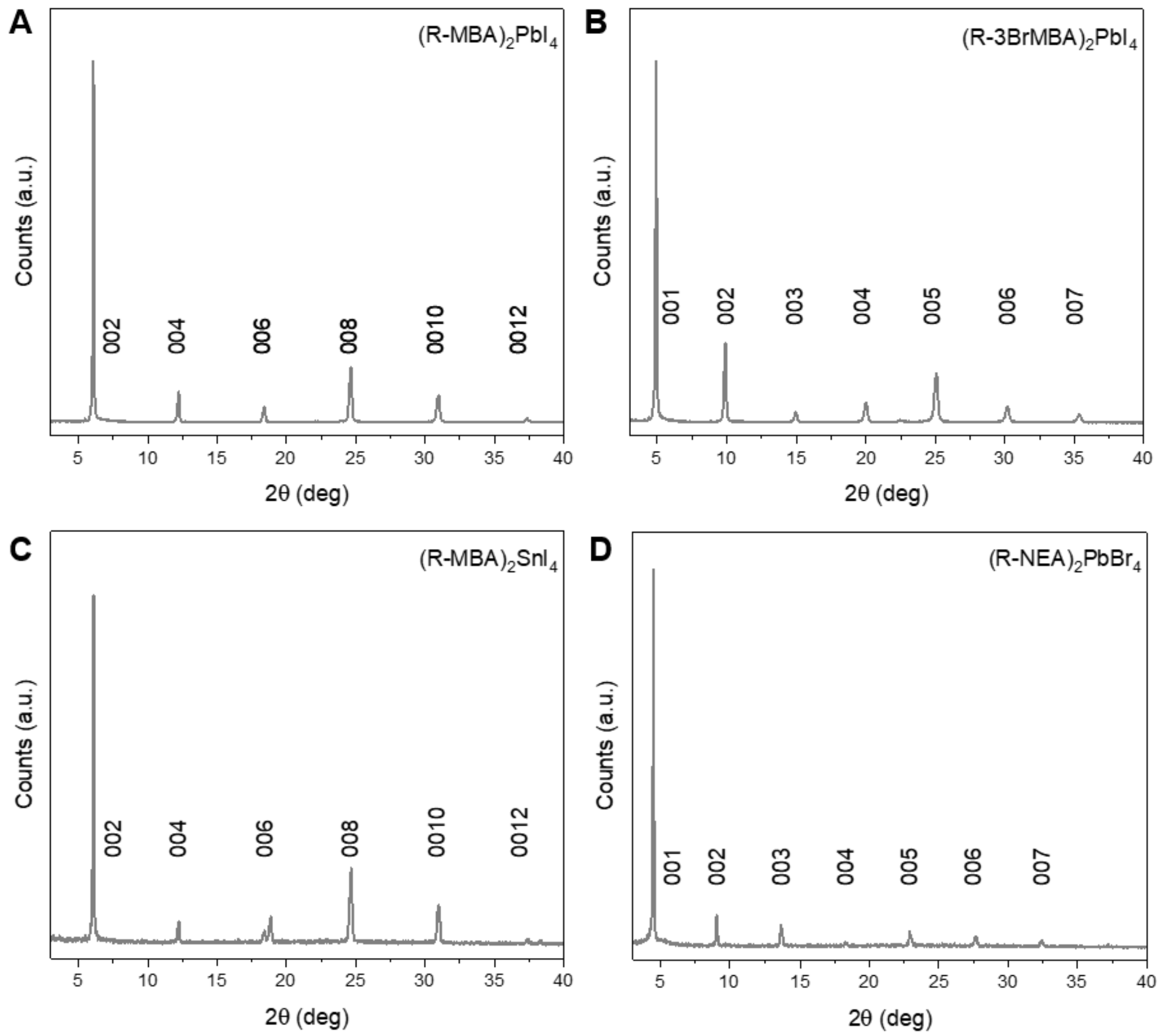

Fig. S16. XRD patterns of various CMHS films. (**A**) (*R*-MBA)$_2$PbI$_4$. (**B**) (*R*-3BrMBA)$_2$PbI$_4$. (**C**) (*R*-MBA)$_2$SnI$_4$. (**D**) (*R*-NEA)$_2$PbBr$_4$. Films were obtained by dissolving their respective single crystals in DMF and spin cast.

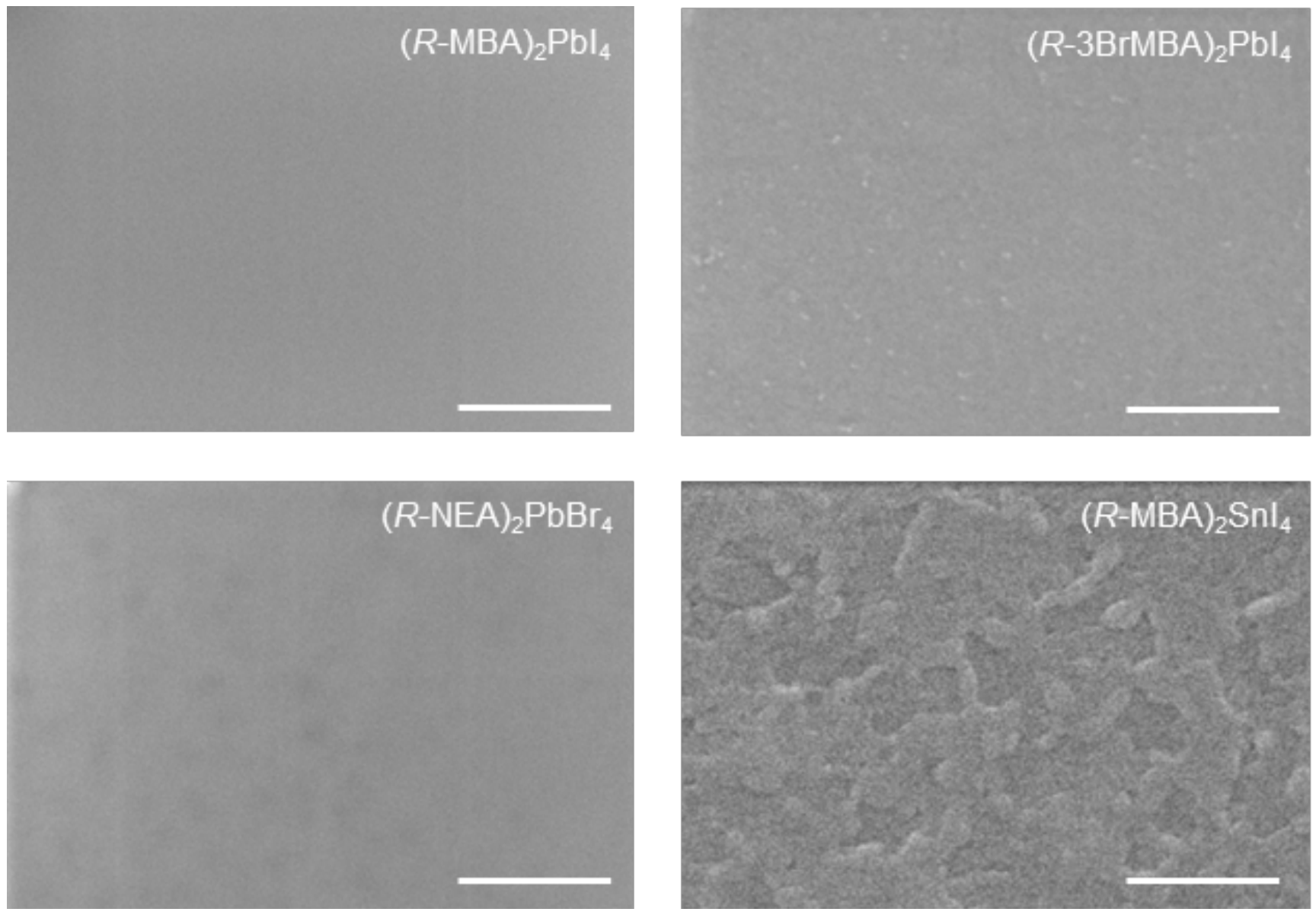

Fig. S17. Top surface SEM images. Various CMHS layers coated on FM/Al_2O_3. The scale bar is 1 μm.

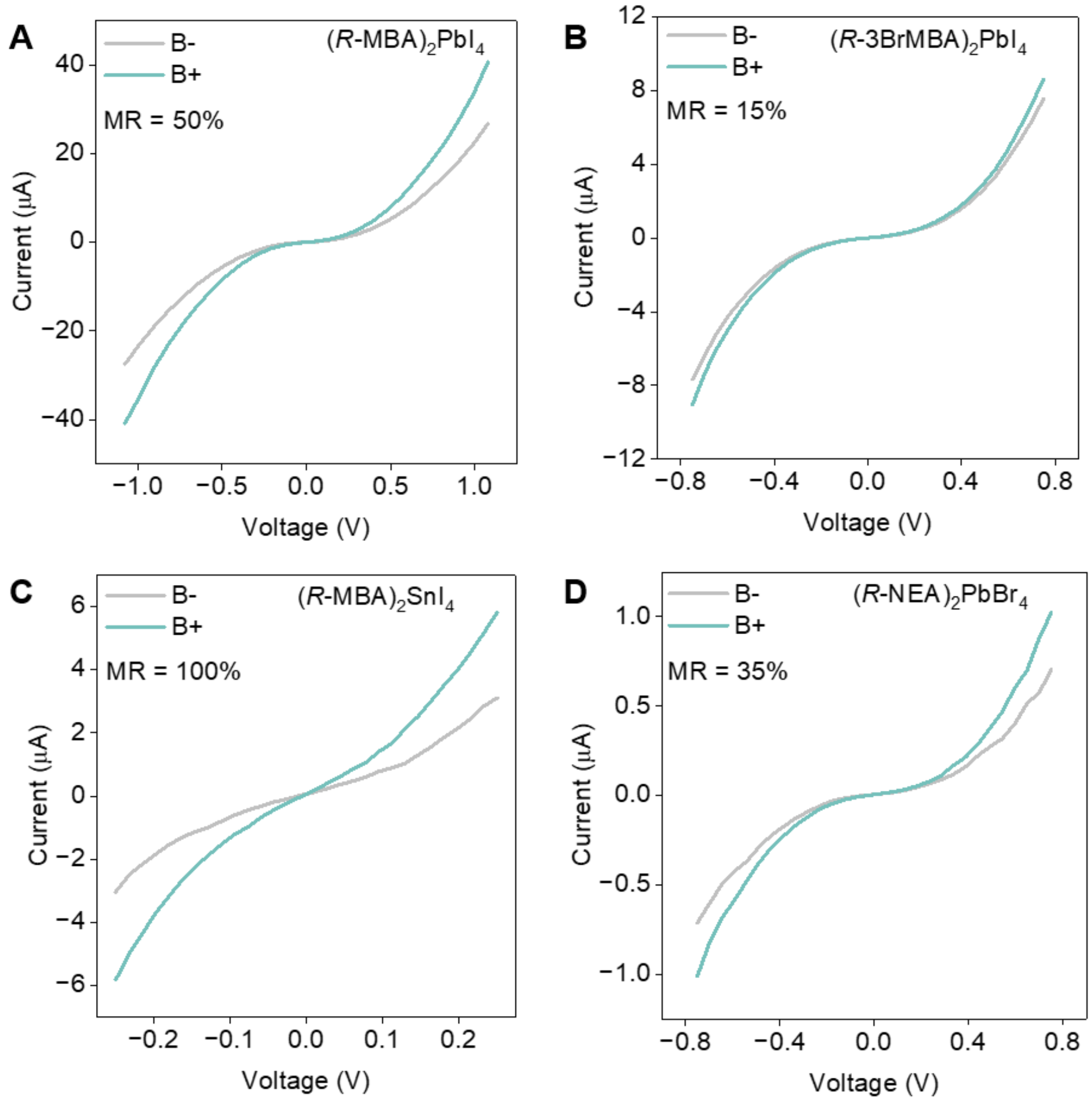

Fig. S18. Representative room-temperature current-voltage characteristics for spin valves (FM/Al_2O_3/CMHS/Au) employing CMHS. (**A**) (*R*-MBA)$_2$PbI$_4$. (**B**) (*R*-3BrMBA)$_2$PbI$_4$. (**C**) (*R*-MBA)$_2$SnI$_4$. (**D**) (*R*-NEA)$_2$PbBr$_4$ under out of plane magnetic field ($B\pm=\pm1$ T).

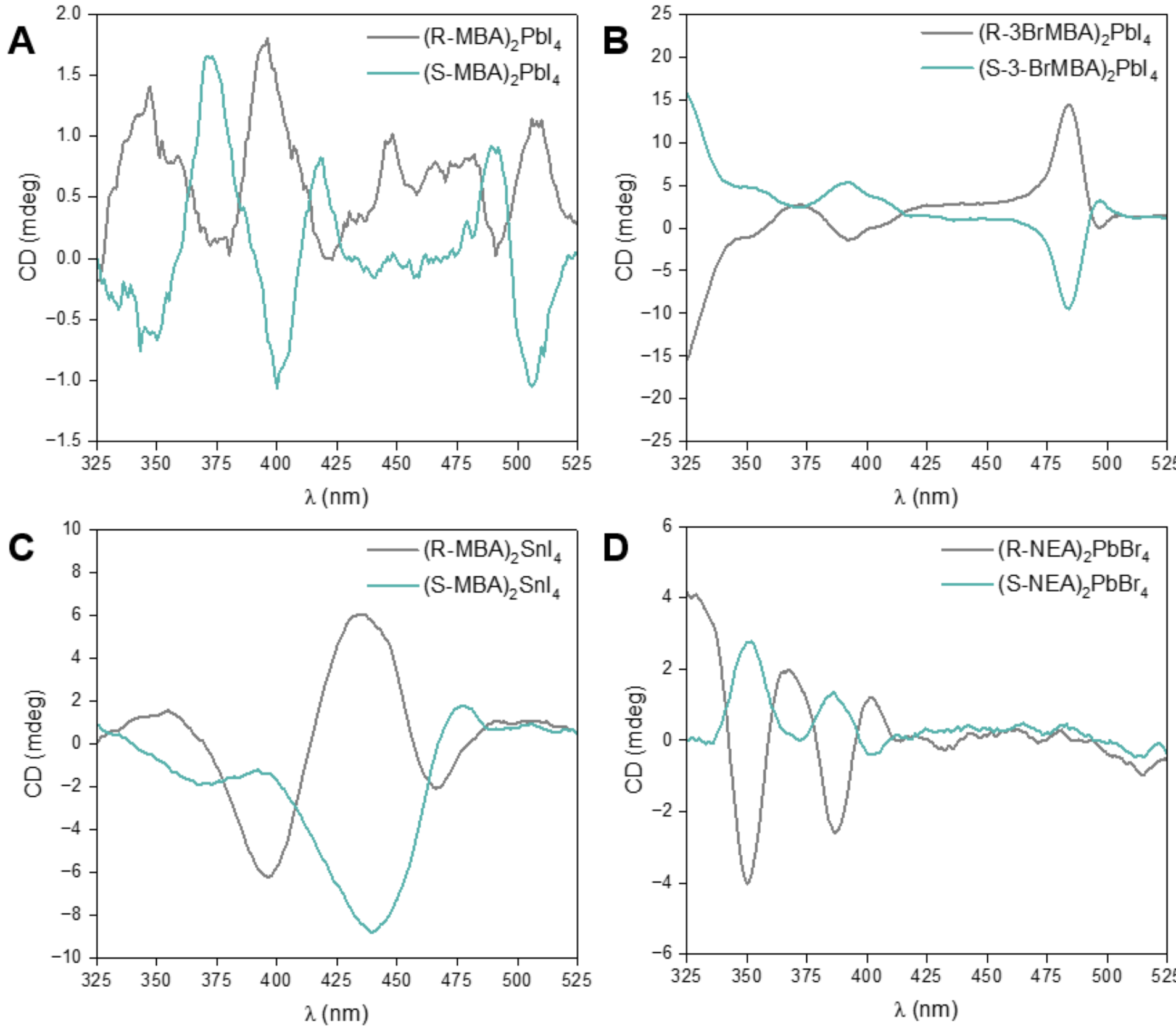

Fig. S19. CD spectra of CMHS. (**A**) (*R/S*-MBA)$_2$PbI$_4$. (**B**) (*R/S*-3BrMBA)$_2$PbI$_4$. (**C**) (*R/S*-MBA)$_2$SnI$_4$. (**D**) (*R/S*-NEA)$_2$PbBr$_4$. Films on glass were deposited with the same recipe as that of spin valves.

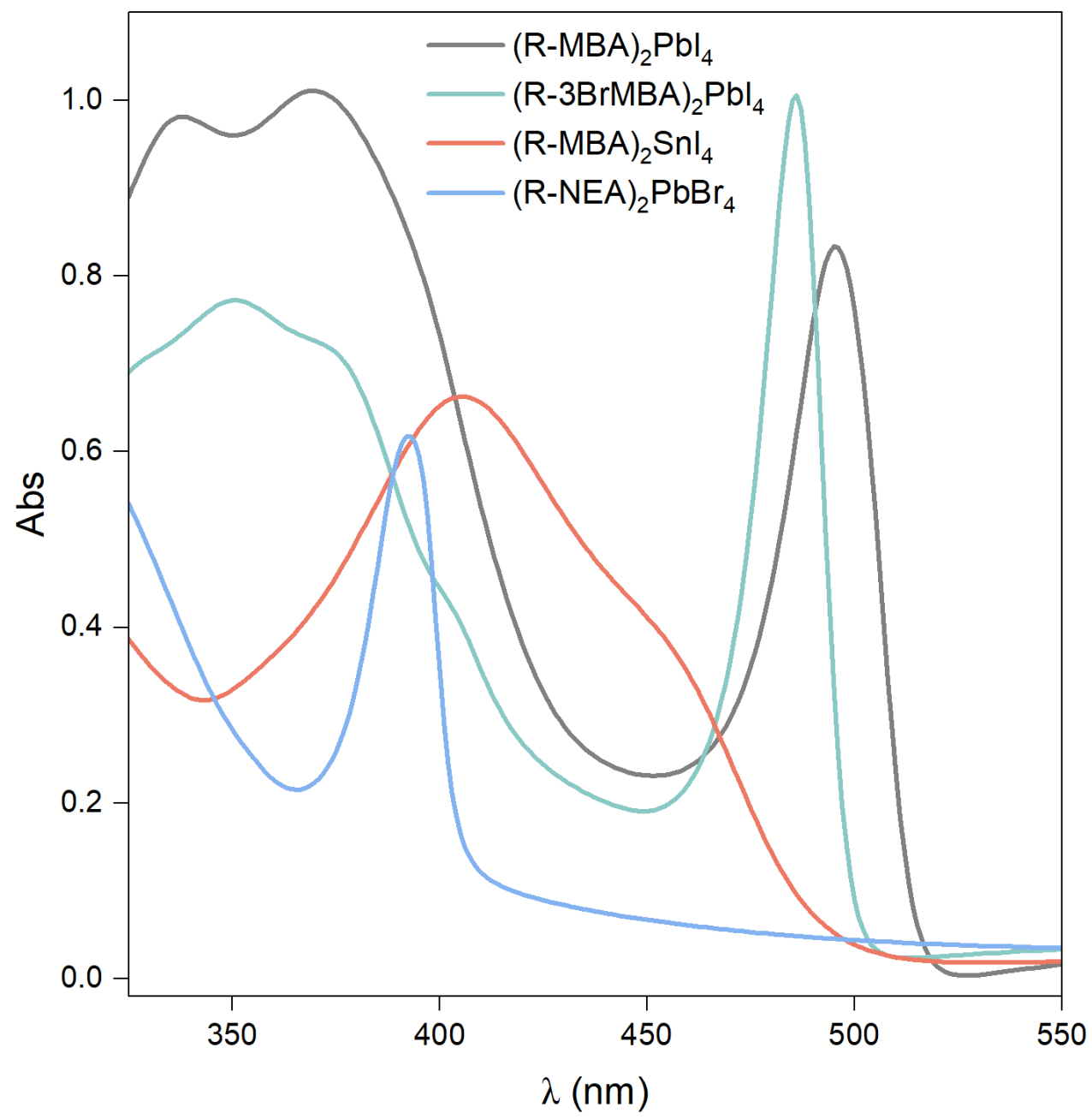

Fig. S20. Absorption spectra of CMHS. UV-Vis absorption of various CMHS compositions. Films on glass were deposited with the same recipe as that of spin valves.

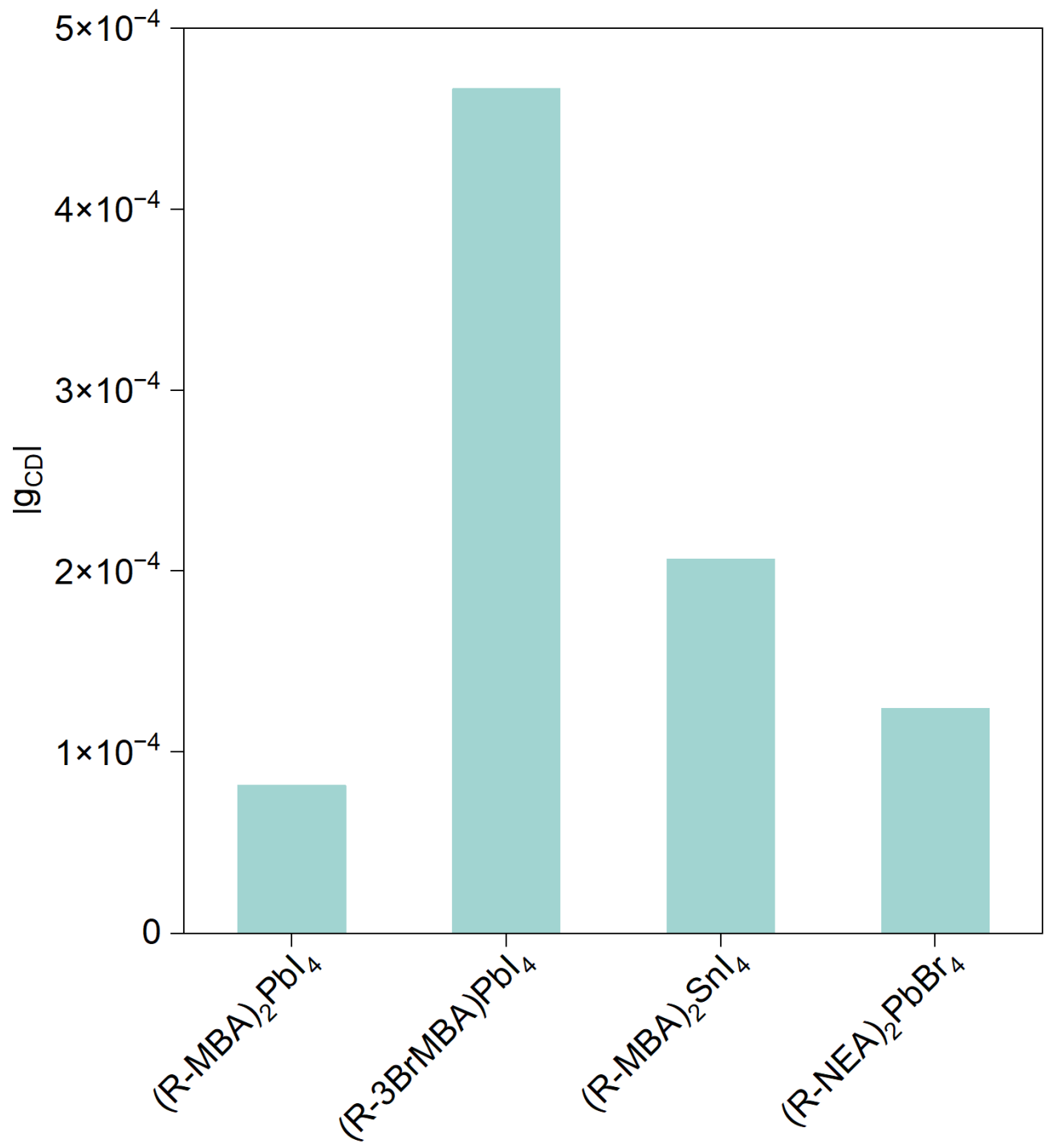

Fig. S21. Dissymmetry factor (g_{CD}). g_{CD} for various CMHS compositions calculated at the wavelength corresponding to the first major peak in the CD spectrum.

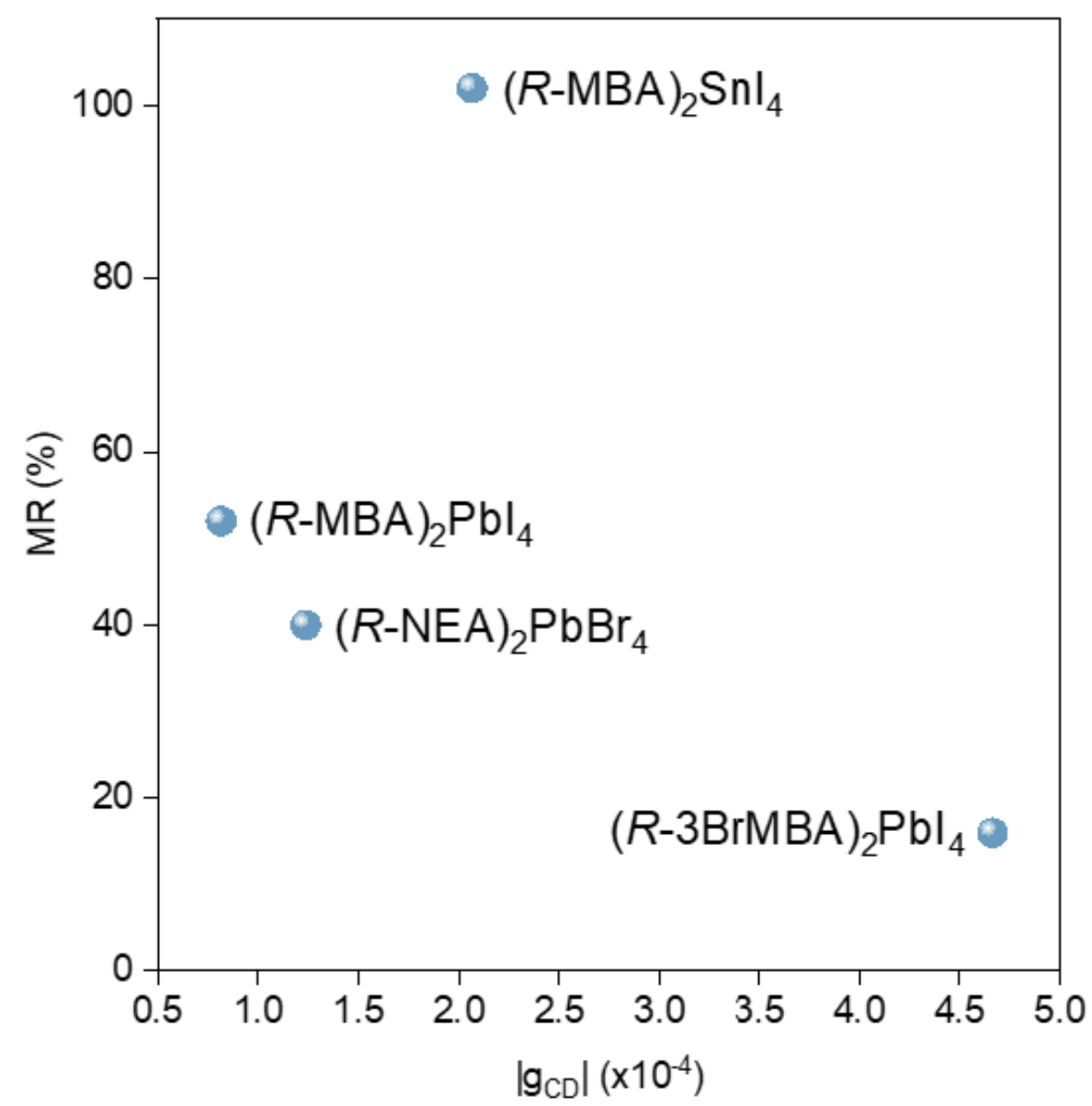

Fig. S22. MR as a function of g_{CD}. Plot of g_{CD} vs. MR for various CMHS.

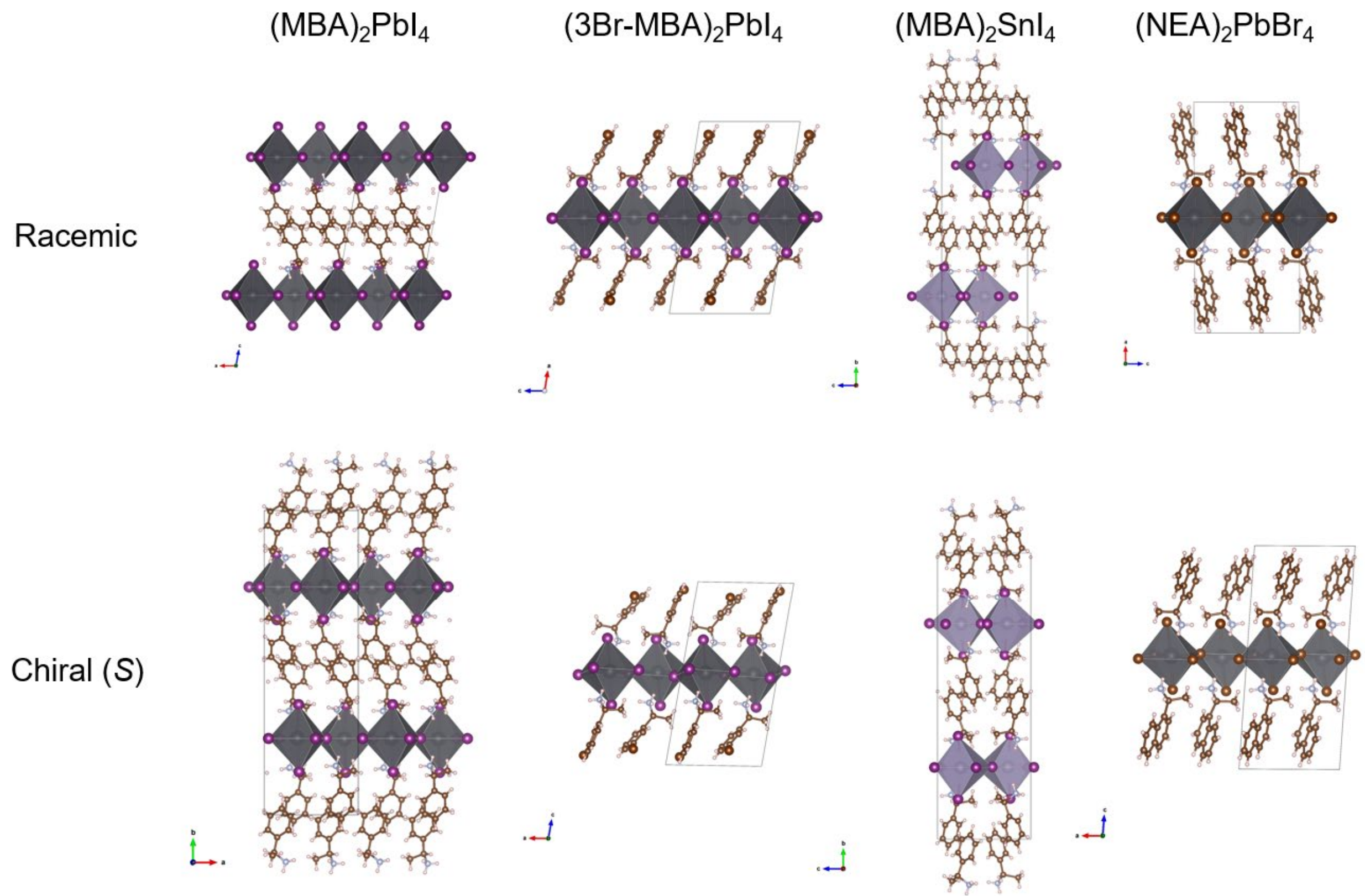

Fig. S23. CMHS crystal structures. Racemic and chiral crystal structures of various CMHS compositions used for CCM calculation.

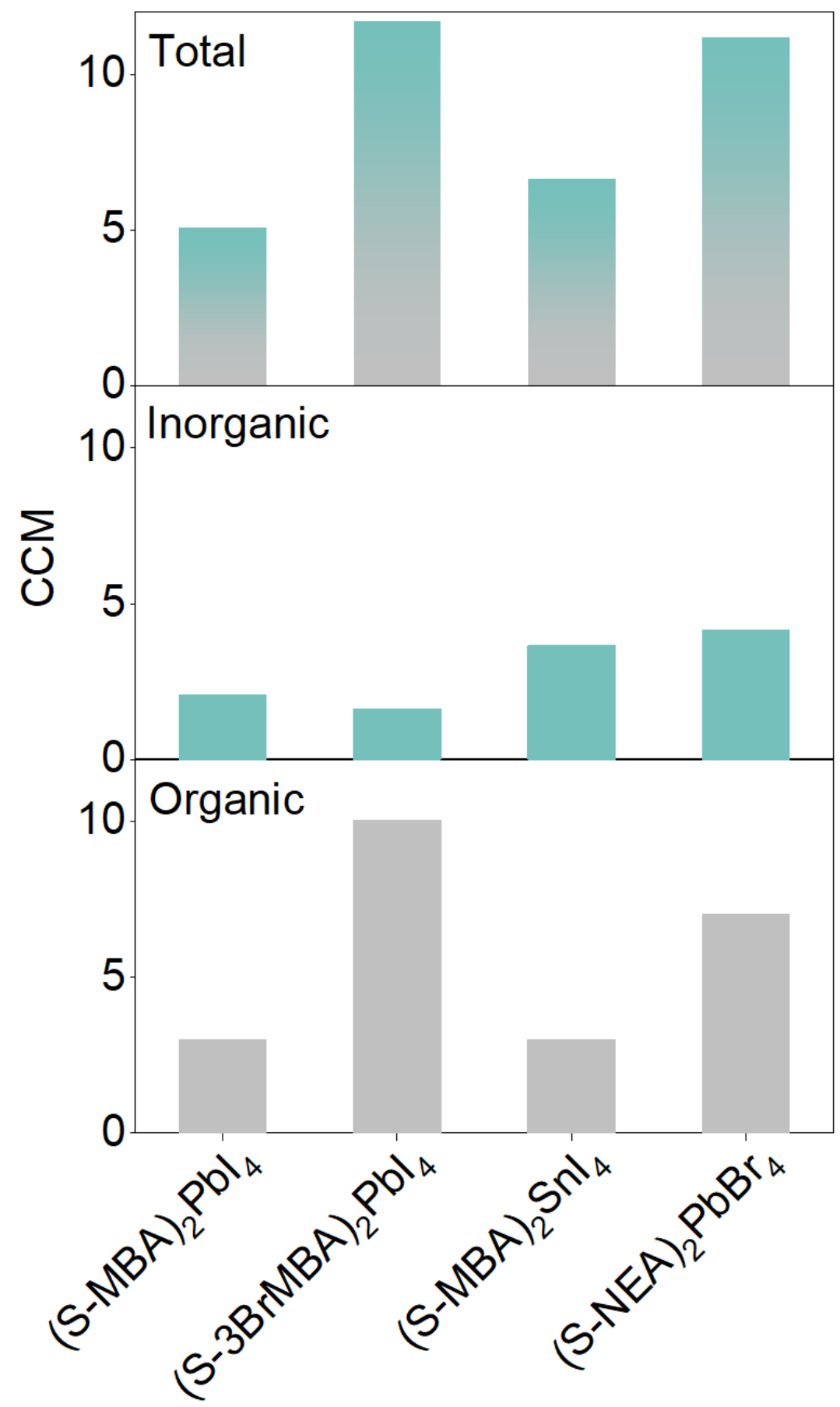

Fig. S24. Continuous chirality measure. CCM for the organic cation, inorganic framework along with total CCM for various CMHS compositions.

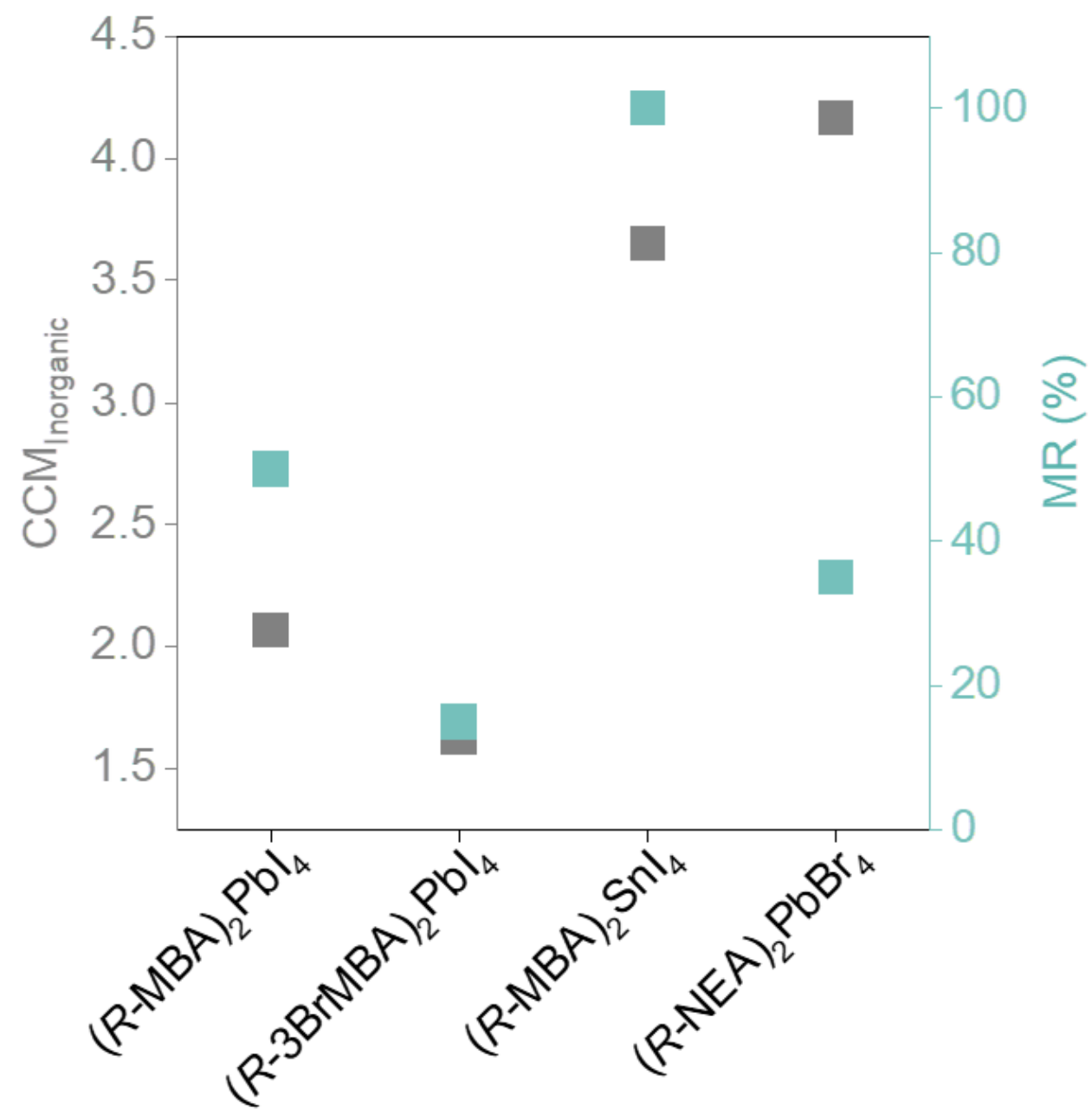

Fig. S25. Structure-property correlation. Comparison between inorganic CCM and MR for various CMHS compositions.

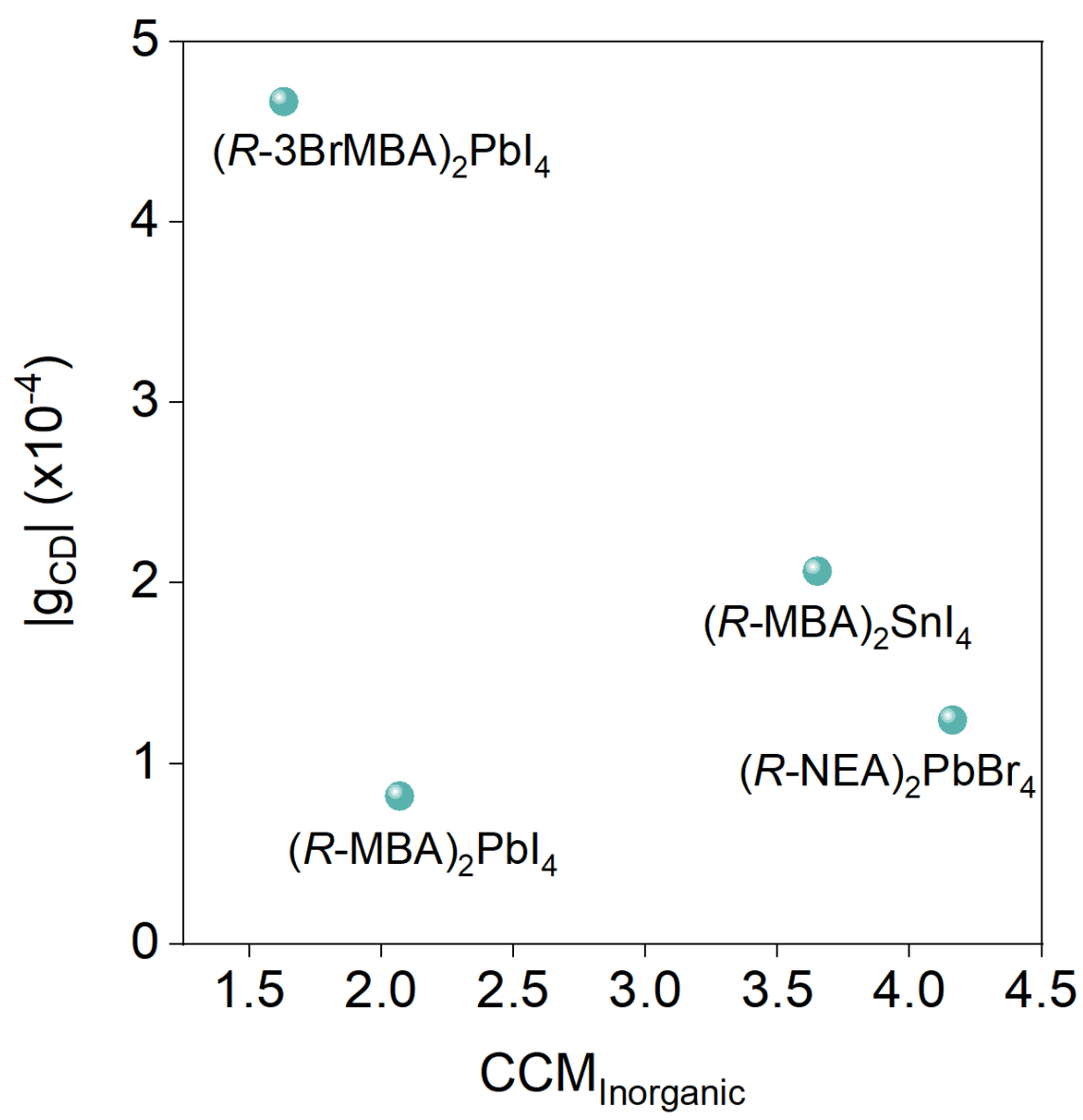

Fig. S26. g_{CD} as a function of CCM. Plot of g_{CD} vs. CCM of inorganic lattice.

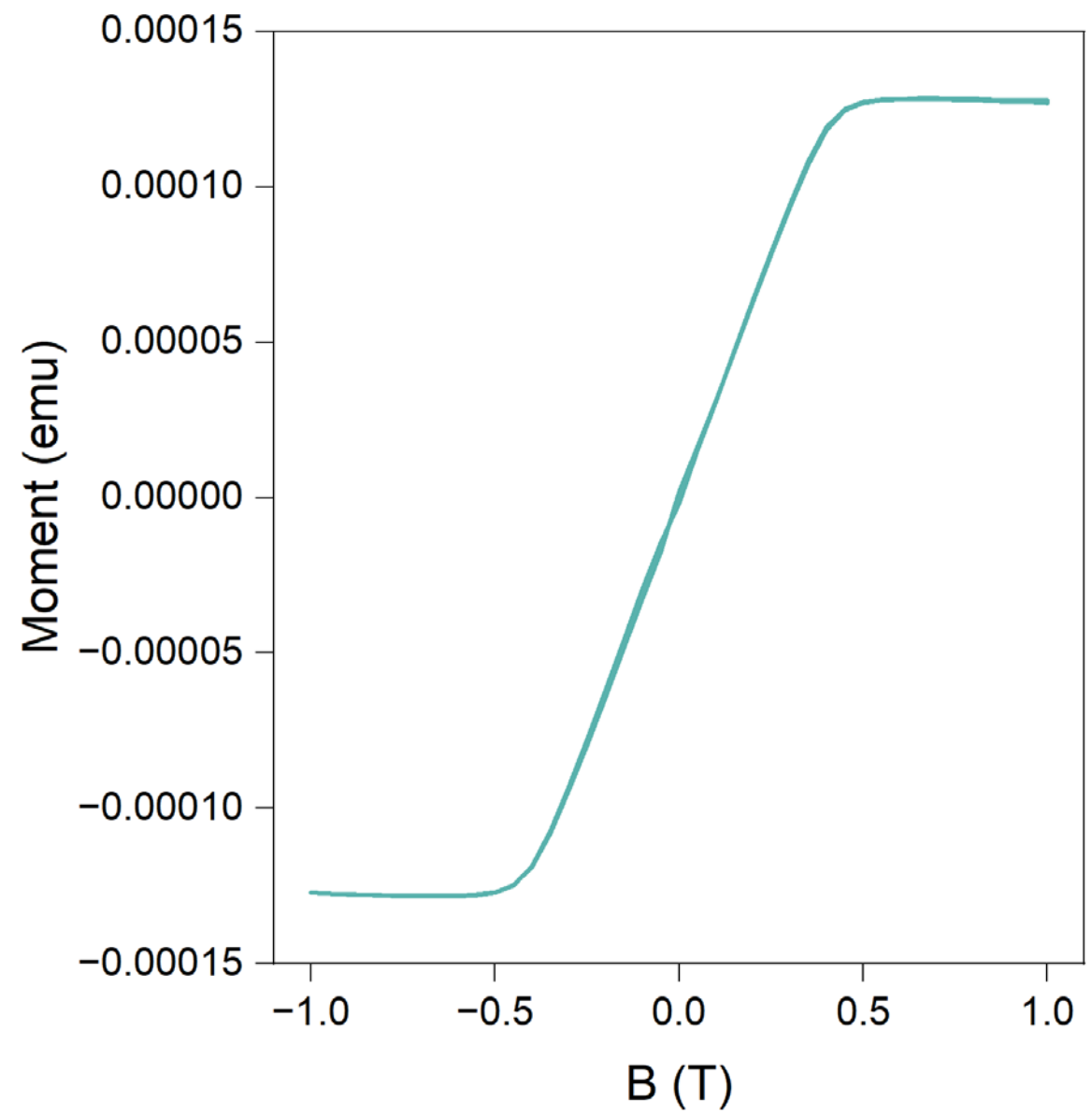

Fig. S27. Field-dependent hysteresis. Hysteresis of the FM (Ni) electrode.

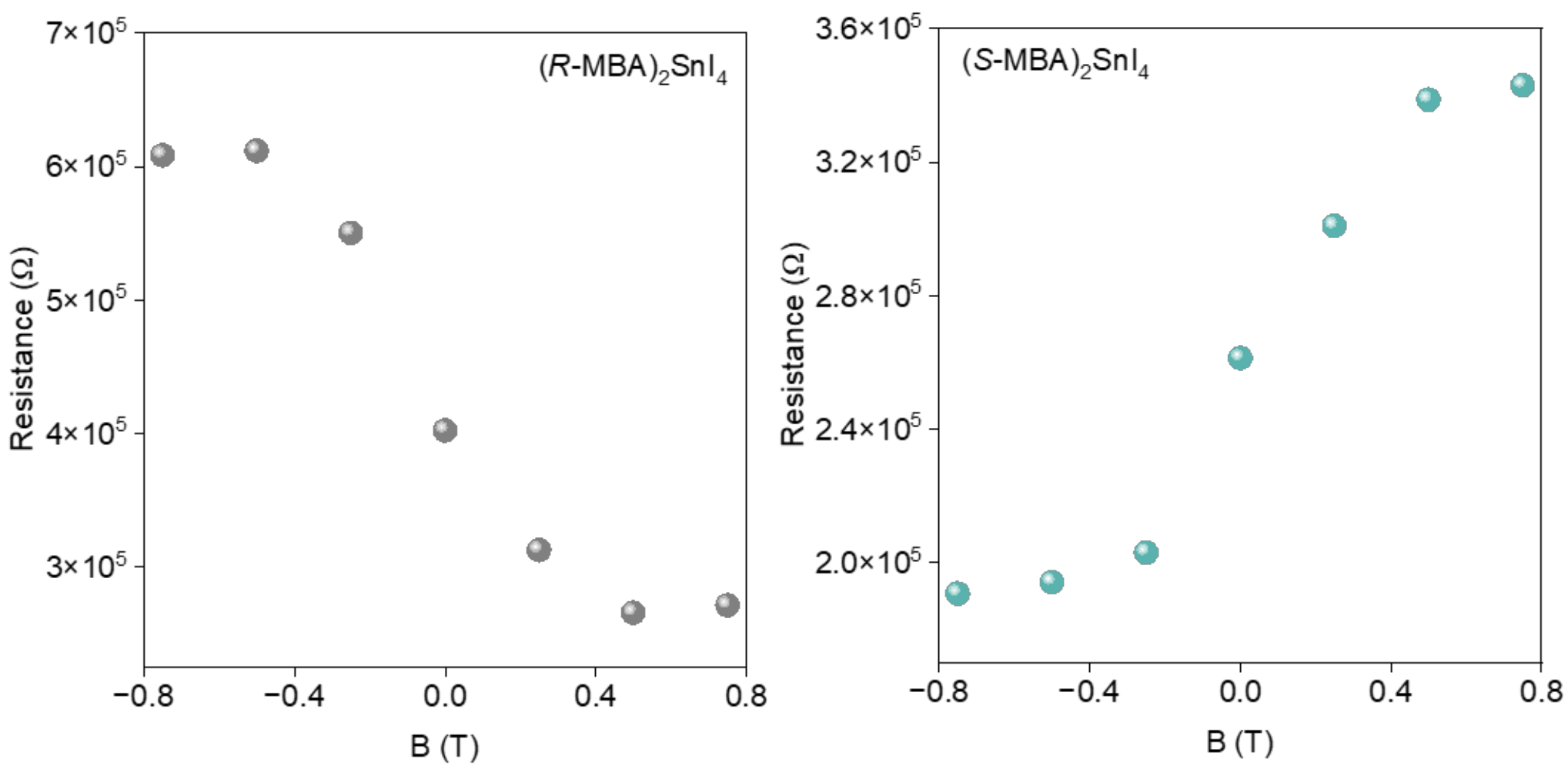

Fig. S28. Field-dependent resistance. (*R/S*-MBA)$_2$SnI$_4$ spin valve at -0.25 V.

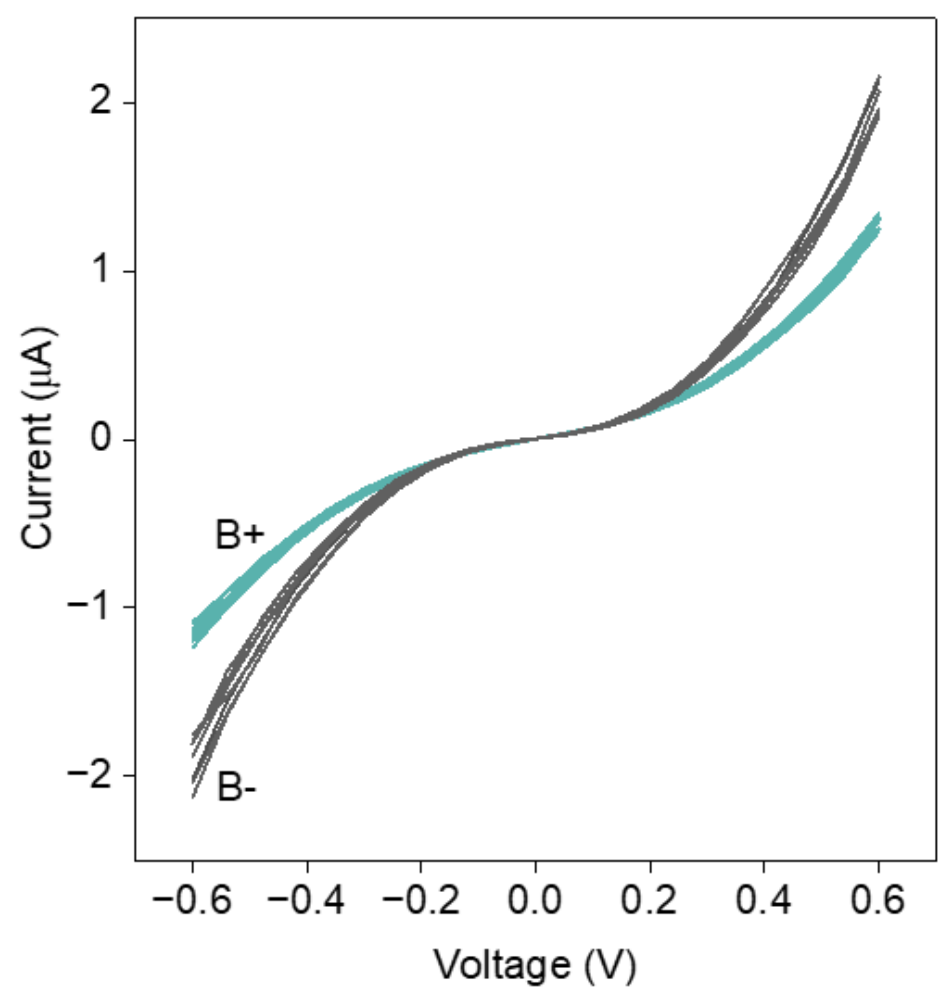

Fig. S29. Stability of current-voltage characteristics. Multiple cycles of I-Vs for spin valve (FM/Al_2O_3/CMHS/Au) employing (*S*-NEA)$_2$PbBr$_4$ CMHS under out of plane magnetic field (B± =±1 T).

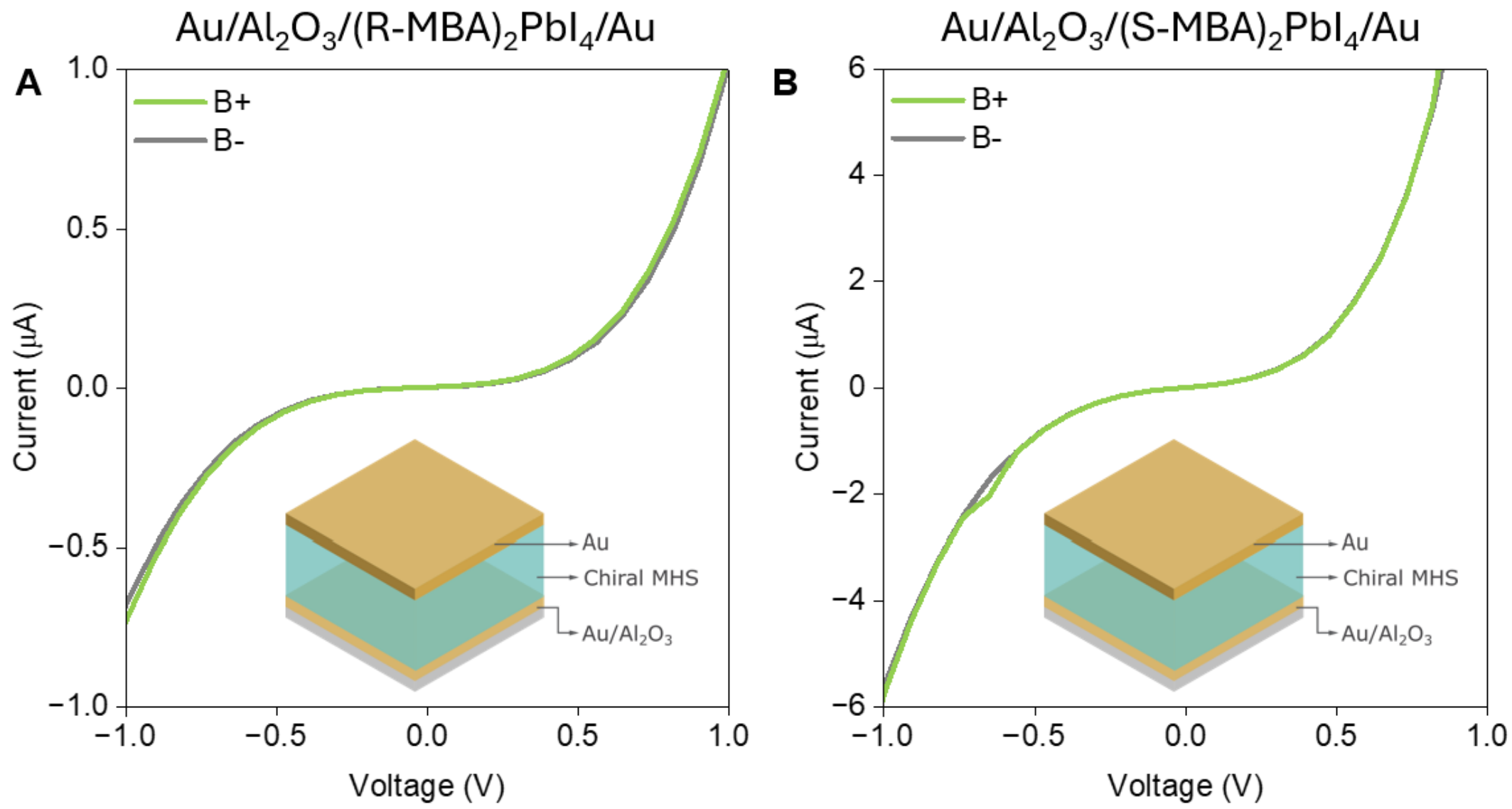

Fig. S30. Current-voltage characteristics. (**A**) Au/Al_2O_3/(*R*-MBA)$_2$$PbI_4$/Au. (**B**) Au/$Al_2O_3$/(*S*-MBA)$_2$$PbI_4$/Au showing negligible difference between opposite magnetic fields.

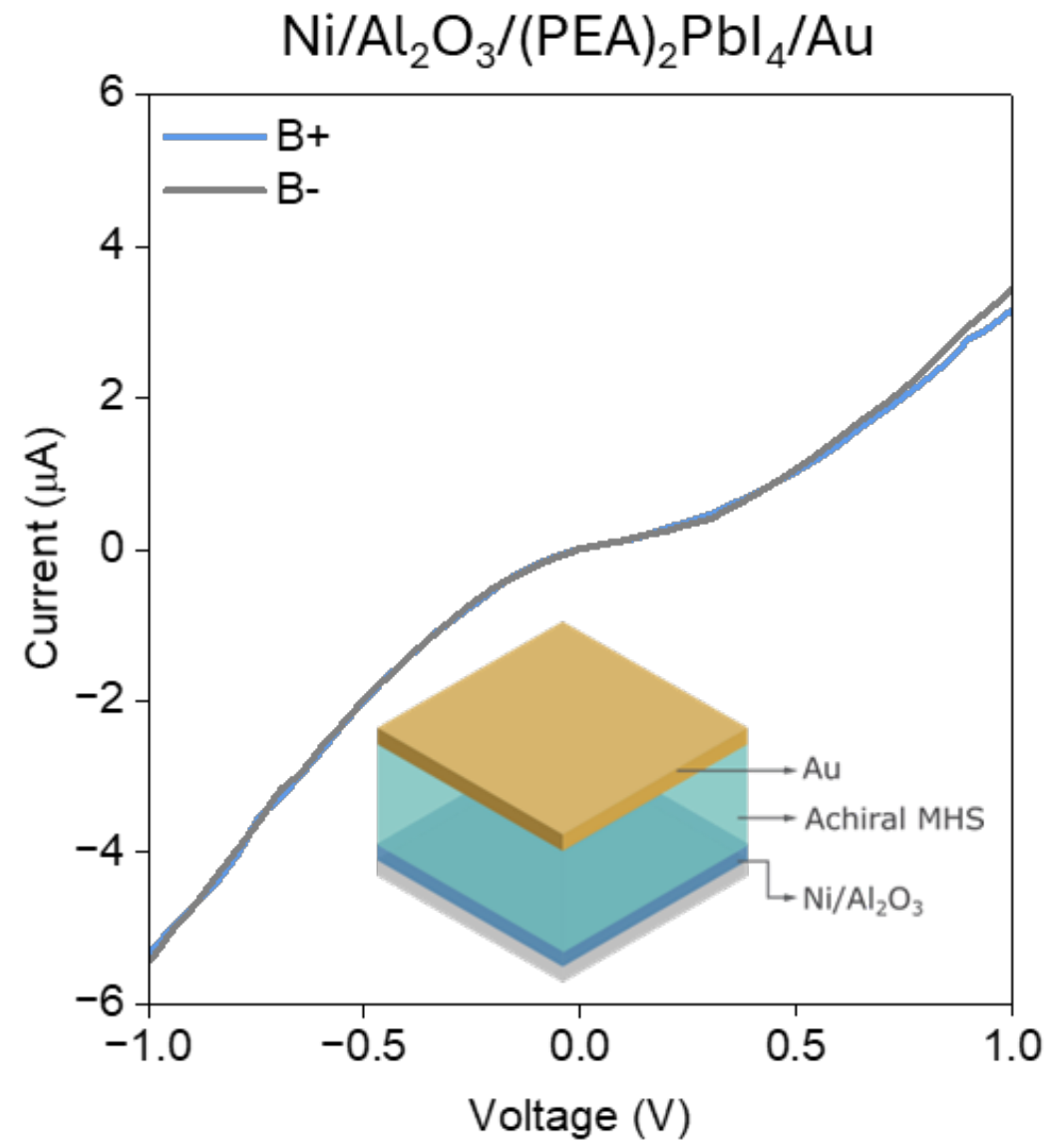

Fig. S31. Current-voltage characteristics. Ni/Al_2O_3/$(PEA)_2PbI_4$/Au spin valve showing negligible response to magnetic field.

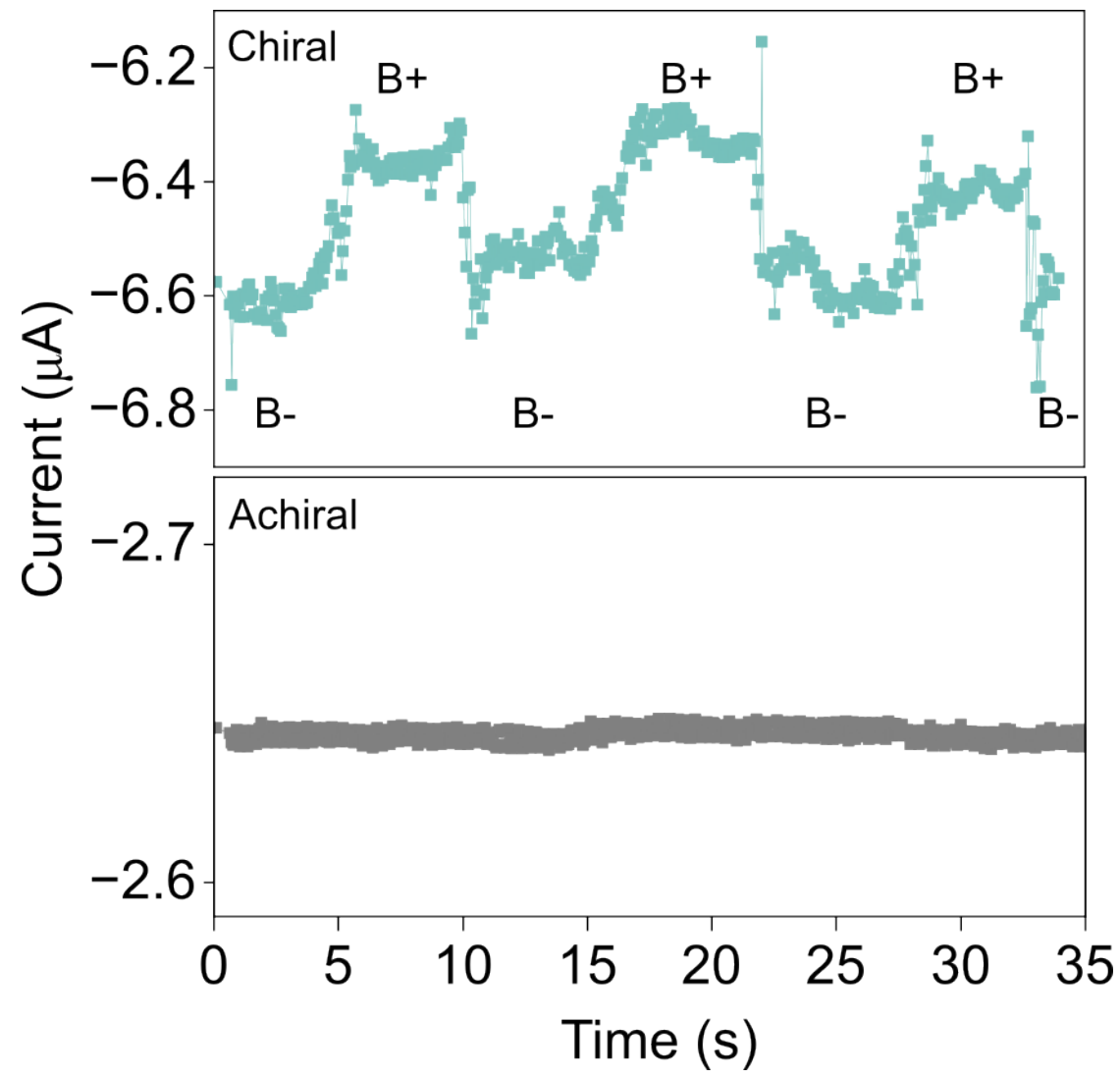

Fig. S32. Current-time *(I-t)* response. *I-t* for chiral (CMHS) and achiral $(PEA)_2PbI_4$ spin valves at -0.5 V recorded by flipping the permanent magnet during operation. x-axis scale is adjusted for plotting.

REFERENCES

1. M. K. Jana, R. Song, H. Liu, D. R. Khanal, S. M. Janke, R. Zhao, C. Liu, Z. Valy Vardeny, V. Blum, D. B. Mitzi, Organic-to-inorganic structural chirality transfer in a 2D hybrid perovskite and impact on Rashba-Dresselhaus spin-orbit coupling. *Nat. Commun.* **11**, 4699 (2020).

2. M. A. Haque, M. C. Beard, Spin effects in metal halide perovskite semiconductors. *Nanoscale* **17**, 9895–9906 (2025).

3. B. Göhler, V. Hamelbeck, T. Z. Markus, M. Kettner, G. F. Hanne, Z. Vager, R. Naaman, H. Zacharias, Spin selectivity in electron transmission through self-assembled monolayers of double-stranded DNA. *Science* **331**, 894–897 (2011).

4. C. Chen, L. Gao, W. Gao, C. Ge, X. Du, Z. Li, Y. Yang, G. Niu, J. Tang, Circularly polarized light detection using chiral hybrid perovskite. *Nat. Commun.* **10**, 1927 (2019).

5. M. P. Hautzinger, X. Pan, S. C. Hayden, J. Y. Ye, Q. Jiang, M. J. Wilson, A. J. Phillips, Y. Dong, E. K. Raulerson, I. A. Leahy, C. S. Jiang, J. L. Blackburn, J. M. Luther, Y. Lu, K. Jungjohann, Z. V. Vardeny, J. J. Berry, K. Alberi, M. C. Beard, Room-temperature spin injection across a chiral perovskite/III-V interface. *Nature* **631**, 307–312 (2024).

6. Q. Qian, H. Ren, J. Zhou, Z. Wan, J. Zhou, X. Yan, J. Cai, P. Wang, B. Li, Z. Sofer, B. Li, X. Duan, X. Pan, Y. Huang, X. Duan, Chiral molecular intercalation superlattices. *Nature* **606**, 902–908 (2022).

7. B. P. Bloom, Y. Paltiel, R. Naaman, D. H. Waldeck, Chiral induced spin selectivity. *Chem. Rev.* **124**, 1950–1991 (2024).

8. M. K. Jana, R. Song, Y. Xie, R. Zhao, P. C. Sercel, V. Blum, D. B. Mitzi, Structural descriptor for enhanced spin-splitting in 2D hybrid perovskites. *Nat. Commun.* **12**, 4982 (2021).

9. J. Ahn, E. Lee, J. Tan, W. Yang, B. Kim, J. Moon, A new class of chiral semiconductors: Chiral-organic-molecule-incorporating organic–inorganic hybrid perovskites. *Mater. Horiz.* **4**, 851–856 (2017).

10. M. A. Haque, A. Grieder, S. P. Harvey, R. Brunecky, J. Y. Ye, B. Addison, J. Zhang, Y. Dong, Y. Xie, M. P. Hautzinger, H. H. Walpitage, K. Zhu, J. L. Blackburn, Z. V. Vardeny, D. B. Mitzi, J. J. Berry, S. R. Marder, Y. Ping, M. C. Beard, J. M. Luther, Remote chirality transfer in low-dimensional hybrid metal halide semiconductors. *Nat. Chem.* **17**, 29–37 (2025).

11. Y. Dong, A. McConnell, M. P. Hautzinger, M. A. Haque, A. H. Comstock, P. M. Theiler, J. M. Luther, P. C. Sercel, D. Sun, M. C. Beard, Ultrafast inverse chirality-induced spin selectivity observed by THz emission. *Science* **390**, 595–600 (2025).

12. D. D. Tang, C.-F. Pai, *Magnetic Memory Technology* (The Institute of Electrical and Electronic Engineers Incorporation, 2020).

13. S. H. Tirion, B. J. van Wees, Mechanism for electrostatically generated magnetoresistance in chiral systems without spin-dependent transport. *ACS Nano* **18**, 6028–6037 (2024).

14. Y. Adhikari, T. Liu, H. Wang, Z. Hua, H. Liu, E. Lochner, P. Schlottmann, B. Yan, J. Zhao, P. Xiong, Interplay of structural chirality, electron spin and topological orbital in chiral molecular spin valves. *Nat. Commun.* **14**, 5163 (2023).

15. Y. Liu, R. Shrestha, K. Denisov, D. Ayala, M. van Schilfgaarde, W. Nie, I. Žutić, Unconventional spintronics from chiral perovskites. *Adv. Funct. Mater.* **35**, e09127 (2025).

16. T. Liu, P. S. Weiss, Spin polarization in transport studies of chirality-induced spin selectivity. *ACS Nano* **17**, 19502–19507 (2023).

17. H. Lu, J. Wang, C. Xiao, X. Pan, X. Chen, R. Brunecky, J. J. Berry, K. Zhu, M. C. Beard, Z. V. Vardeny, Spin-dependent charge transport through 2D chiral hybrid lead-iodide perovskites. *Sci. Adv.* **5**, eaay0571 (2019).

18. M.-G. Kang, I.-K. Hwang, H.-C. Kyung, J. Kang, D. Han, S. Lee, J. Kwon, K.-J. Lee, J. Yeom, B.-G. Park, Room temperature chiral magnetoresistance in a chiral-perovskite-based perpendicular spin valve. *APL Mater.* **12**, 081118 (2024).

19. A. Abherve, N. Mercier, A. Kumar, T. K. Das, J. Even, C. Katan, M. Kepenekian, Chirality versus symmetry: Electron's spin selectivity in nonpolar chiral lead-bromide perovskites. *Adv. Mater.* **35**, e2305784 (2023).

20. S. Dan, S. Paramanik, A. J. Pal, Why mixed halides in 2D chiral perovskites weaken chirality-induced spin selectivity. *ACS Nano* **18**, 35644–35653 (2024).

21. Y.-H. Kim, Y. Zhai, H. Lu, X. Pan, C. Xiao, E. A. Gaulding, S. P. Harvey, J. J. Berry, Z. V. Vardeny, J. M. Luther, M. C. Beard, Chiral-induced spin selectivity enables a room-temperature spin light-emitting diode. *Science* **371**, 1129–1133 (2021).

22. G. Jang, D. Y. Jo, S. Ma, J. Lee, J. Son, C. U. Lee, W. Jeong, S. Yang, J. H. Park, H. Yang, J. Moon, Core-shell perovskite quantum dots for highly selective room-temperature spin light-emitting diodes. *Adv. Mater.* **36**, e2309335 (2024).

23. B. P. Bloom, V. Kiran, V. Varade, R. Naaman, D. H. Waldeck, Spin selective charge transport through cysteine capped CdSe quantum dots. *Nano Lett.* **16**, 4583–4589 (2016).

24. M. F. Khan, S. Rehman, M. A. Rehman, R. U. Rehman Sagar, D.-K. Kim, H. M. Waseem Khalil, P. A. Shinde, N. U. Hassan, P. R. Sharma, J. Eom, S. C. Jun, Multi-heterostructured spin-valve junction of vertical FLG/MoSe2/FLG. *APL Mater.* **8**, 071104 (2020).

25. T. Liu, X. Wang, H. Wang, G. Shi, F. Gao, H. Feng, H. Deng, L. Hu, E. Lochner, P. Schlottmann, S. von Molnar, Y. Li, J. Zhao, P. Xiong, Linear and nonlinear two-terminal spin-valve effect from chirality-induced spin selectivity. *ACS Nano* **14**, 15983–15991 (2020).

26. S. O. Valenzuela, D. J. Monsma, C. M. Marcus, V. Narayanamurti, M. Tinkham, Spin polarized tunneling at finite bias. *Phys. Rev. Lett.* **94**, 196601 (2005).

27. G. A. Rikken, N. Avarvari, Comparing electrical magnetochiral anisotropy and chirality-induced spin selectivity. *J. Phys. Chem. Lett.* **14**, 9727–9731 (2023).

28. Y. Utsumi, O. Entin-Wohlman, A. Aharony, Spin selectivity through time-reversal symmetric helical junctions. *Phys. Rev. B* **102**, 035445 (2020).

29. P. M. Theiler, M. C. Beard, Equilibrium spin polarization arising from chirality. *Newton* **2**, 100480 (2026).

30. G. Schmidt, D. Ferrand, L. W. Molenkamp, A. T. Filip, B. J. van Wees, Fundamental obstacle for electrical spin injection from a ferromagnetic metal into a diffusive semiconductor. *Phys. Rev. B* **62**, R4790–R4793 (2000).

31. Y. Dong, M. P. Hautzinger, M. A. Haque, M. C. Beard, Chirality-induced spin selectivity in hybrid organic-inorganic perovskite semiconductors. *Annu. Rev. Phys. Chem.* **76**, 519–537 (2025).

32. S. Ding, Y. Tian, Y. Li, W. Mi, H. Dong, X. Zhang, W. Hu, D. Zhu, Inverse magnetoresistance in polymer spin valves. *ACS Appl. Mater. Interfaces* **9**, 15644–15651 (2017).

33. J. J. Versluijs, M. A. Bari, J. M. D. Coey, Magnetoresistance of half-metallic oxide nanocontacts. *Phys. Rev. Lett.* **87**, 026601 (2001).

34. W. Zhu, H. Lin, F. Yan, C. Hu, Z. Wang, L. Zhao, Y. Deng, Z. R. Kudrynskyi, T. Zhou, Z. D. Kovalyuk, Y. Zheng, A. Patane, I. Zutic, S. Li, H. Zheng, K. Wang, Large tunneling magnetoresistance in van der Waals ferromagnet/semiconductor heterojunctions. *Adv. Mater.* **33**, e2104658 (2021).

35. F. Volmer, M. Drögeler, T. Pohlmann, G. Güntherodt, C. Stampfer, B. Beschoten, Contact-induced charge contributions to non-local spin transport measurements in Co/MgO/graphene devices. *2D Mater.* **2**, 024001 (2015).

36. F. Volmer, M. Drögeler, E. Maynicke, N. von den Driesch, M. L. Boschen, G. Güntherodt, B. Beschoten, Role of MgO barriers for spin and charge transport in Co/MgO/graphene nonlocal spin-valve devices. *Phys. Rev. B* **88**, 161405 (2013).

37. J. M. Slaughter, Materials for magnetoresistive random access memory. *Annu. Rev. Mat. Res.* **39**, 277–296 (2009).

38. M. M. C. Souza, R. C. Sousa, C. Ducruet, S. Auffret, B. Dieny, Metallic Mg insertion in rf deposited MgO barrier. *J. Appl. Phys.* **107**, 09C702 (2010).

39. R. W. Dave, G. Steiner, J. M. Slaughter, J. J. Sun, B. Craigo, S. Pietambaram, K. Smith, G. Grynkewich, M. DeHerrera, J. Akerman, S. Tehrani, MgO-based tunnel junction material for high-speed toggle magnetic random access memory. *IEEE Trans. Magn.* **42**, 1935–1939 (2006).

40. S. Yuasa, T. Nagahama, A. Fukushima, Y. Suzuki, K. Ando, Giant room-temperature magnetoresistance in single-crystal Fe/MgO/Fe magnetic tunnel junctions. *Nat. Mater.* **3**, 868–871 (2004).

41. D. Pantel, S. Goetze, D. Hesse, M. Alexe, Reversible electrical switching of spin polarization in multiferroic tunnel junctions. *Nat. Mater.* **11**, 289–293 (2012).

42. T. Kado, Large room-temperature inverse magnetoresistance in tunnel junctions with a Fe_3O_4 electrode. *Appl. Phys. Lett.* **92**, 092502 (2008).

43. T. Kado, H. Saito, K. Ando, Room-temperature magnetoresistance in magnetic tunnel junctions with Fe_3O_4 electrode. *J. Appl. Phys.* **101**, 09J511 (2007).

44. K. Z. Suzuki, T. Izumi, X. Zhang, A. Sugihara, S.-T. Pham, H. Taka, S. Sato, H. Isobe, S. Mizukami, Room temperature magnetoresistance in an organic spin valve with an aromatic hydrocarbon macrocycle. *APL Mater.* **5**, 046101 (2017).

45. M. Cubukcu, M. B. Martin, P. Laczkowski, C. Vergnaud, A. Marty, J. P. Attané, P. Seneor, A. Anane, C. Deranlot, A. Fert, S. Auffret, C. Ducruet, L. Notin, L. Vila, M. Jamet, Ferromagnetic tunnel contacts to graphene: Contact resistance and spin signal. *J. Appl. Phys.* **117**, 083909 (2015).

46. S. Yuasa, T. Sato, E. Tamura, Y. Suzuki, H. Yamamori, K. Ando, T. Katayama, Magnetic tunnel junctions with single-crystal electrodes: A crystal anisotropy of tunnel magneto-resistance. *Europhys. Lett.* **52**, 344 (2000).

47. R. Gupta, A. Balo, R. Garg, A. K. Mondal, K. B. Ghosh, P. C. Mondal, The chirality-induced spin selectivity effect in asymmetric spin transport: From solution to device applications. *Chem. Sci.* **15**, 18751–18771 (2024).

48. O. Ben Dor, S. Yochelis, A. Radko, K. Vankayala, E. Capua, A. Capua, S. H. Yang, L. T. Baczewski, S. S. Parkin, R. Naaman, Y. Paltiel, Magnetization switching in ferromagnets by adsorbed chiral molecules without current or external magnetic field. *Nat. Commun.* **8**, 14567 (2017).

49. K. Banerjee-Ghosh, O. Ben Dor, F. Tassinari, E. Capua, S. Yochelis, A. Capua, S.-H. Yang, S. S. P. Parkin, S. Sarkar, L. Kronik, L. T. Baczewski, R. Naaman, Y. Paltiel, Separation of enantiomers by their enantiospecific interaction with achiral magnetic substrates. *Science* **360**, 1331–1334 (2018).

50. M. Julliere, Tunneling between ferromagnetic films. *Phys. Lett. A* **54**, 225–226 (1975).

51. J. N. Chazalviel, Y. Yafet, Theory of the spin polarization of field-emitted electrons from nickel. *Phys. Rev. B* **15**, 1062–1071 (1977).

52. J. C. Slonczewski, Conductance and exchange coupling of two ferromagnets separated by a tunneling barrier. *Phys. Rev. B* **39**, 6995–7002 (1989).

53. J. M. MacLaren, X. G. Zhang, W. H. Butler, Validity of the Julliere model of spin-dependent tunneling. *Phys. Rev. B* **56**, 11827–11832 (1997).

54. X. G. Zhang, W. H. Butler, Band structure, evanescent states, and transport in spin tunnel junctions. *J. Phys. Condens. Matter* **15**, R1603 (2003).

55. A. H. MacDonald, T. Jungwirth, M. Kasner, Temperature dependence of itinerant electron junction magnetoresistance. *Phys. Rev. Lett.* **81**, 705–708 (1998).

56. Q. Q. Shu, W. G. Ma, Barrier parameter variation in Al-Al_2O_3-metal tunnel junctions. *Appl. Phys. Lett.* **61**, 2542–2544 (1992).

57. J. M. Abendroth, K. M. Cheung, D. M. Stemer, M. S. El Hadri, C. Zhao, E. E. Fullerton, P. S. Weiss, Spin-dependent ionization of chiral molecular films. *J. Am. Chem. Soc.* **141**, 3863–3874 (2019).

58. S. Ghosh, S. Mishra, E. Avigad, B. P. Bloom, L. T. Baczewski, S. Yochelis, Y. Paltiel, R. Naaman, D. H. Waldeck, Effect of chiral molecules on the electron's spin wavefunction at interfaces. *J. Phys. Chem. Lett.* **11**, 1550–1557 (2020).

59. P. M. Theiler, C. Ritz, R. Hofmann, A. Stemmer, Detection of a chirality-induced spin selective quantum capacitance in alpha-helical peptides. *Nano Lett.* **23**, 8280–8287 (2023).

60. P. M. Theiler, S. Driessen, M. C. Beard, PT symmetry enforced twin exchange as the origin of chirality-induced spin selectivity. *Sci. Adv.* **12**, eaec7069 (2026).

61. J. Fransson, Charge redistribution and spin polarization driven by correlation induced electron exchange in chiral molecules. *Nano Lett.* **21**, 3026–3032 (2021).

62. G. H. Fecher, J. Kübler, C. Felser, Chirality in the solid state: Chiral crystal structures in chiral and achiral space groups. *Materials* **15**, 5812 (2022).

63. S. Liu, M. Kepenekian, S. Bodnar, S. Feldmann, M. W. Heindl, N. Fehn, J. Zerhoch, A. Shcherbakov, A. Pöthig, Y. Li, U. W. Paetzold, A. Kartouzian, I. D. Sharp, C. Katan, J. Even, F. Deschler, Bright circularly polarized photoluminescence in chiral layered hybrid lead-halide perovskites. *Sci. Adv.* **9**, eadh5083 (2023).

64. H. Lu, C. Xiao, R. Song, T. Li, A. E. Maughan, A. Levin, R. Brunecky, J. J. Berry, D. B. Mitzi, V. Blum, M. C. Beard, Highly distorted chiral two-dimensional tin iodide perovskites for spin polarized charge transport. *J. Am. Chem. Soc.* **142**, 13030–13040 (2020).

65. P. C. Sercel, M. P. Hautzinger, R. Song, V. Blum, M. C. Beard, Optical activity of chiral excitons. *Adv. Mater.* **37**, e2415901 (2025).

66. M. Fortino, A. Mattoni, A. Pietropaolo, Atomistic modeling of metal–ligand chirality transfer and chiroptical properties of lead and tin hybrid perovskites. *J. Mater. Chem. C* **11**, 9135–9143 (2023).

67. M. Taddei, J. Zhang, M. A. Haque, C. McLeod, S. P. Harvey, Y. Dong, L. T. Schelhas, S. Barlow, J. L. Blackburn, J. M. Luther, S. R. Marder, M. C. Beard, Symmetry breaking

induced by chiral phosphonic acids in a 2D tin-halide perovskite. *J. Am. Chem. Soc.* **147**, 36642–36649 (2025).

68. I. Tuvi-Arad, Y. Shalit, G. Alon, CSM software: Continuous symmetry and chirality measures for quantitative structural analysis. *J. Chem. Inf. Model.* **64**, 5375–5380 (2024).

69. S. Alvarez, P. Alemany, D. Avnir, Continuous chirality measures in transition metal chemistry. *Chem. Soc. Rev.* **34**, 313–326 (2005).

70. A. Grieder, S. Tu, Y. Ping, Relation of continuous chirality measure to spin and orbital polarization, and chiroptical properties in solids. *Adv. Optical Mater.* **13**, e01190 (2025).

71. N. Goren, P. Pandurangan, Y. Eisenberg-Domovich, S. Yochelis, N. Keren, J. P. Ansermet, R. Naaman, O. Livnah, N. Ashkenasy, Y. Paltiel, Coupling between electrons' spin and proton transfer in chiral biological crystals. *Proc. Natl. Acad. Sci. U.S.A.* **122**, e2500584122 (2025).

72. V. T. Pham, M. Cosset-Chéneau, A. Brenac, O. Boulle, A. Marty, J.-P. Attané, L. Vila, Evidence of interfacial asymmetric spin scattering at ferromagnet-Pt interfaces. *Phys. Rev. B* **103**, L201403 (2021).

73. M. A. Haque, P. M. Theiler, I. A. Leahy, S. P. Harvey, J. Tan, M. P. Hautzinger, M. Taddei, A. McConnell, A. Grieder, A. H. Comstock, Y. Dong, K. Alberi, Y. Ping, P. C. Sercel, J. M. Luther, D. Sun, M. C. Beard, Dataset for chirality-induced magnetoresistance in hybrid organic-inorganic perovskite semiconductors. Zenodo, (2026); https://doi.org/10.5281/zenodo.19702071.

74. M. Johnson, R. H. Silsbee, Interfacial charge-spin coupling: Injection and detection of spin magnetization in metals. *Phys. Rev. Lett.* **55**, 1790–1793 (1985).

75. A. Scholl, M. Liberati, E. Arenholz, H. Ohldag, J. Stöhr, Creation of an antiferromagnetic exchange spring. *Phys. Rev. Lett.* **92**, 247201 (2004).

76. R. Pan, X. Tang, X. Wang, Y. Liu, L. Huang, Y. Wang, Z. Wang, X. Zhou, Impact of chiral spinterfaces on magneto-photoluminescence effects for chiral lead halide perovskites. *ACS Appl. Mater. Interfaces* **16**, 2964–2971 (2024).

77. B. Li, Y. Li, W. Yuan, X. Zhang, S. Tao, H. Zhan, Z.-G. Yu, K. Wang, J. Liu, L. Wang, C. Qin, Chiral quasi-2D perovskites based single junction spin-light-emitting diodes. *Adv. Funct. Mater.* **35**, 2415433 (2025).

78. W. H. Butler, Tunneling magnetoresistance from a symmetry filtering effect. *Sci. Technol. Adv. Mater.* **9**, 014106 (2008).

79. J. G. Simmons, Generalized formula for the electric tunnel effect between similar electrodes separated by a thin insulating film. *J. Appl. Phys.* **34**, 1793–1803 (1963).
